# Chromatin accessibility dynamics of neurogenic niche cells reveal defects in neural stem cell adhesion and migration during aging

**DOI:** 10.1038/s43587-023-00449-3

**Published:** 2023-07-13

**Authors:** Robin W. Yeo, Olivia Y. Zhou, Brian L. Zhong, Eric D. Sun, Paloma Navarro Negredo, Surag Nair, Mahfuza Sharmin, Tyson J. Ruetz, Mikaela Wilson, Anshul Kundaje, Alexander R. Dunn, Anne Brunet

**Affiliations:** 1 Department of Genetics, Stanford University, Stanford, CA USA; 2 Stanford Biophysics Program, Stanford University, Stanford, CA USA; 3 Stanford Medical Scientist Training Program, Stanford University, Stanford, CA USA; 4 Department of Chemical Engineering, Stanford University, Stanford, CA USA; 5 Biomedical Informatics Graduate Program, Stanford University, Stanford, CA USA; 6 Department of Computer Science, Stanford University, Stanford, CA USA; 7 Glenn Laboratories for the Biology of Aging, Stanford University, Stanford, CA USA; 8 Wu Tsai Neurosciences Institute, Stanford University, Stanford, CA USA

**Keywords:** Cell migration, Ageing, Neural stem cells, Cell adhesion

## Abstract

The regenerative potential of brain stem cell niches deteriorates during aging. Yet the mechanisms underlying this decline are largely unknown. Here we characterize genome-wide chromatin accessibility of neurogenic niche cells in vivo during aging. Interestingly, chromatin accessibility at adhesion and migration genes decreases with age in quiescent neural stem cells (NSCs) but increases with age in activated (proliferative) NSCs. Quiescent and activated NSCs exhibit opposing adhesion behaviors during aging: quiescent NSCs become less adhesive, whereas activated NSCs become more adhesive. Old activated NSCs also show decreased migration in vitro and diminished mobilization out of the niche for neurogenesis in vivo. Using tension sensors, we find that aging increases force-producing adhesions in activated NSCs. Inhibiting the cytoskeletal-regulating kinase ROCK reduces these adhesions, restores migration in old activated NSCs in vitro, and boosts neurogenesis in vivo. These results have implications for restoring the migratory potential of NSCs and for improving neurogenesis in the aged brain.

## Abstract (web summary)

Yeo and Zhou et al. profile whole-genome chromatin accessibility in different cell types of the adult neurogenic niche, which reveals a reversible decrease in the migration of proliferative neural stem cells in aged mice.

## Main

The adult brain contains regenerative NSC niches with progenitors that migrate to distal brain regions to generate new neurons and glial cells^1–5^. The regenerative potential of stem cell niches in the brain declines with age, and this is accompanied by a corresponding deterioration in aspects of sensory and cognitive function as well as repair ability^2,6–8^. In the subventricular zone (SVZ) neurogenic niche, quiescent NSCs (qNSCs) line the ventricular wall^9–11^. qNSCs can activate and generate progenitors and neuroblasts that migrate through the rostral migratory stream (RMS) toward the olfactory bulb (OB) to produce new neurons^12^. NSC progeny also migrate to sites of injury to mitigate damage by generating new neurons^13^ and astrocytes^14^. Both the regenerative potential and repair abilities of the SVZ neurogenic region decline with age^15–19^. Previous transcriptomic studies have uncovered defects in inflammation, signaling pathways and the cell cycle in the SVZ neurogenic niche during aging^20–23^. But the mechanisms underlying regenerative decline during aging—and how defects in migratory potential might be involved—are largely unknown.

Epigenomic changes that affect chromatin states play an important role in the regulation of cell fate^24^ and aging^25^. So far, however, epigenomic studies of NSCs have been limited to whole tissues in vivo^26,27^, developmental studies^28^ or culture systems^27,29–31^. Importantly, age-dependent epigenomic changes in different cell types of the neurogenic niche in vivo remain unknown. Such changes to the chromatin landscape of NSCs could have a longer-lasting impact on progeny and reveal features of aging that were previously undetected. Identifying chromatin changes in different cell types from the neurogenic niche during aging may also identify ways to reverse age-dependent defects and counter brain aging.

## Chromatin profiling of neurogenic niche cells during aging

**Fig. 1:**
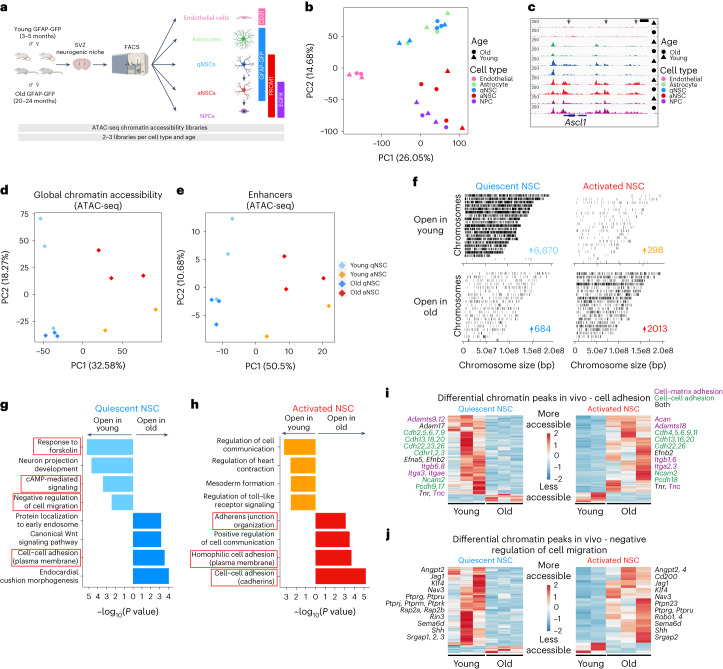
Chromatin profiling of five cell types freshly isolated from the SVZ neurogenic niche reveals opposing changes with age in quiescent and activated NSCs involving cell adhesion and migration pathways. **a**, Design for freshly isolating five cell types from the SVZ using FACS. Created with BioRender.com. **b**, PCA on all genome-wide chromatin peaks from SVZ niche cell types isolated from young (triangle) and old (circle) GFAP-GFP mice. Each dot represents a single ATAC-seq library. **c**, Genome browser (IGV) view of chromatin accessibility signal tracks from representative RPKM-normalized libraries of all SVZ niche cell types around the *Ascl1* locus. Black arrows represent sites of differentially accessible peaks that open in young aNSCs when compared to young qNSCs. Scale bar, 1 kb. **d**, PCA on all chromatin peaks from young and old qNSCs and aNSCs. Each dot represents a single ATAC-seq library. **e**, PCA on chromatin peaks that overlap with marks of enhancers (H3K27ac and p300 binding) from young and old qNSCs and aNSCs. Each dot represents a single ATAC-seq library. **b**,**d**,**e**, PCA generated using variance stabilizing transformation (VST)-normalized read counts. **f**, Chromosome-level visualization of differentially accessible ATAC-seq peaks (FDR < 0.05) that change with age in qNSC and aNSC. Each vertical bar represents a dynamic ATAC-seq peak aligned to mouse chromosomes (mouse genome *mm10*) that is differentially open in young or old NSCs. **g**,**h**, Selected GO terms for genes associated with differentially accessible ATAC-seq peaks (FDR < 0.05) that change with age in quiescent (**g**) and activated (h) NSCs generated by EnrichR and ranked by *P* value (two-sided Fisher’s exact test). ATAC-seq peaks annotated with their nearest gene using ChIPSeeker. Red boxes indicate GO terms associated with adhesion and migration. **i**,**j**, Heat maps showing accessibility levels of differential ATAC-seq peaks associated with the ‘cell adhesion’ GO category (**i**) and ‘negative regulation of cell migration’ GO category (**j**) that change with age in qNSCs and aNSCs. Selected gene names with associated differentially accessible peaks are displayed. In **i**, genes names are colored according to AmiGO as cell–matrix adhesion genes (purple), cell–cell adhesion genes (green) or both (black). TMM-normalized read counts (by EdgeR), scaled row-wise. Source data

**Extended Data Fig. 1:**
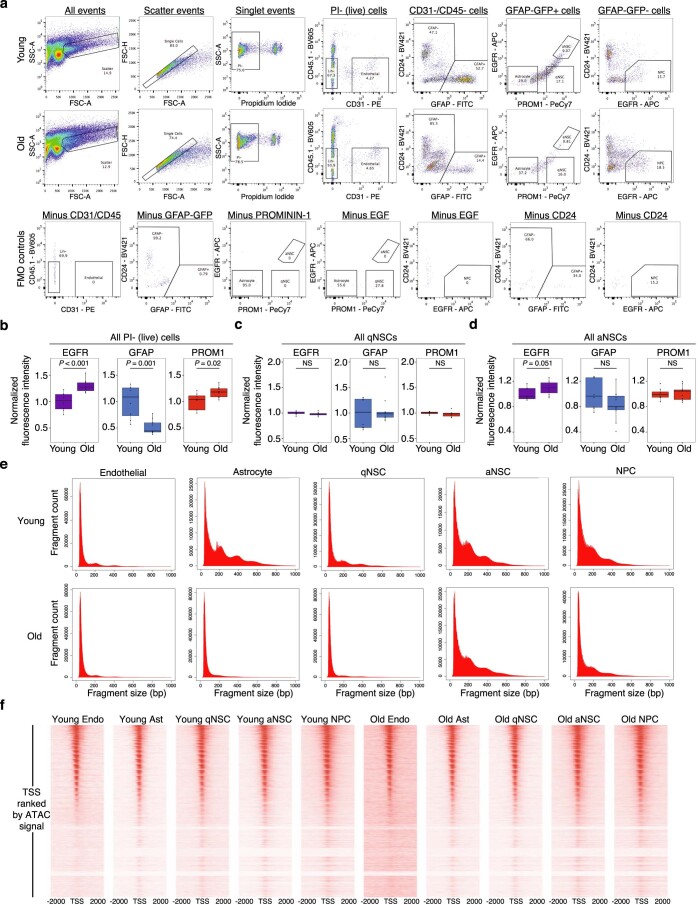
Quality control metrics for FACS and in vivo ATAC-seq libraries. **a**, FACS gating scheme used to isolate 5 cell populations from the SVZ of young and old GFAP-GFP mice. Negative controls (fluorescence minus one (FMO) controls) for each stain are indicated. Negative controls (FMO controls) were stained with all antibodies other than the one(s) for which it was the negative control. **b-d** Changes in FACS markers with age for all sorted live cells (**b**), sorted live qNSCs (**c**), and sorted live aNSCs (**d)**. Normalized scaled fluorescence intensity of indicated FACS markers (EGFR, GFAP, and PROM1) of young and old sorted cells used for generating ATAC-seq libraries from freshly isolated cells from the SVZ neurogenic niche. *n* = 8 young male and female GFAP-GFP mice and *n* = 11 old male and female GFAP-GFP mice, combined over 2 independent experiments. Data normalized to young mean of each individual experiment. Boxplots display median, and lower and upper quartiles. Minimum and maximum within 1.5× the interquartile range (whiskers) indicated. Dots outside whiskers represent outliers. Dots represent mean fluorescence intensity for an individual animal, normalized to mean of experiment. *P*-values calculated using a two-tailed Mann-Whitney test. The change in marker expression in all live cells in (**b**) likely reflects changes in cell type composition that occur with age. **e**, Insert size distribution histograms of all paired-end reads from a single representative ATAC-seq library for each of the 5 cell types freshly isolated from the SVZ of both young and old animals. **f**, Heatmap of ATAC-seq enrichment at genome-wide transcription start sites (TSSs) ± 2000 base pairs for all 5 cell types in both young and old conditions. Data from independent experiments are in Source Data. Ages of animals used provided in Source Data. Source data

To determine the impact of aging on the chromatin landscape of cells in the neurogenic niche in vivo, we generated chromatin accessibility profiles from five cell types freshly isolated from the SVZ neurogenic niche of young and old mice. We aged cohorts of transgenic mice expressing GFP driven by the human promoter for glial fibrillary acidic protein (GFAP-GFP)^32^, which, in combination with other markers, enables the isolation of five different cell types by fluorescence-activated cell sorting (FACS)^33,34^ (Fig. 1a, Extended Data Fig. 1a and Methods). The SVZ neurogenic niches of young (3–5 months old) and old (20–24 months old) GFAP-GFP mice were microdissected, and five cell populations from this region were freshly isolated by FACS—endothelial cells, astrocytes, qNSCs, activated NSCs (aNSCs) and neural progenitor cells (NPCs; Fig. 1a and Extended Data Fig. 1a). FACS markers generally did not change with age in isolated quiescent and activated NSCs (Extended Data Fig. 1a–d), suggesting that young and old cells are largely similar in terms of cell identity, consistent with our previous findings^34^.

**Extended Data Fig. 2:**
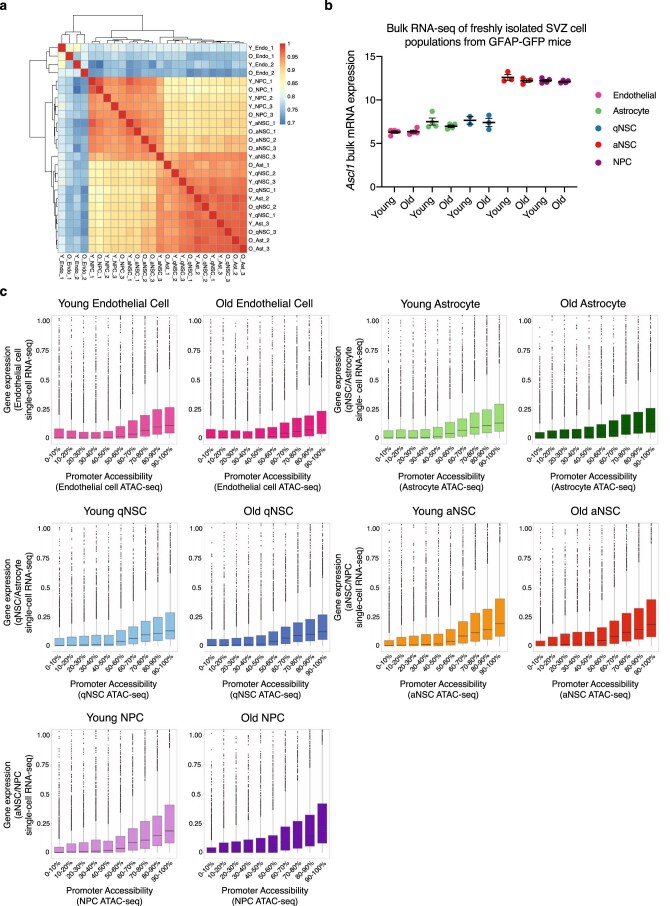
Chromatin accessibility clustering and correlation with gene expression. **a**, Hierarchical clustering using Pearson’s correlation of raw chromatin accessibility libraries (un-normalized) of all 5 cell types freshly isolated from the SVZ of young and old animals. Biological replicates are noted as numbers following age and cell type. **b**, mRNA expression of *Ascl1* in all 5 SVZ cell types in young and old conditions from published bulk RNA-seq dataset (see Methods). Each dot represents the VST-normalized mRNA expression value from a single RNA-seq library. For endothelial cells, astrocytes, and NPCs 4 libraries were made per condition using *n* = 8 young male and *n* = 8 young female GFAP-GFP mice (pairs of male and female mice were pooled), and *n* = 8 old male and *n* = 8 old female GFAP-GFP (pairs of male and female mice were pooled). For qNSCs and aNSCs, 3 libraries were made per condition using *n* = 6 young male and *n* = 6 young female GFAP-GFP mice (pairs of male and female mice were pooled), and *n* = 6 old male and *n* = 6 old female GFAP-GFP (pairs of male and female mice were pooled), mice except for the young qNSC condition which consisted of 2 libraries. Data are mean ± SEM. **c**, Decile plot of correlation between promoter accessibility levels (from ATAC-seq data) and mean gene expression values (from single cell RNA-seq data, see Methods). Promoters were binned into deciles based on accessibility level and boxplots were generated to correlate promoter bins with average gene expression for young and old endothelial cells, astrocytes, qNSCs, aNSCs, and NPCs. Each dot represents the gene expression level of an individual promoter binned based on chromatin accessibility signal. Y-axis constrained between 0 and 1 to facilitate boxplot visualization. Boxplot indicates median, and lower and upper quartiles. Minimum and maximum within 1.5× the interquartile range (whiskers) indicated. Dots outside whiskers represent outliers. *n* = 6 young male and *n* = 6 female GFAP-GFP mice (pairs of male and female mice were pooled), and *n* = 9 old male and *n* = 9 old female GFAP-GFP mice (pairs of male and female mice were pooled), combined over 3 independent experiments.

To assess chromatin accessibility genome-wide on these rare cell populations (~100-1000 cells per individual), we used the assay for transposase-accessible chromatin using sequencing (ATAC-seq^35–39^; Fig. 1a and Methods). ATAC-seq libraries across all conditions exhibited stereotypical 147-base-pair (bp) nucleosome periodicity (Extended Data Fig. 1e) and strong enrichment of accessibility around transcription start sites (TSSs; Extended Data Fig. 1f). Principal component analysis (PCA; Fig. 1b) and hierarchical clustering (Extended Data Fig. 2a) on accessible chromatin peaks separated endothelial cells from other brain cells, and quiescent cells (qNSCs and astrocytes) from activated ones (aNSCs and NPCs). The locus for *Ascl1* (encoding achaete-schute family bHLH transcription factor 1), a neural lineage gene involved in NSC activation^40^, showed accessible chromatin peaks in all neural cell types (but not in endothelial cells) and more accessible peaks in aNSCs and NPCs compared to qNSCs and astrocytes (Fig. 1c), consistent with *Ascl1* bulk mRNA expression^34^ (Extended Data Fig. 2b). Additionally, chromatin accessibility at genome-wide promoters positively correlated with gene expression from single-cell RNA-sequencing (RNA-seq) data^22^ (Extended Data Fig. 2c). These genome-wide chromatin accessibility datasets represent a useful resource for studying age-related chromatin changes in five different cell types freshly isolated from the SVZ neurogenic niche.

**Extended Data Fig. 3:**
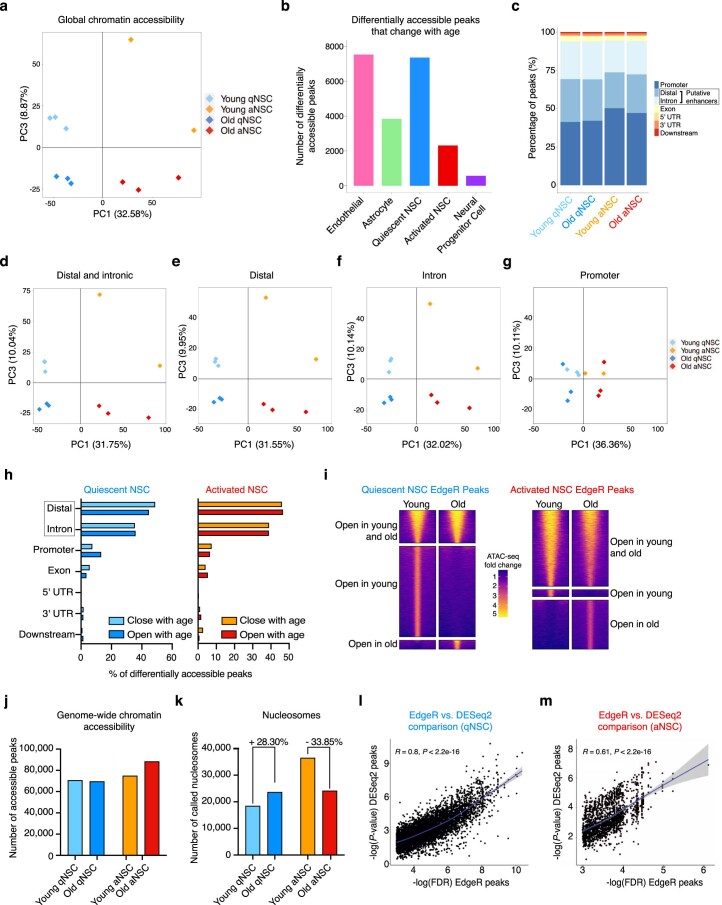
Chromatin accessibility in quiescent and activated NSCs during aging. **a**, PCA on genome-wide chromatin peaks defining qNSCs and aNSCs freshly isolated from the young and old SVZ where each dot represents a single ATAC-seq library (PC1 vs. PC3). PCA was generated using the variance stabilizing transformation (VST)-normalized NSC consensus count matrix. **b**, Barplot denoting the number of differentially accessible peaks that change with age (open and close) in freshly isolated endothelial cells, astrocytes, quiescent NSCs, activated NSCs, and neural progenitor cells. **c**, Stacked barchart representing the genomic distribution of open chromatin peaks for qNSCs and aNSCs in young and old conditions based on their pooled peaksets. ATAC-seq peaks were annotated with ChIPSeeker. Grey box indicates distal and intronic peaks that likely contain putative enhancers. **d-g**, PCA on distal and intronic chromatin peaks **(d)**, exclusively distal **I**, exclusively intronic **(f)**, and exclusively promoter **(g)** chromatin peaks for qNSCs and aNSCs freshly isolated from the SVZ of young and old mice where each dot represents a single ATAC-seq library (PC1 vs. PC3). PCA was generated using the variance stabilizing transformation (VST)-normalized NSC consensus count matrix. **h**, Frequency of differentially accessible ATAC-seq peaks that change with age in qNSCs and aNSCs associated with distal regions, introns, promoters, exons, 5’ UTRs, 3’ UTRs, and downstream regions. ATAC-seq peaks were annotated with ChIPSeeker. Box denotes differentially accessible distal and intronic peaks that likely contain putative enhancers. **I**, Raw signal pileup plots illustrating chromatin accessibility signal of quiescent and activated NSC chromatin peaks separated based on whether they are common to qNSCs and aNSCs (top row), open in young (middle row), or open in old (bottom) bottom as determined by the differential peak caller EdgeR. **j**, Barplot denoting the number of genome-wide chromatin peaks (non-differential) in freshly isolated young and old qNSCs and aNSCs. **k**, Number of nucleosome peaks called by NucleoATAC for freshly isolated young and old qNSC and aNSC chromatin landscapes. Nucleosome peaks were called using pooled, sub-sampled (to 30 million unique reads) ATAC-seq reads for each condition. **l,m**, Scatterplot illustrating correlation between significance levels of alternative differential peak calling methods: EdgeR FDR values vs DESeq2 *P*-values for peaks that are differentially open in young quiescent NSCs (called by EdgeR) (**l**) and open in old activated NSCs (**m**). Shaded region indicates 95% confidence interval. Regression and *P*-values were respectively calculated using geom_smooth() and state_cor() (two-tailed *t*-test) in R.

Chromatin accessibility allowed separation of quiescent and aNSCs by age (Fig. 1d and Extended Data Fig. 3a). In line with transcriptional studies^22,23,34^, more chromatin peaks change with age in qNSCs (7,354) than in aNSCs (2,311; false discovery rate (FDR) < 0.05; Extended Data Fig. 3b and Supplementary Table 2). As expected, chromatin peaks were largely located either at promoters or at intronic and distal regions, which are known to contain noncoding regulatory elements such as enhancers (Extended Data Fig. 3c). Chromatin peaks that overlap with marks of enhancers (H3K27ac and p300 binding^29^; Methods), as well as those at distal and intronic regions, were sufficient to separate NSCs based on age, whereas chromatin peaks at promoters alone were not (Fig. 1e and Extended Data Fig. 3d–g). Furthermore, chromatin peaks that dynamically change with age in qNSCs and aNSCs were almost entirely distal or intronic (Extended Data Fig. 3h and Supplementary Table 2). Thus, noncoding regulatory elements may be particularly sensitive to changes during aging.

Surprisingly, aging had opposing effects on chromatin accessibility dynamics in qNSCs and aNSCs. While most dynamic chromatin peaks in qNSCs closed with age, most dynamic chromatin peaks in aNSCs opened with age (Fig. 1f and Extended Data Fig. 3i,l,m). Likewise, the genome-wide chromatin landscape of old qNSCs contained less accessible chromatin peaks and more nucleosomes than that of young qNSCs, while the opposite was true for aNSCs (Extended Data Fig. 3j,k). These data suggest that chromatin of qNSCs becomes more repressed with age, while that of aNSCs becomes more permissive.

**Extended Data Fig. 4:**
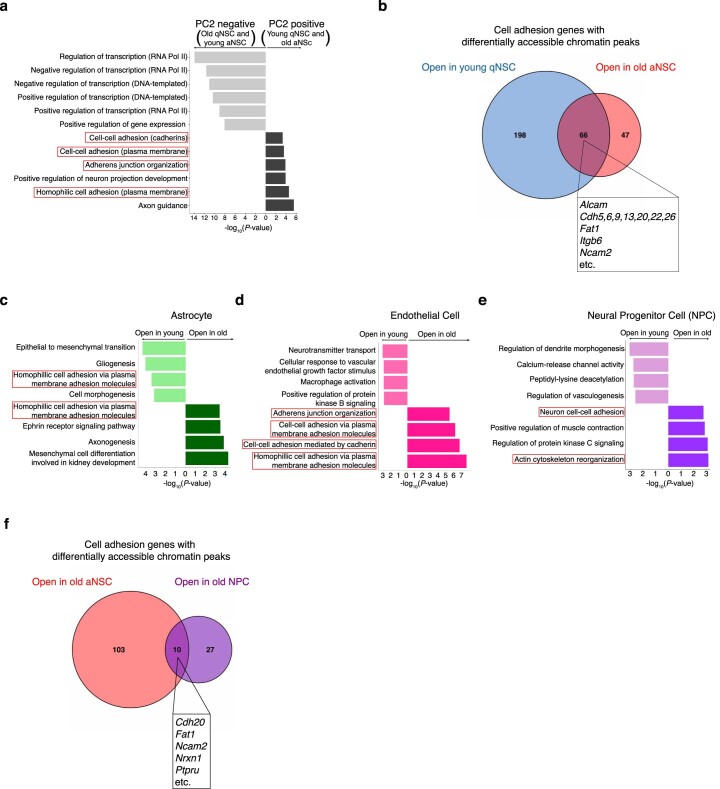
Chromatin accessibility analysis in 5 different cell types. **a**, Top 6 GO terms associated with the top 1000 ATAC-seq peak-associated genes driving PC2 in the positive (young qNSCs and old aNSCs) and negative (old qNSCs and young aNSCs) direction. Red boxes indicate GO terms associated with cell adhesion. *P*-values determined using a two-sided Fisher’s exact test. **b**, Venn diagram illustrating overlap between genes in ‘Cell Adhesion’ GO category (GO:0007155) with nearby chromatin peaks that are differentially open in young qNSCs (compared to old) and old aNSCs (compared to young). Box in overlap indicates select shared cell adhesion genes. **c-e**, Selected GO terms associated with differentially accessible ATAC-seq peaks (FDR < 0.05) that change with age in freshly isolated astrocytes **(c)**, endothelial cells **(d)**, and neural progenitor cells (NPCs) **(e)** generated by EnrichR and ranked by *P*-value (two-sided Fisher’s exact test). ATAC-seq peaks annotated with their nearest gene using ChIPSeeker. Red boxes indicate GO terms associated with adhesion and migration. **f**, Venn diagram illustrating overlap between genes in ‘Cell Adhesion’ GO category (GO:0007155) genes with nearby chromatin peaks that are differentially open in old aNSCs and old NPCs. Box in overlap indicates select shared cell adhesion genes.

Cell adhesion and migration is a defining hallmark of genes with opposing chromatin changes in both quiescent and activated NSCs during aging. Indeed, Gene Ontology (GO) biological pathway enrichment revealed that old qNSCs generally had decreased chromatin accessibility (open in young) at regulatory regions of genes involved in promoting cell adhesion and inhibiting cell migration (‘response to forskolin’, ‘cAMP-mediated signaling’, ‘negative regulation of cell migration’; Fig. 1g and Supplementary Table 3). Conversely, old aNSCs had increased chromatin accessibility (open in old) at regulatory regions of genes associated with cell adhesion, especially genes involved in cell–cell interactions (‘cell–cell adhesion (cadherins)’, ‘homophilic cell adhesion (plasma membrane)’, ‘adherens junction organization’) and some cell–matrix interactions (‘extracellular matrix assembly’) (Fig. 1h and Supplementary Table 3). Consistently, young qNSCs and old aNSCs were grouped together along the principal component 2 (PC2) axis in PCA, based largely on cell adhesion pathways (Extended Data Fig. 4a and Supplementary Tables 4 and 5).

Old qNSCs freshly isolated from the brain showed reduced chromatin accessibility at regulatory regions of genes involved in cell adhesion to the extracellular matrix (for example, integrins (*Itga3*, *Itgb6*)), cell–cell adhesion (for example, cadherins (*Cdh2*, *Cdh5)* and protocadherins (*Pcdh9*); Fig. 1i), and negative regulation of cell migration (for example, *Jag1*, *Nav3*; Fig. 1j). Conversely, old aNSCs exhibited increased chromatin accessibility at regulatory regions of genes implicated in cell adhesion to the extracellular matrix (for example, integrins (*Itga2, Itga3*)), cell–cell adhesion (for example, cadherins (*Cdh4, Cdh13*); Fig. 1i) and negative regulation of cell migration (for example, *Jag1*, *Nav3*; Fig. 1j and Supplementary Tables 2 and 3). A number of cell adhesion genes enriched in young qNSCs were shared with those enriched in old aNSCs (Extended Data Fig. 4b). Thus, chromatin accessibility dynamics suggest that quiescent and activated NSCs exhibit opposing changes at cell adhesion and migration genes with age.

Other cell types in the niche showed changes in chromatin accessibility with age, including at genes involved in adhesion pathways (Extended Data Fig. 4c–e and Supplementary Table 3). For example, endothelial cells and NPCs had increased chromatin accessibility at adhesion pathways with age (Extended Data Fig. 4d,e and Supplementary Table 3). Interestingly, a number of cell adhesion genes with age-related chromatin changes in old aNSCs and old NPCs were shared, suggesting that some chromatin changes may be long-lasting and preserved in downstream progeny (Extended Data Fig. 4f).

Thus, aging has opposing effects on the global chromatin landscape of quiescent and activated NSCs (and NPC progeny), including opposing changes to chromatin accessibility in regulatory regions involved in cell adhesion and migration.

## Opposing gene expression changes in quiescent and activated neural stem cells with age

**Fig. 2:**
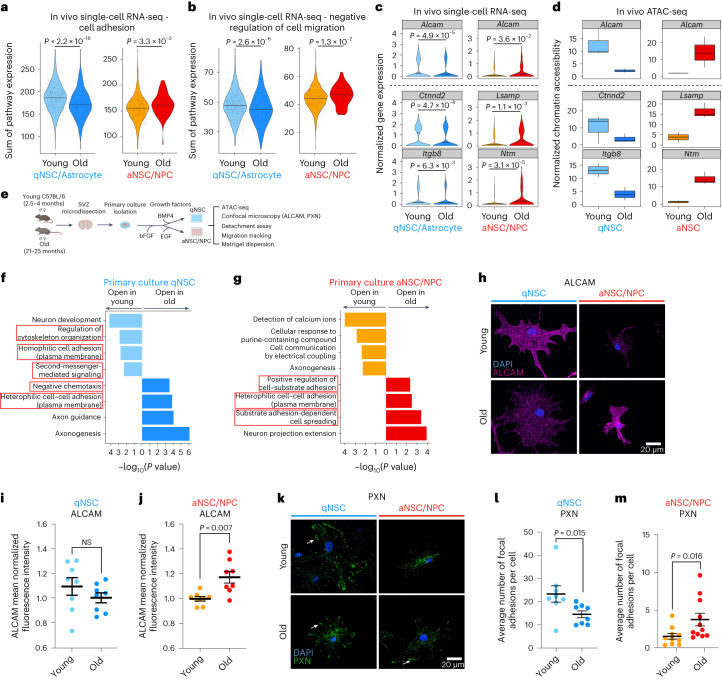
Opposing chromatin changes in quiescent and activated NSCs during aging are associated with gene expression changes. **a**,**b**, Violin plots of cumulative expression profiles of genes within the ‘cell adhesion’ (**a**) and ‘negative regulation of cell migration’ (**b**) GO categories for young and old qNSCs/astrocytes and aNSCs/NPCs from single-cell RNA-seq data. Each dot represents the cumulative expression of genes within the GO category in a single cell. **c**, Violin plots of select cell adhesion gene expression profiles for young and old qNSCs/astrocytes and aNSCs/NPCs from single-cell RNA-seq data. **d**, Box plots of TMM-normalized chromatin accessibility changes in differentially accessible peaks near cell adhesion genes from **c** for young and old qNSCs and aNSCs. Box plot displays the median and lower and upper quartile values. Minimum and maximum values within 1.5 times the interquartile range (whiskers) are indicated. *n* = 6 young male and *n* = 6 young female GFAP-GFP mice, and *n* = 9 old male and *n* = 9 old female GFAP-GFP mice (pairs of male and female mice were pooled). **c**,**d**, Genes above the dashed line are examples of genes that are shared between qNSCs/astrocytes and aNSCs/NPCs and genes below the dashed line are examples of genes that are not shared. **e**, Design for primary cultures of qNSCs and aNSCs/NPCs. Created with BioRender.com. **f**,**g**, Selected GO terms for genes associated with differentially accessible ATAC-seq peaks (FDR < 0.05) that change with age in primary cultures of qNSCs (**f**) and aNSCs/NPCs (**g**) generated by EnrichR and ranked by *P* value (two-sided Fisher’s exact test). Red boxes indicate GO terms associated with cell adhesion and migration. **h**, Representative images of immunofluorescence staining for ALCAM in young and old qNSCs and aNSCs/NPCs. Purple, ALCAM. Blue, DAPI. Scale bar, 20 μm. **i**,**j**, Quantification of ALCAM mean normalized fluorescence intensity of young and old qNSCs (**i**) and aNSCs/NPCs (**j**). Each dot represents the mean fluorescence intensity of 30 fields (each containing 1–3 cells) in a primary culture derived from an individual mouse, normalized by experiment and cell size. NS, not significant. **h**–**j,** *n* = 8 young and *n* = 8 old male mice. **k**, Representative images of immunofluorescence staining for PXN of young and old qNSCs and aNSCs/NPCs. Green, PXN. Blue, DAPI. Arrow indicates localization of PXN to peripheral focal adhesions. Scale bar, 20 μm. **l**,**m**, Quantification of PXN immunostaining of young and old qNSCs (**l**) and aNSCs/NPCs (**m**). Each dot represents the average number of focal adhesions per cell (28–32 cells per dot) in a primary culture derived from an individual mouse. In **k**–**m**, *n* = 8 young and *n* = 8 old male mice (qNSCs), *n* = 10 young and *n* = 11 old male mice (aNSCs/NPCs). In **i**, **j**, **l** and **m**, data are the mean ± s.e.m. Data were combined over three (**d**, **j** and **m**) or two (**i** and **l**) independent experiments. All statistical comparisons were made using a two-tailed Mann–Whitney test unless otherwise stated. Source data

**Extended Data Fig. 5:**
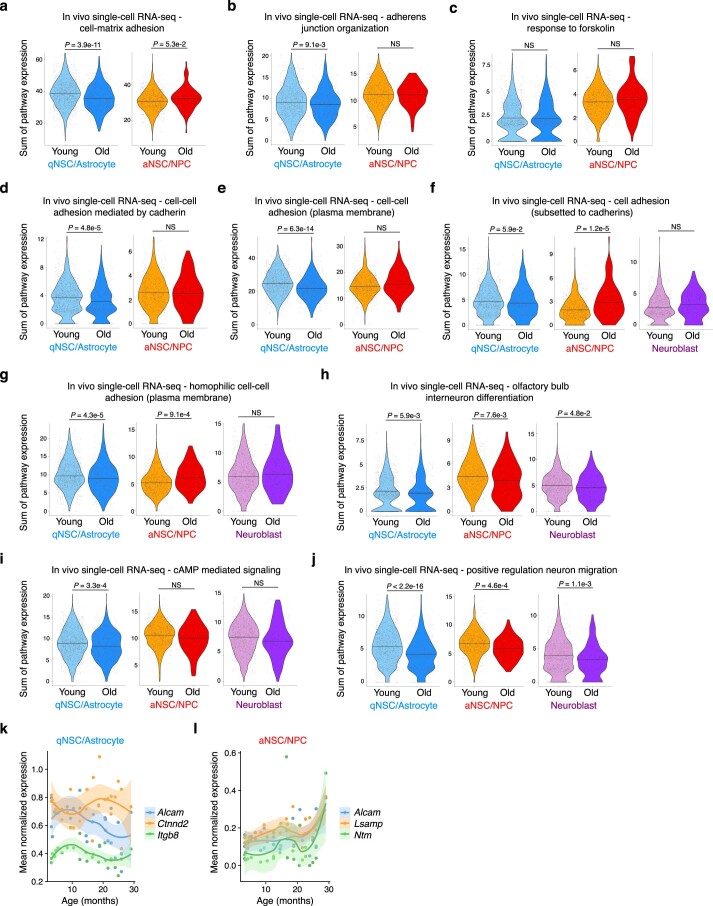
Gene expression of cell adhesion and migration pathways in NSC lineage during aging by single cell RNA-seq. **a-e**, Violin plots of the distribution of single cell cumulative expression profiles of genes within the ‘Cell-matrix adhesion’ GO category (GO:0007160) **(a)**, ‘Adherens junction organization’ GO category (GO:0034332) **(b)**, ‘Response to forskolin’ GO Category (GO:1904322) **(c)**, ‘Cell-cell adhesion mediated by cadherin’ GO Category (GO:0044331) **(d)**, and ‘Cell-cell adhesion (plasma membrane)’ GO Category (GO:0098742) **I** for young and old qNSCs/Astrocytes (left) and aNSCs/NPCs (right) from single cell RNA-seq data (see Methods). Each overlaid dot represents the cumulative expression of genes within the GO category in a single cell. **f-j**, Violin plots of the distribution of single cell cumulative expression profiles of genes within the ‘Cell adhesion’ GO Category (GO:0007155) restricted to cadherins **(f)**, ‘Homophilic cell-cell adhesion (plasma membrane)’ GO Category (GO:0007156) **(g)**, ‘Olfactory bulb interneuron differentiation’ GO Category (GO:0021889) **(h)**, ‘cAMP mediated signaling’ GO Category (GO:0019933) **(i)**, and ‘Positive regulation neuron migration’ GO Category (GO:2001224) **(j)** for young and old qNSC/Astrocyte (left), aNSC/NPC (middle), and neuroblasts (right) from single cell RNA-seq data (see Methods). Each overlaid dot represents the cumulative expression of genes within the GO category in a single cell. **k**, Gene expression trajectories as a function of age of select cell adhesion genes (*Alcam, Ctnnd2, Itgb8*) in qNSCs/Astrocytes from single cell RNA-seq dataset (see Methods). Shaded region represents 95% confidence interval. **l**, Gene expression trajectories as a function of age of select cell adhesion genes (*Alcam, Lsamp, Ntm*) in aNSCs/NPCs from single cell RNA-seq dataset (see Methods). Shaded region represents 95% confidence interval. All statistical comparisons made using a two-tailed Mann-Whitney test.

We next asked if the opposing changes to chromatin in quiescent and activated NSCs during aging are associated with expression changes in cell adhesion and migration genes. Analysis of available single-cell RNA-seq datasets from neurogenic niches of young and old mice^22,41^ revealed that old quiescent and activated NSCs showed opposing expression changes in gene signatures involved in cell adhesion, negative regulation of migration, cell–cell adhesion and cell–matrix adhesion (Fig. 2a,b and Extended Data Fig. 5), consistent with predictions from chromatin changes (with the exception of ‘cell–cell adhesion (plasma membrane)’, which showed increased chromatin accessibility in old qNSCs but decreased gene expression in old qNSCs (Fig. 1g and Extended Data Fig. 5e)). Some of these gene expression changes were also preserved in neuroblasts, suggesting that they can persist in downstream progeny (Extended Data Fig. 5h,j).

Old qNSCs/astrocytes showed downregulation of genes involved in adhesion to the extracellular matrix (for example, *Itgb8*) or cell–cell adhesion (for example, *Alcam*, *Ctnnd2*; Fig. 2c and Extended Data Fig. 5k). Conversely, old aNSCs/NPCs showed upregulation of genes involved in cell–cell adhesion (for example, *Alcam*, *Lsamp*, *Ntm*; Fig. 2c and Extended Data Fig. 5l), consistent with chromatin accessibility changes at these genetic loci (Fig. 2d). Thus, changes in gene expression of cell adhesion and migration pathways by single-cell RNA-seq generally corroborate opposing changes in chromatin states in quiescent and activated NSCs. A few genes did not exhibit a correlation between chromatin and expression (for example, *Cadm2* and *Itgb1* for qNSCs and *Ccdc80* and *Epdr1* for aNSCs), and in these cases age-dependent chromatin changes may be poising genes for future expression in downstream progeny (for example, neuroblasts).

**Extended Data Fig. 6:**
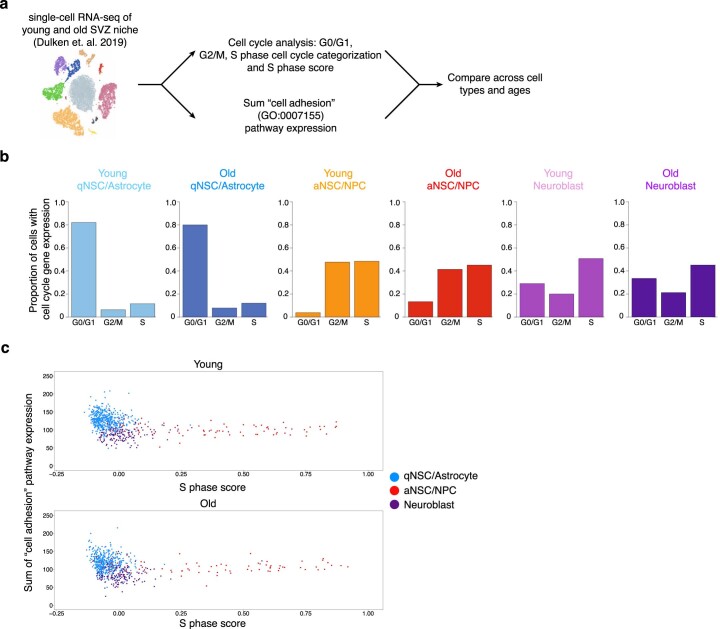
Analysis of cell cycle and cell migration signatures in NSCs during aging by single cell RNA-seq. **a**, Single cell RNA-seq data was used to assess cell cycle gene expression and Cell Adhesion (GO:0007155) pathway expression for young and old qNSCs/astrocytes, aNSCs/NPCs, and neuroblasts (see Methods). Created with BioRender.com. **b**, Barplots illustrating the proportion of young and old qNSCs/astrocytes, aNSCs/NPCs, and neuroblasts in the GO/G1, G2/M, or S phase of the cell cycle based on gene expression signatures from single cell RNA-seq data (see Methods). **c**, Scatterplot of qNSCs/astrocytes, aNSCs/NPCs, and neuroblasts from young (top) and old (bottom) single cell RNA-seq data (see Methods) illustrating the relationship between Seurat’s ‘S phase score’ and the cumulative single cell expression levels of the genes in the ‘Cell Adhesion’ GO category (GO:0007155). Young cells were downsampled to visualize the same number of young and old cells per cell type (480 qNSCs/astrocytes, 82 aNSCs/NPCs, and 146 neuroblasts).

We used single-cell RNA-seq data to examine whether changes in adhesion and migration genes occurred at the level of individual cells or were due to changes in NSC subpopulations. For most cell adhesion and migration pathways, single-cell gene expression data were not bimodal (Fig. 2a,b and Extended Data Fig. 5), suggesting that changes occur in individual cells. In addition, while cell cycle analysis of single-cell RNA-seq data indicated a small shift toward a subpopulation of more ‘quiescent-like’ cells in old activated NSCs/NPCs (Extended Data Fig. 6a,b), not all age-dependent adhesion expression changes occurred in this subpopulation (Extended Data Fig. 6c). Thus, age-dependent expression changes occur mostly in individual cells, although subpopulation differences in old aNSCs may also contribute.

**Extended Data Fig. 7:**
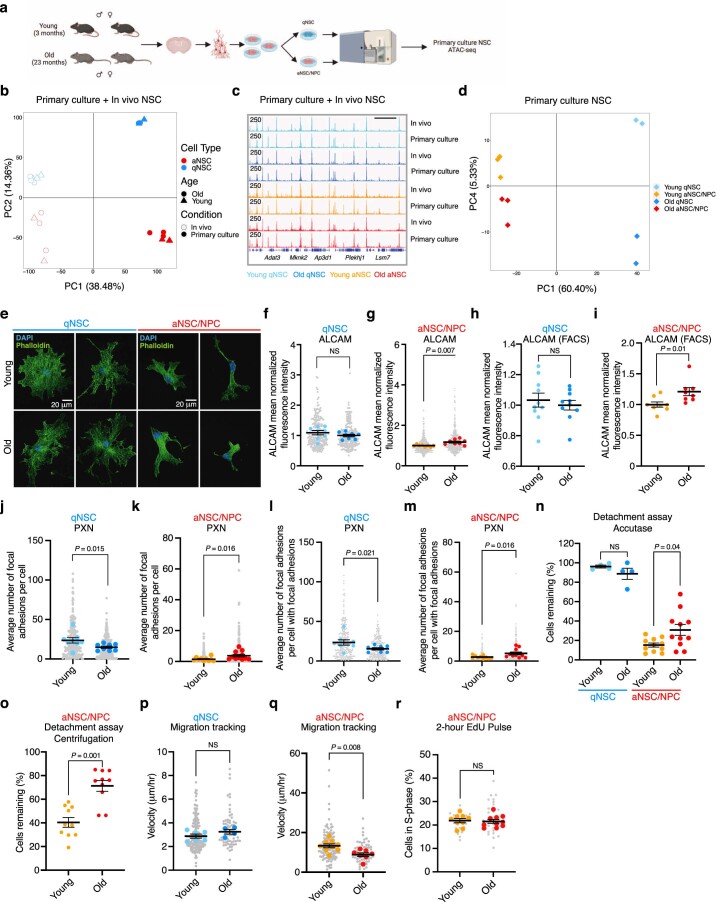
Primary cultures of qNSCs and aNSCs/NPCs for ATAC-seq, immunostaining, and adhesion and migration assays. **a**, Experimental design for the generation of ATAC-seq libraries from quiescent and activated NSCs cultured from young and old mixed-sex C57BL/6 mice. Created with BioRender.com. **b**, PCA on chromatin peaks defining cultured and in vivo qNSC and aNSC landscapes where each dot represents a single ATAC-seq library (PC1 vs. PC2). PCA was generated using the variance stabilizing transformation (VST)-normalized global consensus count matrix of all cultured and in vivo NSC peaks. **c**, Genome browser (IGV) view of chromatin accessibility signal tracks on chromosome 10 of freshly isolated and cultured qNSC and aNSC from the young and old SVZ. *Adat3*, Adenosine Deaminase TRNA Specific 3. *Mknk2*, MAPK Interacting Serine/Threonine Kinase 2. *Ap3d1*, Adaptor Related Protein Complex 3 Subunit Delta 1. *Plekhj1*, Pleckstrin Homology Domain Containing J1. *Lsm7*, LSM7 Homology U6 Small Nuclear RNA and mRNA Degradation Associated. Scale bar, 50 kb. **d**, PCA on chromatin peaks defining qNSC and aNSC/NPC cultured from young and old SVZs where each dot represents a single ATAC-seq library (PC1 vs. PC4). PCA was generated using the variance stabilizing transformation (VST)-normalized global consensus count matrix. **e**, Representative immunofluorescent images of young and old qNSC and aNSC/NPC highlighting heterogeneity in cell size and shape. Green, phalloidin (F-actin). Blue, DAPI. Scale bar, 20 μm. **f, g**, ALCAM fluorescence intensity of young and old qNSC **(f)** and aNSC/NPC **(g)**. Each grey dot represents mean normalized fluorescent intensity per cell. Each colored dot represents mean normalized fluorescence intensity of 30 fields (each containing 1-3 cells) in a primary culture derived from an individual mouse, normalized by experiment and cell size. *n* = 8 young male mice, and *n* = 8 old male mice, combined over 2 (qNSC) or 3 (aNSC/NPC) independent experiments. **h,I**, Normalized fluorescence intensity of ALCAM, as assessed by FACS, in young and old qNSCs **(h)** and aNSCs/NPCs (**i**). Each colored dot represents mean normalized fluorescence intensity of ~10,000 live cells from an individual mouse, normalized by experiment. *n* = 10 young male mice, and *n* = 10 old male mice (qNSCs), *n* = 8 young male mice, and *n* = 8 old male mice (aNSCs/NPCs), combined over 2 independent experiments. **j,k**, Quantification of paxillin (PXN) immunostaining of young and old qNSCs **(j)** and aNSCs/NPCs **(k)**. Each grey dot represents number of focal adhesions per cell. Each colored dot represents average number of focal adhesions per cell (28-32 cells per dot) in a primary culture derived from an individual mouse. *n* = 8 young male mice, and *n* = 8 old male mice (qNSCs), *n* = 10 young male mice, and *n* = 11 old male mice (aNSCs/NPCs), combined over 2 (qNSCs) or 3 (aNSCs/NPCs) independent experiments. **l,m**, Quantification of subpopulation of cells with at least one active focal adhesion as assessed with paxillin (PXN) immunostaining of young and old qNSCs **(l)** and aNSCs/NPCs **(m)**. Each grey dot represents number of focal adhesions per cell. Each colored dot represents average number of focal adhesions per cell of cells that have at least once focal adhesion (4-23 cells per dot) from an individual mouse. *n* = 8 young male mice, and *n* = 8 old male mice (qNSCs), *n* = 10 young male mice and *n* = 11 old male mice (aNSCs/NPCs), combined over 2 (qNSCs) or 3 (aNSCs/NPCs) independent experiments. Same experiment as in (j) and (k). **n**, Quantification of percent cells remaining of young and old qNSCs and young and old aNSCs/NPCs after a 5 minute incubation with Accutase and PBS wash. Each dot represents average percent cells remaining after Accutase treatment of 2-4 technical replicates (wells) per primary culture derived from an individual mouse. *n* = 4 young male mice and *n* = 4 old male mice (qNSCs), *n* = 11 young male mice and *n* = 11 old male mice (aNSCs/NPCs) from one independent experiment (qNSCs) or combined over 6 independent experiments (aNSCs/NPCs). Note that the aNSC/NPC data are the same as in Fig. 3c. **o**, Force-based assay to assess aNSC/NPC adhesion. Quantification of percent area remaining of young and old aNSCs/NPCs after cells were detached by centrifugal force (300 g for 4 minutes). Each dot represents average percent area remaining after centrifugation of 2-3 technical replicates (wells) per primary culture derived from an individual mouse. *n* = 10 young male mice, and *n* = 10 old male mice, combined over 2 independent experiments. **p**, Migration speed of young and old qNSCs. Each grey dot represents the average velocity of a single cell over a 20-hour period. Each dot represents the average velocity over a 20-hour period of 5-42 cells in a primary culture derived from an individual mouse. *n* = 6 young male mice and *n* = 4 old male mice, combined over 2 independent experiments. **q**, Migration speed of young old aNSCs/NPCs. Each grey dot represents the average velocity of a single cell over a 20-hour period. Each colored dot represents the average velocity per mouse over a 20-hour period of 6-28 cells. *n* = 9 young male mice and *n* = 7 old male mice, combined over 3 independent experiments. **r**, Percent cells that are EdU-positive (S-phase) after a 2-hour pulse for young and old aNSCs/NPCs. Each grey dot represents percent of cells in given field that are EdU-positive. Each colored dot represents average percent cells EdU-positive for an individual mouse (average of 5-7 fields containing at least 100 cells each). *n* = 9 young male mice, and *n* = 10 old male mice, combined over 3 independent experiments. All data are mean ± SEM. All statistical comparisons made using a two-tailed Mann-Whitney test comparing sample means. Data from independent experiments are in Source Data. Ages of animals used provided in Source Data. Source data

To experimentally test the prediction that adhesion and migration properties of NSCs change during aging, we used a culture system for qNSCs and proliferative NSCs (a mix of aNSCs/NPCs)^29,34,42–44^ (Fig. 2e). In this culture system, cells are directly isolated from the SVZ neurogenic niches of young and old mice, and cell identity is maintained in culture with specific growth factors (Fig. 2e and Methods). We verified that the chromatin landscape of cultured NSCs was similar to that of freshly isolated NSCs (Extended Data Fig. 7a–d and Supplementary Tables 2,3), notably in age-related changes to cell adhesion and migration pathways (Fig. 2f,g and Supplementary Table 3).

To examine specific adhesion proteins or pathways identified by changes in chromatin and gene expression, we performed immunostaining on young and old primary cultures of qNSCs and aNSCs/NPCs (Fig. 2e and Extended Data Fig. 7e). We first stained for ALCAM—a transmembrane glycoprotein directly involved in cell–cell adhesion^45,46^ and indirectly implicated in cell–matrix adhesion^47^—whose gene exhibits opposing chromatin and gene expression changes in quiescent and activated NSCs with age (Fig. 2c,d). ALCAM immunostaining quantification revealed that old qNSCs exhibited a modest (non-significant) decrease in ALCAM protein levels (Fig. 2h,i and Extended Data Fig. 7f), while old aNSCs/NPCs showed a significant increase in ALCAM protein levels compared to their young counterparts (Fig. 2h,j and Extended Data Fig. 7g). These age-dependent changes were also observed using FACS (Extended Data Fig. 7h,i). We next stained for paxillin (PXN)—a marker of focal adhesions and cell–matrix adhesion^48,49^—given that integrin genes, which regulate focal adhesions, exhibit opposing age-dependent chromatin changes in quiescent and activated NSCs with age and some of them also show gene expression changes (Figs. 1i,j and 2c,d). PXN immunostaining quantification showed that old qNSCs had fewer focal adhesions than their young counterparts, whereas old aNSCs/NPCs had more focal adhesions than young counterparts (Fig. 2k–m and Extended Data Fig. 7j,k). These opposing changes in PXN staining were also observed within the subpopulation of cells containing at least one focal adhesion (Extended Data Fig. 7l,m), suggesting that aging could induce changes at the individual cell level. These immunostaining data corroborate the opposing changes in adhesion pathways in quiescent and activated NSCs with age predicted by chromatin and transcriptional data and indicate that at least some of the age-related changes in adhesion molecules occur at the level of individual cells.

## Opposing adhesion changes in quiescent and activated neural stem cells with age

**Fig. 3:**
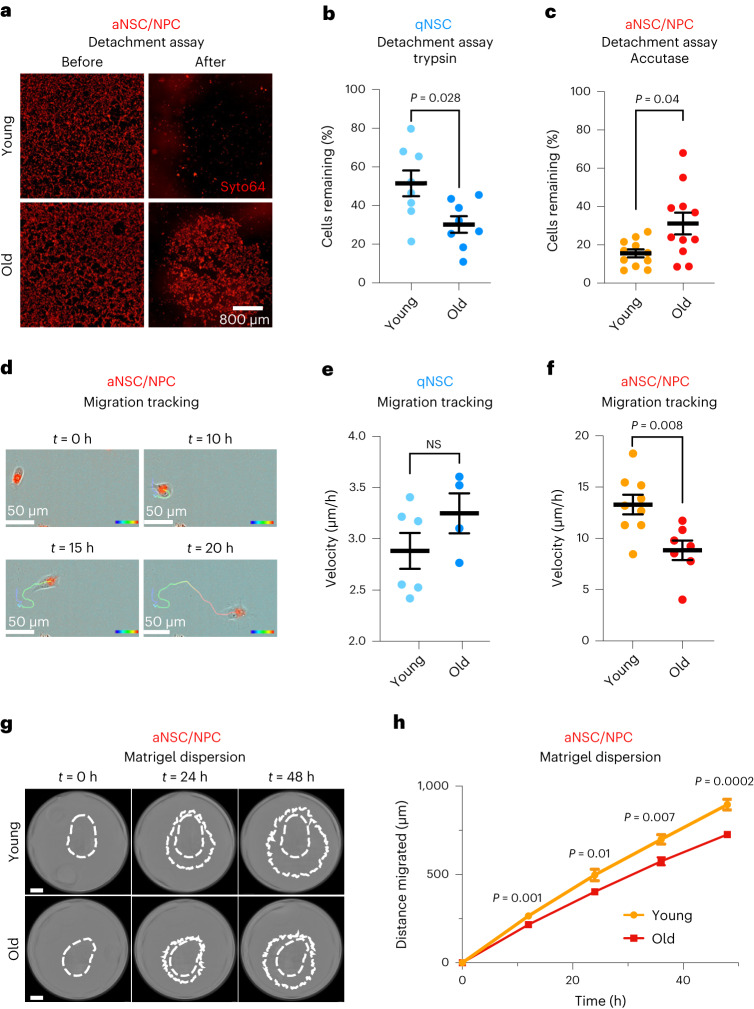
Opposing chromatin changes in quiescent and activated neural stem cells during aging are associated with functional defects in cell adhesion and migration. **a**, Representative images of live young and old aNSCs/NPCs stained with Syto64 taken before and Accutase treatment. Scale bar, 800 μm. **b**, Percentage of cells remaining of young and old qNSCs after trypsin treatment. Each dot represents the average percentage of cells remaining after trypsin treatment of 2–4 technical replicates per primary culture derived from an individual mouse. *n* = 8 young and *n* = 8 old male mice. **c**, Percentage of cells remaining of young and old aNSCs/NPCs after Accutase treatment. Each dot represents the average percentage of cells remaining after Accutase treatment of 2–4 technical replicates per primary culture derived from an individual mouse. **a**,**c**, *n* = 11 young and *n* = 11 old male mice. **d**, Representative images of the migration path of a young aNSC or NPC. Color bar represents the passage of time from 0 h (blue) to 20 h (red). Scale bars, 50 μm. **e**, Migration speed of young and old qNSCs. *n* = 6 young and *n* = 4 old male mice. **f**, Migration speed of young and old aNSCs/NPCs. **e**,**f**, Each dot represents the average velocity over a 20-h period of 5–42 cells in a primary culture derived from an individual mouse. **d**,**f**, *n* = 9 young and *n* = 7 old male mice. **g**, Representative images of young and old aNSC/NPC dispersion through Matrigel. The outer dashed line represents the outermost extent of invasion and the inner dashed line represents the initial extent of the cells after plating (*t* = 0 h). Scale bar, 800 μm. **h**, Migration distance of young and old aNSC/NPC dispersion through Matrigel over 48 h. At each time point, distance was averaged over 1–4 technical replicates from a primary culture derived from an individual mouse. **g**,**h**, *n* = 7 young and *n* = 10 old male mice. All data are the mean ± s.e.m. Data were combined over six (**a** and **c**), two (**b**, **e**, **g** and **h**) or three (**d** and **f**) independent experiments. All statistical comparisons were made using a two-tailed Mann–Whitney. Source data

We functionally assessed if aging impacts cell adhesion in quiescent and activated NSCs in cell culture. To probe cell adhesion, we adapted a detachment assay used in other cell types^50,51^ for NSCs. In this assay, cultured NSCs are plated as a monolayer and imaged before and after incubation with specific enzymes (trypsin or Accutase). These enzymes detach cells by cleaving cell adhesion proteins (although they also cleave extracellular matrix proteins, which could be differentially deposited by cells). We used trypsin for qNSCs, which are very adhesive cells, and Accutase for aNSCs/NPCs, which are less adhesive cells (Extended Data Fig. 7n). Old qNSCs detached more easily than their young counterparts, whereas old aNSCs/NPCs detached less easily than their young counterparts (Fig. 3a–c). In a complementary detachment assay^52,53^ that uses mechanical force to detach cells, old aNSCs/NPCs also detached less easily than their young counterparts (Extended Data Fig. 7o). These data suggest that qNSCs become less adhesive with age, whereas aNSCs become more adhesive with age.

**Extended Data Fig. 8:**
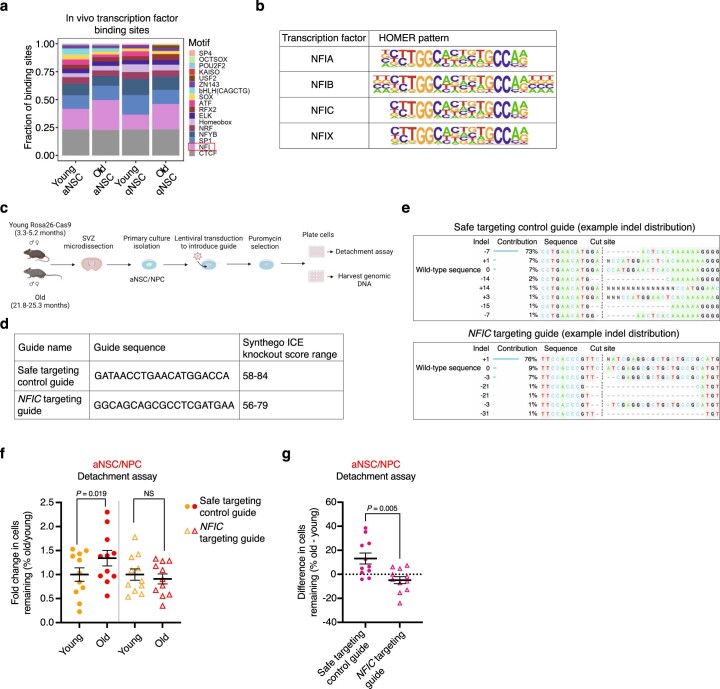
NFI family of transcription factors during aging in activated NSCs. **a**, Distribution of transcription factor binding sites in accessible chromatin regions from freshly isolated young and old aNSCs and qNSCs identified using a deep learning model (see Methods). **b**, NFI family transcription factor binding site motifs for each NFI isoform from HOMER. **c**, Experimental design for knocking out *NFIC* in primary cultures of young and old aNSCs/NPCs. Created with BioRender.com. **d**, Guide sequences used for safe targeting control guide which targets a safe harbor locus (gene that can be modified or disrupted with CRISPR-Cas9 without any adverse effect on the cell) and *NFIC* targeting guide from Bassik Lab mouse CRISPR Knockout library (see Methods) as well as Synthego ICE knockout score range achieved with each guide. With this tool, a score of 60 indicates 60% of the population sequenced has a frameshift mutation or deletion greater than 21 base pairs. **e**, Sample traces for safe targeting control guide and *NFIC* targeting guide from Synthego ICE analysis tool indicating indel distribution and cut site. **f**, Detachment assay upon *NFIC* knockout in young and old aNSC/NPC using *NFIC* targeting guide. Quantification of percent of aNSCs/NPCs remaining after 3 minute Accutase incubation and PBS wash. Each data point is average of 2-3 technical replicates (wells) per primary culture derived from an individual mouse. Safe targeting control guide samples (solid dot) were normalized to average of all young safe targeting control samples. *NFIC* targeting guide samples (open triangle) were normalized to average of all young *NFIC* targeting guide samples. *P*-values calculated using a two-sided Wilcoxon Matched-Pairs Signed Ranks Test, since experiments were performed in a paired manner (paired young and old). **g**, Detachment assay upon *NFIC* knockout in young and old aNSCs/NPCs using *NFIC* targeting guide. Same experiment as in (f). Quantification of difference of percent of aNSCs/NPCs remaining (old – young) after 3 minute Accutase incubation and wash with 1x PBS for safe targeting control guide samples (solid dot) and *NFIC* targeting guide samples (open triangle). *P*-value was calculated using a two-tailed Mann-Whitney test. **f,g**, Data are mean ± SEM. *n* = 11 young male and female mice, and *n* = 11 old male and female mice, combined over 7 independent experiments. Data from independent experiments are in Source Data. Ages of animals used provided in Source Data. Source data

We next tested the functional importance of age-regulated changes in chromatin accessibility for cell adhesion in aNSCs. We focused on the NFI family of transcription factors because it regulates cell adhesion^29^ and is enriched in the accessible chromatin landscape of old aNSCs (Extended Data Fig. 8a,b). Interestingly, CRISPR–Cas9 knockout of the NFIC transcription factor family member blunted the difference in cell adhesion between young and old aNSCs, with aNSCs/NPCs lacking NFIC no longer exhibiting increased adhesion with age (Extended Data Fig. 8c–g). Hence, aging has opposing effects on the adhesive properties of quiescent and activated NSCs, and alterations in NFIC regulation during aging may contribute to increased adhesion in old aNSCs.

## Old activated neural stem cells exhibit defects in migration with age

To assess the migratory properties of NSCs, we performed continuous live-cell imaging of NSCs cultured from young and old brains over 20 h (Fig. 3d). Whereas old qNSCs were slightly more migratory than their young counterparts (non-significant; Fig. 3e and Extended Data Fig. 7p), old aNSCs/NPCs were significantly less migratory than their young counterparts (Fig. 3f and Extended Data Fig. 7q). We also quantified the dispersion of cultured aNSCs/NPCs through Matrigel, an assay that integrates migration as well as other factors (for example, proliferation). In this assay, old aNSCs/NPCs also exhibited impaired ability to disperse through extracellular matrix compared to their young counterparts (Fig. 3g,h). These dispersion differences are likely due to defects in migration (rather than proliferation) because they already manifest at early time points (12 h, before NSCs would have time to significantly expand) and there were no significant proliferation differences in cultured young and old aNSCs/NPCs (Extended Data Fig. 7r). These results suggest that aNSCs become less migratory during aging.

## Aging disrupts the location of quiescent and activated neural progenitor cells in vivo

In the SVZ neurogenic niche, qNSCs line the ventricles and can become activated (aNSCs) to give rise to NPCs and neuroblasts^1–4^. Neuroblasts then migrate long distances along the RMS to the OB to produce new neurons (neurogenesis)^3,12,54^. While NSC/NPC and neuroblast migration has been examined in older animals^55–59^, the adhesive and migratory properties of quiescent and activated NSCs (and their progenitors) have not been systematically studied in vivo during aging.

Location within the SVZ niche is important for NSC function^60,61^, and adhesion and migration defects in quiescent and activated NSCs during aging could manifest as changes to their niche location. We assessed the location of quiescent and activated NSCs with respect to the ventricle in the SVZ neurogenic niche during aging. To this end, we immunostained brain sections from young and old individuals using markers such as GFAP (NSCs and astrocytes), S100a6 (NSCs in the adult SVZ neurogenic niche^62^), Ki67 (proliferating cells) and, in some, cases DCX (neuroblasts).

**Fig. 4:**
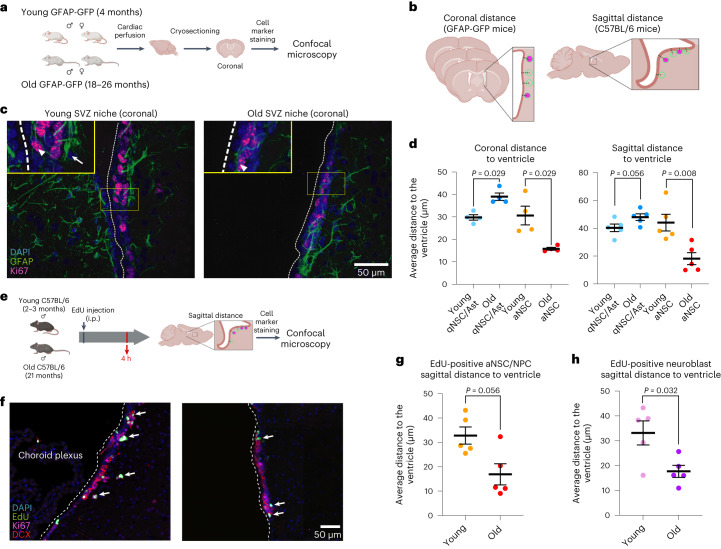
Age-dependent location defects of quiescent and activated neural stem cells and progeny in vivo. **a**, Design of immunofluorescence experiments for quantifying the location of qNSCs/astrocytes and aNSCs in vivo in coronal brain sections. **b**, Schematic depicting how distance of cells to the ventricle were quantified. **c**, Representative images of immunofluorescence staining of coronal SVZ sections from young and old GFAP-GFP mice. The yellow box denotes the inset containing a qNSC/niche astrocyte (arrow) and an aNSC (arrowhead). The ventricular lining is indicated by a dashed white line (see Extended Data Fig. 9a for demarcation of ventricle wall with vinculin). Green, GFAP (astrocyte/NSC); pink, Ki67 (proliferation); blue, DAPI. Scale bar, 50 μm. **d**, NSC distance to the ventricle for qNSCs and niche astrocytes (Ast) and aNSCs in serial coronal sections (left) of young and old SVZs from mixed-sex GFAP-GFP mice and sagittal sections (right) of young and old SVZs from male C57BL/6 mice. Each dot represents the mean distance from the ventricle per mouse. Serial coronal sections: *n* = 4 young and *n* = 4 old mixed-sex GFAP-GFP mice, combined over four independent experiments. Sagittal sections: *n* = 5 young and *n* = 5 old male C57BL/6 mice, combined over two independent experiments. **e**, Design of immunofluorescence experiments to assess the location of EdU-labeled aNSCs/NPCs and neuroblasts in the SVZ neurogenic niche in vivo. i.p., intraperitoneal. **f**, Representative images of immunofluorescence staining of sagittal SVZ sections of young and old male C57BL/6 mice 4 h after EdU injection. Green, EdU; pink, Ki67 (aNSC/NPC/neuroblast); red, DCX (neuroblast); blue, DAPI. The dashed white line indicates the ventricle wall and arrows indicate EdU^+^ cells. Scale bar, 50 μm. **g**,**h**, Distance to the ventricle for EdU^+^ aNSCs/NPCs (**g**) and EdU^+^ neuroblasts (**h**) in sagittal sections of young and old SVZs 4 h after EdU injection. Each dot represents the mean distance from the ventricle per mouse. **f**–**h**, *n* = 5 young and *n* = 5 old male mice, combined over two experiments. All data are the mean ± s.e.m. All statistical comparisons were made using a two-tailed Mann–Whitney test. Figures in **a**, **b** and **e** were created with BioRender.com. Source data

**Extended Data Fig. 9:**
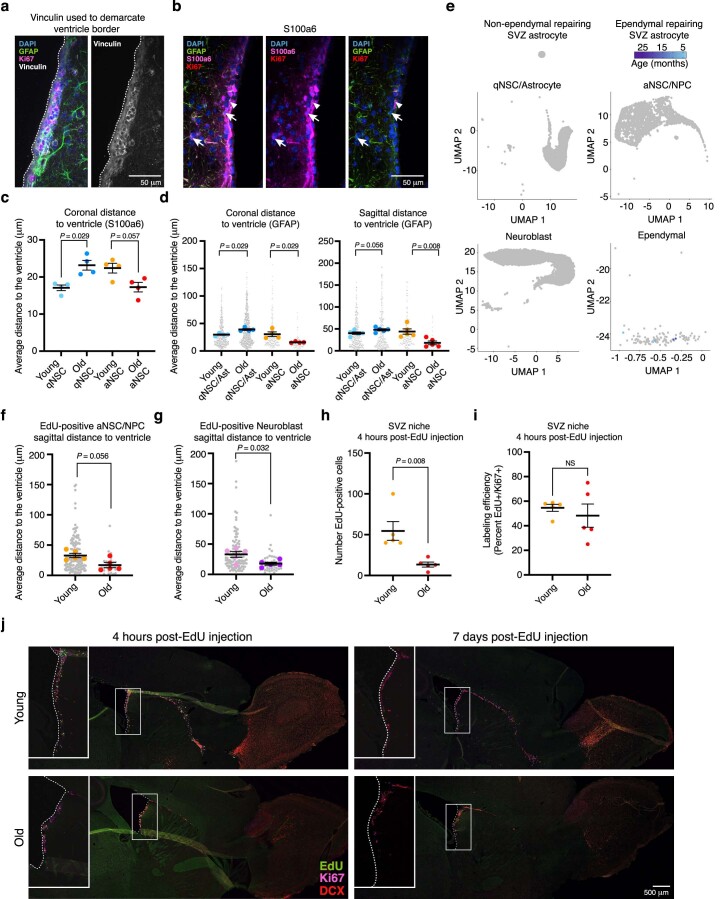
In vivo location of EdU-labelled cells in the SVZ neurogenic niche. **a**, Representative image of vinculin immunofluorescence staining of old coronal section from GFAP-GFP mouse (Fig. 4c) indicating how vinculin (white) was used to demarcate ventricle border. The ventricular lining is indicated by a dotted white line. Green, GFAP (astrocyte/NSC). Pink, Ki67 (proliferation). Blue, DAPI. Scale bar, 50 μm. **b**, Representative image of S100a6 immunofluorescent staining of old coronal section from GFAP-GFP mouse. White arrows: qNSC (S100a6 + /Ki67- and GFAP + /Ki67-); white arrowheads aNSC (S100a6 + /Ki67+ and GFAP + /Ki67+). Green, GFAP (astrocyte/NSC). Pink, S100a6 (NSC marker in the SVZ neurogenic niche). Red, Ki67 (proliferation). Blue, DAPI. Scale bar, 50 μm. **c**, NSC distance to the ventricle was calculated for qNSCs (S100a6 + /Ki67-) and aNSCs (S100a6 + /Ki67+) in coronal sections of young and old SVZs. Each dot represents the mean distance from the ventricle of 140–574 cells in 6-11 fields per section (3 sections per dot) for an individual mouse. *n* = 4 mixed-sex young mice, and *n* = 4 mixed-sex old mice, combined over 4 independent experiments. **d**, NSC distance to the ventricle was calculated for qNSCs/astrocytes (GFAP + /Ki67-) and aNSCs (GFAP + /Ki67+) in coronal sections (left) of young and old SVZs from mixed-sex GFAP-GFP mice and sagittal sections (right) of young and old SVZs from male C57BL/6 mice. Each grey dot represents the distance of a single cell from the ventricular lining. Each colored dot represents the mean distance from the ventricle of 19-231 cells in 3-7 fields per section (3 sections for coronal sections or 1 section for sagittal section) per individual mouse. Coronal sections: *n* = 4 young, and *n* = 4 old mixed-sex GFAP-GFP mice, combined over 4 independent experiments. Sagittal sections: *n* = 5 young male mice, and *n* = 5 old male C57BL/6 mice combined over 2 independent experiments. **e**, Single cell RNA-seq analysis of 28 C57BL/6 mice ranging in age from 3.3 months to 29 months (see Methods). Out of 21,458 cells, there are 4 cells expressing all markers of ependymal-repairing SVZ astrocytes (*S100b, Gfap, CD24a, Ctnnb1*) (see Methods). These cells are colored by age (4.7 months, 5.4 months, 16.83 months, and 18.87 months). Ependymal-repairing SVZ astrocytes are absent from qNSC/astrocyte, aNSC/NPC, and neuroblast clusters but are present in ependymal cell cluster. **f**, Distance to the ventricle was calculated for EdU+ aNSC/NPC (Ki67 + /DCX-) in sagittal sections of young and old SVZs 4 hours after EdU injection. Each grey dot represents the distance of a single aNSC/NPC from the ventricle. Each colored dot represents the mean aNSC/NPC distance from the ventricle of 2-35 cells from 1 section per individual mouse. *n* = 5 young male mice, and *n* = 5 old male mice, combined over 2 independent experiments. **g**, Distance to the ventricle was calculated for EdU+ neuroblasts (DCX+) in sagittal sections of young and old SVZs 4 hours after EdU injection. Each grey dot represents the distance of a single neuroblast from the ventricle. Each colored dot represents the mean neuroblast distance from the ventricle of 3-25 cells from 1 section per individual mouse. *n* = 5 young male mice, and *n* = 5 old male mice, combined over 2 independent experiments. **h**, Quantification of number of EdU+ cells counted in the SVZ (along the entire length of the ventricle) from young and old sagittal sections 4 hours post-injection of EdU. *n* = 5 young male mice, and *n* = 5 old male mice, combined over 2 independent experiments. **I**, Quantification of percent of Ki67+ cells that are EdU+ 4 hours post-injection of EdU in the SVZ (counting cells along the entire length of the ventricle). *n* = 5 young male mice, and *n* = 5 old male mice, combined over 2 independent experiments. **j**, Representative images of immunofluorescence staining of sagittal sections encompassing the SVZ, RMS, and OB regions from a young or old male C57BL/6 mouse 4 hrs or 7 days after intraperitoneal EdU injection. White inset denotes SVZ. Dotted white line indicates ventricle lining. *n* = 5 old male mice 4 hours post-injection, *n* = 3 old male mice 2 days post-injection, and *n* = 4 old male mice 7 days post-injection, combined over 2 independent experiments. Green, EdU (proliferation). Pink, Ki67 (aNSC/NPC/neuroblast); Red, DCX (neuroblast). Scale bar, 500 μm. All data are mean ± SEM. All statistical comparisons made using a two-tailed Mann-Whitney test comparing sample means. Data from independent experiments are in Source Data. Ages of animals used provided in Source Data. Source data

In coronal sections, to determine the location of quiescent and activated NSCs within the SVZ neurogenic niche (and to avoid including striatal astrocytes), we quantified cells that line the ventricle within 200 μm of the ventricle border (Fig. 4a,b, Extended Data Fig. 9a and Methods). In coronal sections from old brains, GFAP^+^/Ki67^−^ cells (qNSCs and niche astrocytes) and S100a6^+^/Ki67^−^ cells (mostly qNSCs) were located farther away from the ventricle than in young counterparts, consistent with the possibility that qNSCs (and perhaps some astrocytes) move away from their location with age (Fig. 4c,d and Extended Data Fig. 9a–d). In contrast, GFAP^+^/Ki67^+^ (aNSCs and proliferative niche astrocytes) and S100a6^+^/Ki67^+^ (mostly aNSCs) were located closer to the ventricle than in young brains (Fig. 4c,d and Extended Data Fig. 9a–d), consistent with the possibility that aNSCs may not move as far with age. Although GFAP^+^/Ki67^+^ or S100a6^+^/Ki67^+^ cells could also include repairing SVZ astrocytes that intercalate in the ependymal layer in old mice^17,63^ or reactive astrocytes^64–68^, we observed only four ependymal-repairing SVZ astrocytes and no reactive astrocytes in our single-cell RNA-seq dataset of 21,458 cells in both young and old animals^41^, suggesting that these cell types are very rare (Extended Data Fig. 9e and Supplementary Table 8).

We also determined the location of quiescent and activated NSCs (and progeny) in sagittal sections (using the RMS as an anatomical landmark; Fig. 4b). We quantified the distance to the ventricle for GFAP^+^/Ki67^−^ (qNSCs/astrocytes) and GFAP^+^/Ki67^+^ (aNSCs; Fig. 4b). We also investigated the location of cells that had recently divided by euthanizing young and old mice 4 h after intraperitoneal injection of the thymidine analog 5-ethynyl-2′-deoxyuridine (EdU), which incorporates into the DNA of replicating cells (Fig. 4e). We quantified distance to the ventricle for EdU^+^/DCX^−^/Ki67^+^ (aNSCs/NPCs), and EdU^+^/DCX^+^/Ki67^+^ or EdU^+^/DCX^+^/Ki67^−^ (neuroblasts; Fig. 4e). Consistent with results from coronal sections, we observed opposing changes in the location of qNSCs/astrocytes and aNSCs/NPCs with age in sagittal sections (Fig. 4d–g and Extended Data Fig. 9d,f). Similar to aNSCs/NPCs, neuroblasts were also located closer to the ventricles in old brain sections (Fig. 4h and Extended Data Fig. 9g). The opposing directionality between quiescent and activated NSCs (and progeny) suggests that these location changes are unlikely to be solely due to the age-dependent thinning of the ventricle (or the presence of repairing or reactive astrocytes)^15,17,63,67^. While other factors (for example, age-dependent changes to the SVZ tissue)^17^ may also contribute, the opposing location changes of qNSCs and aNSCs/NPCs (and neuroblasts) are consistent with in vitro results. Thus, aging disrupts the location of quiescent and activated NSCs in the SVZ neurogenic niche.

## Following neural stem cells and progeny along their migratory path in vivo

**Fig. 5:**
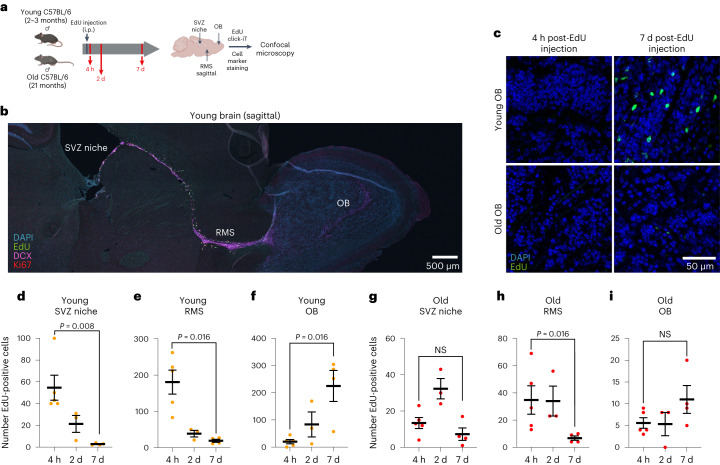
Age-dependent location defects of quiescent and activated neural stem cells and progeny in vivo in the niche, rostral migratory stream and olfactory bulb. **a**, Design of immunofluorescence experiments to assess EdU-labeled NSC localization along migratory path in vivo. Created with BioRender.com. **b**, Representative immunofluorescence staining of a sagittal section from a young male C57BL/6 mouse 4 h after EdU injection. Green, EdU; pink, DCX (neuroblast); red, Ki67 (aNSC/NPC/neuroblast); blue, DAPI. Scale bar, 500 μm. **c**, Representative images of immunofluorescence staining in sagittal sections of the OB from a young or old male C57BL/6 mouse 4 h or 7 d after EdU injection. *n* = 5 young and *n* = 5 old male mice 4 h after injection, *n* = 4 young and *n* = 4 old male mice 7 d after injection. Green, EdU; blue, DAPI. Scale bar, 50 μm. **d**–**f**, Quantification of EdU^+^ cells from young sagittal sections 4 h, 2 d and 7 d after EdU injection. Each dot represents the total number of EdU^+^ cells counted in the SVZ (along the entire length of the ventricle (**d**), the entire RMS (**e**) and the entire OB (**f**) of one sagittal section from an individual mouse. *n* = 5 young male mice 4 h after injection, *n* = 3 young male mice 2 d after injection, and *n* = 4 young male mice 7 d after injection. **g**–**i**, Quantification of EdU^+^ cells from old sagittal sections 4 h, 2 d and 7 d after EdU injection. Each dot represents the total number of EdU^+^ cells counted in the SVZ along the entire length of the ventricle (**g**), the entire RMS (**h**) and the entire OB (**i**) in one sagittal section from an individual mouse. *n* = 5 old male mice 4 h after injection, *n* = 3 old male mice 2 d after injection, and *n* = 4 old male mice 7 d after injection. In **b**–**i**, data were combined over two independent experiments. All data are the mean ± s.e.m. All statistical comparisons were made using a two-tailed Mann–Whitney test. Source data

To follow NSCs and progeny along their migratory path in vivo, we quantified the location of newborn NSCs and their progeny (NPCs, neuroblasts) in the SVZ niche, along the RMS, and in the OB during aging. To this end, we injected young and old mice with EdU to label and trace aNSCs and their progeny (Fig. 5a). We verified that EdU labeling efficiency was similar in young and old individuals (Extended Data Fig. 9h,i). We then assessed the number of EdU-positive (EdU^+^) cells in the SVZ neurogenic niche, the RMS and distal destination (OB), at a short (4 h), middle (2 d) or longer (7 d) time point after EdU injection (Fig. 5a,b and Extended Data Fig. 9j). In young individuals, EdU^+^ cells were numerous in the niche and along the RMS 4 h after EdU injection (Fig. 5d,e and Extended Data Fig. 9j). After 7 d, young animals showed a dramatic reduction of EdU^+^ cells in the SVZ and RMS, and a corresponding increase in EdU^+^ cells in the OB, consistent with mobilization of labeled cells out of the SVZ neurogenic niche and clearance from the RMS toward the OB (Fig. 5c–f and Extended Data Fig. 9j). In old individuals, EdU^+^ cells were less numerous overall, as expected^17–19,69^ (Fig. 5g–i and Extended Data Fig. 9h). Interestingly, after 7 d, old animals showed no reduction of EdU^+^ cells in the SVZ neurogenic niche and no corresponding increase in EdU^+^ cells in the OB (Fig. 5c,g,i), consistent with the possibility that there is reduced mobilization of NSCs (and progeny) out of the niche. There was some clearance of EdU^+^ cells through the old RMS after 7 d in old animals (Fig. 5h), suggesting that old neuroblasts may retain motility in the RMS^55^ but migrate more slowly than young neuroblasts. While this experimental design is static and integrates cell migration as well as other factors (for example, EdU dilution, differences in cell cycle and survival), these in vivo results are consistent with our in vitro observations and suggest that aging could decrease the ability of aNSCs and progeny (NPCs, neuroblasts) to leave the neurogenic niche and reach their distal destination.

## ROCK inhibition removes force-producing adhesions in old activated neural stem cells

**Fig. 6:**
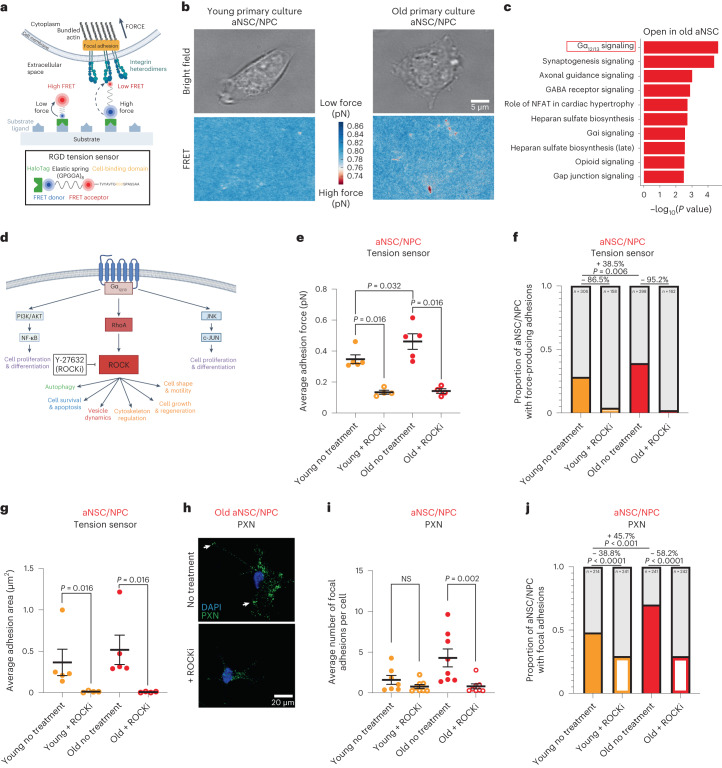
Molecular tension sensors reveal an increase in force-producing adhesions in old activated neural stem cells/neural progenitor cells that can be eliminated by ROCK inhibition. **a**, Diagram of RGD molecular tension sensor. **b**, Representative images of young and old cultured aNSCs/NPCs taken with brightfield (top) and traction map of FRET efficiency (bottom). Colored bar represents FRET efficiency where low FRET efficiency indicates high force (red) and high FRET efficiency indicates low force (blue). Scale bar, 5 μm. **c**, Top ten canonical pathways enriched for genes associated with differentially accessible peaks that open with age in aNSCs (FDR < 0.05) generated by IPA and ranked by *P* value (one-sided Fisher’s exact test). ATAC-seq peaks annotated with their nearest gene using ChIPSeeker. **d**, Gα_12/13_ signaling pathway (adapted from IPA diagram and previous work). **e**, Average adhesion force (pN) exhibited by young and old cultured aNSCs/NPCs treated with H_2_O vehicle (solid circles) or 10 μM ROCKi (open circles). Each dot represents the average force produced by one cell (15–89 cells per dot) in a primary culture derived from an individual mouse. **f**, Proportion (colored bars) of young and old cultured aNSCs/NPCs exhibiting force-producing adhesion patterns when treated with H_2_O (solid bars), or ROCKi (open bars). Same experiment as in **e**. *P* values were calculated using a two-sided Fisher’s exact test. **g**, Average adhesion area of force-producing adhesions of young and old cultured aNSCs/NPCs treated with H_2_O vehicle (solid circles) or 10 μM ROCKi (open circles). Each dot represents the average adhesion area of force-producing adhesions from a single cell (15–89 cells per dot) in a primary culture derived from an individual mouse. Same experiment as in **e**. **b**,**e**,**f**,**g**, *n* = 5 young male mice and *n* = 5 old male mice (no treatment), and *n* = 4 young male mice and *n* = 4 old male mice (ROCKi), combined over three independent experiments. **h**, Representative immunofluorescence staining of old cultured aNSCs/NPCs treated with H_2_O vehicle (no treatment) or 10 μM ROCKi. Green, PXN (focal adhesions). Blue, DAPI. Arrows indicate PXN localization to focal adhesions. Scale bar, 20 μm. **i**, Average number of focal adhesions (marked by PXN) exhibited by young and old cultured aNSCs/NPCs treated with H_2_O vehicle (solid circles) or 10 μM ROCKi (open circles). Each dot represents the average number of focal adhesions per cell from a primary culture (30 cells per dot) derived from an individual mouse. **j**, Proportion (colored bars) of young and old cultured aNSCs/NPCs exhibiting focal adhesions (marked by PXN) when treated with H_2_O vehicle (solid bars) or 10 μM ROCKi (open bars) exhibiting focal adhesions. Same experiment as in **i**. *P* values calculated using a two-sided Fisher’s exact test. **h**–**j**, *n* = 7 young and *n* = 8 old male mice (no treatment), and *n* = 8 young and *n* = 8 old male mice (ROCKi treatment), combined over two independent experiments. In **e**, **g** and **i**, data are the mean ± s.e.m. All statistical comparisons were made using a two-tailed Mann–Whitney test unless otherwise stated. Figures in **a** and **d** were created with BioRender.com. Source data

**Extended Data Fig. 10:**
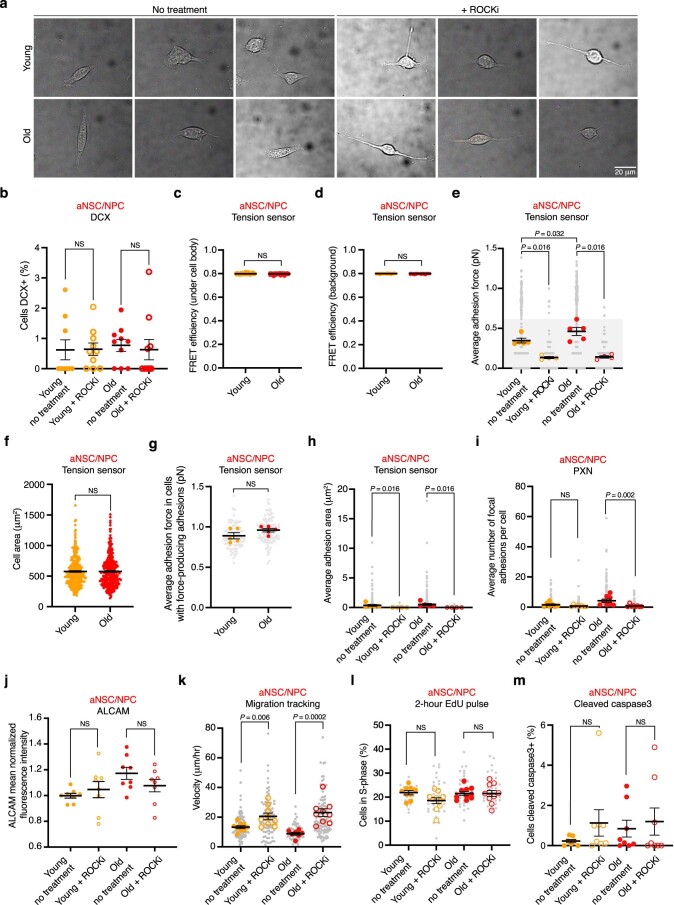
Effect of ROCK inhibitor in vitro. **a**, Representative brightfield images of young and old aNSCs/NPCs on RGD molecular tension sensors with H_2_O vehicle or 10 μM ROCKi. **b**, Percent cells that are positive for DCX staining in young and old aNSCs/NPCs treated with H_2_O vehicle (solid dots) or 10 μM ROCKi (open circles). Each dot represents percent cells DCX-positive in a given field (containing 38-349 cells) for an individual animal (4-5 fields per animal). *n* = 2 young male mice and *n* = 2 old male mice (no treatment), and *n* = 2 young male mice and *n* = 2 old male mice (+ROCKi), combined over 1 experiment. **c**, FRET efficiency values underneath cell bodies of young and old cultured aNSC/NPC on RGD molecular tension sensors. Same experiment as Fig. 6e. **d**, Background FRET efficiency values of young and old cultured aNSCs/NPCs on RGD molecular tension sensors. **e**, Quantification of average adhesion force (pN) exhibited by young and old cultured aNSCs/NPCs treated with H_2_O vehicle (solid dots) or 10 μM ROCKi (open circles) determined using RGD molecular tension sensors. Each grey dot represents adhesion force produced by one cell. Each colored dot represents the average force produced by one cell (15– 89 cells per dot) in a primary culture derived from an individual mouse. Shading delineates the bimodal populations of cells with strong adhesions (white) or weak or no adhesions (grey). **f**, Cell areas of young and old cultured aNSCs/NPCs determined using RGD molecular tension sensors. Same experiment as in (e). **g**, Quantification of average adhesion force (pN) exhibited by young and old cultured aNSCs/NPCs that have at least one force-producing adhesion determined using RGD molecular tension sensors. Same experiment as in (e). Each grey dot represents adhesion force produced by one cell. Each colored dot represents the average force produced by one cell in cells that have at least one force-producing adhesion (8-29 cells per dot) in a primary culture derived from an individual mouse. *n* = 5 young male mice and *n* = 5 old male mice, combined over 3 independent experiments. **h**, Quantification of average adhesion area under force-producing adhesions (μm^2^) of young and old cultured aNSCs/NPCs treated with H_2_O vehicle (solid dots) or 10 μM ROCKi (open circles) determined using RGD molecular tension sensors. Each grey dot represents adhesion area of force-producing adhesion from a single cell. Each colored dot represents the average adhesion area of force-producing adhesions from a single cell (15–89 cells per dot) in a primary culture derived from an individual mouse. Same experiment as in (e). **c,d,f** *n* = 306 young aNSCs/NPCs, and *n* = 298 old aNSCs/NPCs, combined over 3 independent experiments. **e,h** *n* = 5 young male mice and *n* = 5 old male mice (no treatment), *n* = 4 young male mice and *n* = 4 old male mice (ROCKi treatment), combined over 3 independent experiments. **i**, Quantification of average number of focal adhesions, determined using paxillin (PXN) staining, exhibited by young and old cultured aNSCs/NPCs treated with H_2_O vehicle (solid dots) or 10 μM ROCKi (open circles). Each grey dot represents number of focal adhesions per cell. Each colored dot represents the average number of focal adhesions per cell from a primary culture (30 cells per dot) derived from an individual mouse. *n* = 7 young male mice and *n* = 8 old male mice (no treatment) and *n* = 8 young male mice and *n* = 8 old male mice (ROCKi treatment), combined over 2 independent experiments. **j**, Quantification of ALCAM fluorescence intensity of young and old aNSCs/NPCs treated with H_2_O vehicle (solid dots) or 10 μM ROCKi (open circles). Each dot represents mean-normalized fluorescence intensity of 30 fields (each containing 1-3 cells) in a primary culture derived from an individual mouse, normalized by experiment and cell size. *n* = 8 young male mice and *n* = 8 old mice (no treatment) and *n* = 8 young male mice and *n* = 8 old male mice (ROCKi treatment), combined over 2 independent experiments. **k**, Quantification of migration speed of young and old aNSC/NPC ± treatment with 10 μM ROCKi. Each grey dot represents average velocity of a single cell over a 20-hour period. Each colored dot represents the average velocity over a 20-hour period of cells (2-28) from one culture derived from one individual mouse. *n* = 9 young male mice and *n* = 7 old male mice (solid dots) and *n* = 9 young male mice and *n* = 9 old male mice (open circles), combined over 3 independent experiments. **l**, Percent cells that are EdU-positive (S-phase) after a 2-hour pulse for young and old aNSCs/NPCs treated with H_2_O vehicle (solid dots) or 10 μM ROCKi (open circles). Each grey dot represents percent of cells in given field that are EdU-positive. Each colored dot represents average percent cells EdU-positive for an individual mouse (average of 5-7 fields containing at least 100 cells each). *n* = 9 young male mice and *n* = 10 old male mice (no treatment), and *n* = 10 young male mice and *n* = 10 old male mice (ROCKi treatment) combined over 3 independent experiments. **m**, Percent cells that are positive for cleaved-caspase 3 staining in young and old aNSCs/NPCs treated with H_2_O vehicle (solid dots) or 10 μM ROCKi (open circles). Each dot represents average percent cells cleaved caspase3-positive for an individual mouse (average of 5 fields containing at least 100 cells). *n* = 7 young male mice and *n* = 8 old male mice (no treatment), and *n* = 8 young male mice and *n* = 8 old male mice (+ROCKi), combined over 2 independent experiments. All data are mean ± SEM. All statistical comparisons made using a two-tailed Mann-Whitney test comparing sample means unless otherwise stated. Data from independent experiments are in Source Data. Source data

We next used molecular tension sensors to assess the cellular properties underlying the increased adhesion observed in aNSCs and progenitors during aging, focusing on cell–matrix adhesion. To directly visualize the mechanical forces exerted by aNSCs/NPCs interacting with their extracellular matrix substrate, we leveraged Förster resonance energy transfer (FRET)-based molecular tension sensors (Fig. 6a)^52,70^. Molecular tension sensors can reveal cellular forces on surfaces coated with synthetic arginine–glycine–aspartate (RGD) peptides known to bind integrins and mediate adhesion^52^ at single-molecule resolution, with higher forces corresponding to a reduction in FRET efficiency (Fig. 6a,b and Extended Data Fig. 10a,b).

FRET measurements revealed that aNSCs/NPCs primarily exert force through discrete adhesion complexes at their periphery (Fig. 6b and Extended Data Fig. 10c,d), consistent with PXN staining for focal adhesions (Fig. 2k). To investigate how aging affects NSC adhesion, we quantified the adhesive force patterns for young and old cultured aNSCs/NPCs. Old aNSCs/NPCs exhibited higher average adhesion force (*P* = 0.032; Fig. 6e and Extended Data Fig. 10e), without changes in cell size (Extended Data Fig. 10f), compared to young aNSCs/NPCs. Furthermore, old aNSCs/NPCs were more likely to exhibit force-producing adhesions than young aNSCs/NPCs (Fig. 6f; *P* = 0.006). In the subset of cells that had force-producing adhesions, old aNSCs/NPCs showed a (non-significant) increase in average adhesion force (Extended Data Fig. 10g), in line with increased immunostaining with PXN, a marker of focal adhesions (Fig. 2m and Extended Data Fig. 7k,m). Thus, aging not only increases the proportion of aNSCs/NPCs that exhibit focal adhesions but also increases the average adhesion strength of individual old aNSCs/NPCs.

We sought to identify a molecular target to counter increased adhesion strength observed in old aNSCs, as a way to restore age-related mobilization of old aNSCs and progeny out of the niche and subsequently improve neurogenesis in the old brain. Focal adhesions are regulated by several pathways, including via the Rho and Rho-associated protein kinase (ROCK) pathway^71^. Indeed, Ingenuity Pathway Analysis (IPA)^72^ showed that the most enriched signaling pathway associated with the chromatin accessibility changes in old aNSCs/NPCs was Gα_12/13_ signaling (Fig. 6c and Supplementary Table 6), a pathway that regulates cell adhesion in part via ROCK^71,73^ (Fig. 6d).

To determine if the age-related increase in cell adhesion exhibited by old aNSCs could be reversed via modulation of the ROCK pathway, we targeted ROCK with Y-27632, a small-molecule inhibitor^59,74–81^ in cultured aNSCs/NPCs. Quantification of force patterns at single-molecule resolution using RGD molecular tension sensors revealed that treating old aNSCs/NPCs with the ROCK inhibitor (ROCKi) Y-27632 eliminated force-producing adhesions and decreased adhesion area (Fig. 6e,g and Extended Data Fig. 10e,h) in both young and old aNSCs/NPCs. ROCKi also decreased the proportion of cells with force-producing adhesions as assessed by tension sensors (Fig. 6f) as well as focal adhesions (marked by PXN) in young and old aNSCs/NPCs with a greater effect on old aNSCs/NPCs (Fig. 6h–j and Extended Data Fig. 10i). ROCKi did not affect the expression of ALCAM protein (Extended Data Fig. 10j), suggesting ROCKi acts downstream of some of the age-related adhesion gene expression changes. Thus, ROCK inhibition eliminates force-producing adhesions and focal adhesions in aNSCs/NPCs.

## ROCK inhibition boosts old activated neural stem cell/neural progenitor cell migration in vitro

**Fig. 7:**
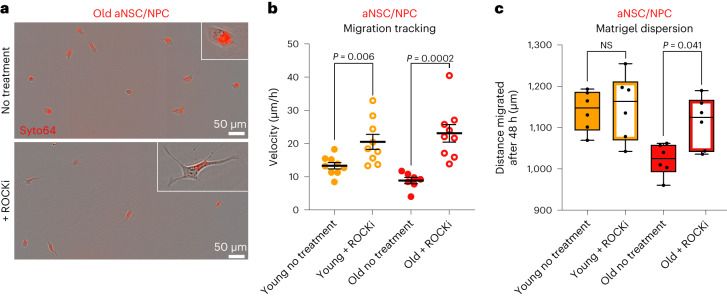
ROCK inhibition boosts migration speed in activated neural stem cells/neural progenitor cells cultured from aged brains. **a**, Representative images of old cultured aNSCs/NPCs 12 h after plating onto migration plates treated with H_2_O vehicle (no treatment) or 10 μM ROCKi. Inset displays a representative magnified cell. Scale bars, 50 μm. **b**, Migration speed of young and old aNSCs/NPCs treated with H_2_O vehicle (solid circles) or with 10 μM ROCKi (open circles). Each dot represents average velocity over a 20-h period of cultured cells (2–28) derived from one individual mouse. **a**,**b**, *n* = 9 young and *n* = 7 old male mice (no treatment), and *n* = 9 young and *n* = 9 old male mice (ROCKi treatment), combined over three independent experiments. Data are the mean ± s.e.m. **c**, Cell dispersion through Matrigel after 48 h by young and old aNSCs/NPCs treated with H_2_O vehicle (solid bars) or 10 μM ROCKi (open bars). Each dot represents the average dispersion distance through Matrigel after 48 h of cultured aNSCs/NPCs derived from an individual mouse. *n* = 6 young and *n* = 6 old male mice for treated and untreated conditions, combined over two independent experiments. For each biological replicate, 1–4 technical replicates were evaluated, and dispersion distance was averaged. Box plots display the median and lower and upper quartile values. Whiskers indicate the minimum and maximum within 1.5 times the interquartile range. All statistical comparisons were made using a two-tailed Mann–Whitney test. Source data

We next asked if ROCK inhibition improves the migration properties of old aNSCs in vitro. In cultured aNSCs/NPCs, ROCKi treatment resulted in improved migration speed in both young and old aNSCs/NPCs (Fig. 7a,b, Extended Data Fig. 10k and Supplementary Videos 1 and 2). Furthermore, ROCKi rescued the age-related decline in old aNSC/NPC dispersion through Matrigel but had no effect on young aNSC/NPC dispersion (Fig. 7c), consistent with the observation that ROCKi had a more profound effect on decreasing focal adhesions in old aNSCs/NPCs than in young counterparts (Fig. 6i,j). We verified that ROCKi induced cell morphological changes^79^ (Extended Data Fig. 10a)—consistent with its ability to improve cell migration—but it did not overtly affect aNSC/NPC differentiation, proliferation, or survival under these culture conditions (Extended Data Fig. 10b,l,m). Together, these results indicate that ROCK inhibition boosts the migratory ability of aNSCs/NPCs cultured from old mice.

## ROCK inhibition improves neurogenesis in vivo in old mice

**Fig. 8:**
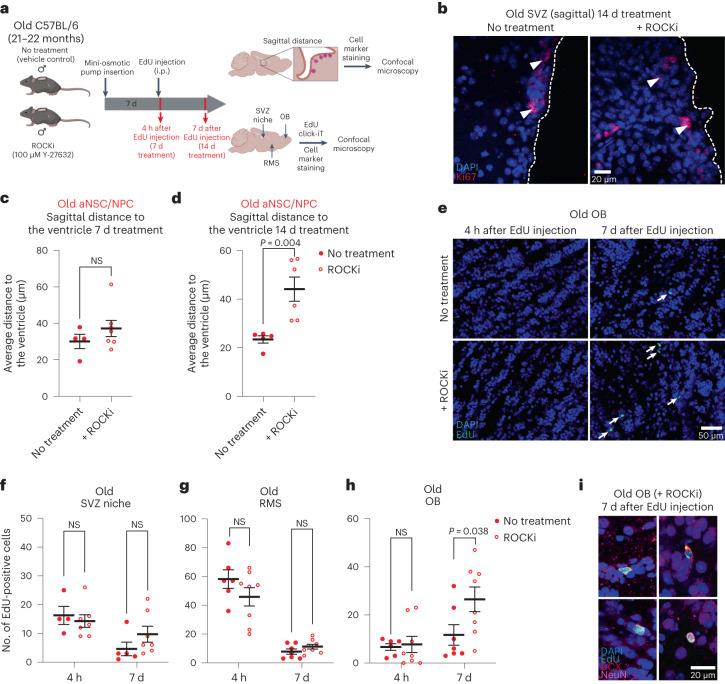
ROCK inhibition improves in vivo neurogenesis in old mice. **a**, Design of in vivo ROCKi immunofluorescence experiments to assess EdU-labeled NSC localization along migration path. Created with BioRender.com. **b**, Representative images of immunofluorescence staining in sagittal sections of the SVZ of old vehicle control (no treatment) and ROCKi-treated mice after 14 d of treatment. The dashed line represents the ventricle border. Arrowheads indicate aNSCs/NPCs. Red, Ki67 (proliferation); blue, DAPI. Scale bar, 20 μm. **c**,**d**, Sagittal distance to the ventricle for aNSCs/NPCs in old SVZs treated with vehicle control (solid circles) or ROCKi (open circles) for 7 d (4 h after EdU injection; **c**) and 14 d (7 d after EdU injection; **d**). Each dot represents the mean distance from the ventricle per mouse. For **c**, *n* = 4 old (no treatment) and *n* = 7 old male mice (ROCKi); for **d**, *n* = 5 old (no treatment) and *n* = 6 old male mice (ROCKi). **e**, Representative images of immunofluorescence staining of the old OB treated with vehicle control or ROCKi 4 h or 7 d after EdU injection. Arrows indicate EdU^+^ cells. Green, EdU; blue, DAPI. Scale bar, 50 μm. **f**–**h**, Quantification of EdU^+^ cells in the SVZ (**f**), RMS (**g**) and OB **(h)** from sagittal sections 4 h and 7 d after EdU injection in old mice treated with vehicle control (no treatment, solid circles) or ROCKi (open circles). Each dot represents the number of EdU^+^ cells counted in one sagittal section from a single mouse. For the 4-h time point, *n* = 4 (SVZ, no treatment), *n* = 6 (RMS and OB, no treatment), *n* = 7 (SVZ, ROCKi) and *n* = 8 (RMS and OB, ROCKi) old male mice. For the 7-d time point, *n* = 5 (SVZ, no treatment), *n* = 7 (RMS and OB, no treatment), *n* = 7 (SVZ, ROCKi) and *n* = 8 (RMS and OB, ROCKi) old male mice. **i**, Representative immunofluorescence images of old OB 7 d after EdU injection. EdU^+^ cells in the OB are DCX^+^ (top) or NeuN^+^ (bottom). *n* = 8 old male mice. Green, EdU; red, DCX (neuroblast/immature neuron); pink, NeuN (neuron); blue, DAPI. Scale bar, 20 μm. All data are the mean ± s.e.m. In **b**–**i**, data were combined over two independent experiments. All statistical comparisons were made using a two-tailed Mann–Whitney test. Source data

To determine how ROCKi treatment in the old neurogenic niche impacts the location of cells in the niche and long-distance neurogenesis in vivo, we delivered ROCKi (or vehicle control) via a mini-osmotic pump into the lateral ventricles of old mice, in close proximity to the SVZ neurogenic niche (Fig. 8a). Seven days after ROCKi delivery, we injected old mice with EdU and quantified EdU^+^ cells in the SVZ neurogenic niche, RMS and OB, 4 h or 7 d after EdU injection (corresponding, respectively, to 7 d and 14 d of ROCKi treatment; Fig. 8a). We quantified both the location of aNSCs/NPCs in the neurogenic niche in sagittal sections and the number of EdU^+^ cells in the niche and at different locations along the neurogenic migration route (Fig. 8a), as we had previously done (Fig. 5). Interestingly, ROCKi delivery in the ventricles significantly increased aNSC/NPC distance from the ventricle 14 d after treatment (7 d after EdU injection) in old mice (Fig. 8b–d)—a feature associated with young neurogenic niches (Fig. 4). These results suggest that ROCK inhibition could restore at least in part the location of aNSCs/NPCs in the neurogenic niche of old animals.

Does ROCK inhibition improve neurogenesis in old individuals? While ROCKi did not affect the number of EdU^+^ cells in the SVZ neurogenic niche or RMS (Fig. 8f,g), ROCKi delivery in the ventricles led to a significant increase in EdU^+^ cells in the OB of old mice 7 d after EdU injection (Fig. 8e,h). These EdU^+^ cells in the OB exhibited markers of neuroblasts and neurons (DCX, NeuN; Fig. 8i). While this assay integrates changes in proliferation, cell survival and differentiation, in addition to cell migration and adhesion, these results indicate that ROCK inhibition in the neurogenic niche restores the age-related defects in location of old aNSCs/NPCs in the niche and boosts neurogenesis at a distance in old brains. Thus, inhibition of the ROCK pathway could be a strategy to improve the migratory property of old aNSCs (and their progeny) and to boost neurogenesis in old brains.

## Discussion

Our study identifies genome-wide changes to the global chromatin landscape of five freshly isolated cell types from the neurogenic niche of young and old animals. This analysis uncovers previously uncharacterized opposing changes in chromatin dynamics in quiescent and activated NSCs during aging in vivo—with some changes observed in aNSCs preserved in downstream progeny. Interestingly, many of these opposing chromatin changes in quiescent and activated NSCs affect genes in adhesion and migration pathways and are accompanied by corresponding expression changes in these genes. Functionally, quiescent and activated NSCs exhibit opposing changes in adhesion with age, with old quiescent NSCs becoming less adherent and old activated NSCs becoming more adherent as well as less migratory compared to young counterparts, and we identify some of the adhesion molecules involved in these age-dependent changes. Using molecular tension sensors, we observed increased force production in old activated NSCs—a phenotype that could explain at least in part the adhesion changes in aNSCs with age. We also uncovered the ROCK pathway as a potential target to revert the defects in adhesion and migration of old aNSCs. A small-molecule ROCK inhibitor (ROCKi) could decrease force production in old aNSCs and restore part of their migration ability in vitro. In vivo, ROCKi injected in the ventricles—in the vicinity of the neurogenic niche—reverses at least part of the age-dependent changes in the location of NSCs (and progeny) within the niche and boosts neurogenesis in old individuals. Our results have important implications for the role of NSC adhesion and migration during aging and point to ROCK as a potential therapeutic target to restore age-dependent defects that occur in old individuals.

With age, the chromatin landscape of qNSCs becomes more closed, whereas that of aNSCs becomes more open. The closing of chromatin regions with age in qNSCs is consistent with observations in cultured NSCs^31^ and hair follicle stem cells^82^, and with findings of increased repressive chromatin marks such as trimethylated histone H3 Lys27 (H3K27me3) in quiescent stem cells from other niches^83^, including muscle satellite cells^84,85^ and hematopoietic stem cells^86^ during aging. In contrast, the chromatin landscape of aNSCs generally becomes more permissive with age, consistent with the observation that reducing the repressive 5-hydroxymethylcytosine (5hmC) mark in hippocampal NPCs mimics age-dependent defects^26^. Interestingly, many of these age-related chromatin changes occur in regulatory regions of cell adhesion and migration genes. The migratory properties of NSCs and neuroblasts have started to be examined with age^55–59^ and in the context of innervating distal tumors^87^. Recent live-cell imaging of SVZ whole-mount preparations showed that aNSC and NPC migration distance and speed decreased with age^59^. However, the changes in migration and adhesion in qNSCs during aging have remained largely unknown. Adhesion molecules have been shown to play important roles in both quiescent and activated stem cell function^29,60,61,88–91^, and targeting some of the adhesion pathways could be beneficial to counter age-dependent functional decline. Our study reveals a dichotomy between changes to the adhesive and migratory properties of quiescent and activated NSCs with age that could be a result of both intrinsic and extrinsic changes. While our findings in cultured NSCs show intrinsic changes to cellular adhesion and migration pathways with age, extrinsic changes likely also play a key role. In line with this, age-related differences in the biomechanical properties of regenerative niches, such as stiffness, have been shown to extrinsically induce age-related phenotypes^92^. It is also possible that mechanical memory of the in vivo niche could manifest in cell-autonomous changes in cultured NSCs. This has been observed with cultured mesenchymal stem cells^93,94^ and epithelial cells^95^ that retain information from past physical environments. Given that mechanical cell deformations and niche stiffness can affect chromatin states and promoter accessibility^82,96–98^, epigenomic profiling might be especially sensitive to identifying changes in the regulation of adhesion and migration in stem cells and their progeny. Understanding the contribution of intrinsic and extrinsic responses, and how they influence each other, will be critical to uncover strategies to boost regeneration during aging.

Our results implicate ROCK, a key regulator of cytoskeletal dynamics^71^, in modulating NSC migration during aging. ROCK inhibition impacts migration in a variety of cells, including myoblasts, glioma cells and microglia with different effects^99–106^. The effect of ROCK inhibition on aNSCs/NPCs and neurogenesis has been previously examined, with varying results. Some studies indicate that short periods of ROCK inhibition in culture and ex vivo cause decreased migration speed in young and postnatal neuroblasts^107^ and human embryonic NSCs^108^. More recently, short-term ROCK inhibition was found to decrease NSC migration speed in SVZ whole-mount preparations from young adult mice^59^, although effects in old mice were not tested. Here, we have instead found that ROCK inhibition boosts old NSC migration in vitro and improves neurogenesis in old mice in vivo. The effects of ROCKi on NSC migration may depend on length of treatment, interaction between age and cell context, and interaction with other cell types. In line with length of treatment being an important factor, other studies show that long periods of ROCK inhibition increase NSC migration^79,109^ and hippocampal neurogenesis^74^ in young animals. Furthermore, cell migration reflects a balance between cell adhesion, cytoskeletal traction generation and actin protrusive dynamics^110,111^. Hence, decreasing cytoskeletal contractility via ROCK inhibition may shift cells (such as old aNSCs) with excessive contractility and adhesion toward a more optimal state for migration, while having the opposite effect on less contractile cells (such as young aNSCs). Finally, in our study, ROCKi was administered directly to the ventricles and some of the beneficial effects on NSCs could result from indirect improvement to niche cells. In the future, tracking stem cells as a function of space and time, coupled with in vivo imaging of the niche^112^, will be critical to better understand the specific effect of ROCK inhibition on neurogenesis. While ROCK has pleiotropic roles in aspects other than migration (for example, autophagy, apoptosis and vesicle dynamics), this protein kinase may be a promising target in the aging brain to restore aspects of migration notably in cases of injury and neurodegenerative disease^113^. ROCK inhibitors are well tolerated in humans and have begun to be studied in the context of neurodegenerative disease^113^ and stroke^114,115^, and could potentially be used to ameliorate other age-dependent defects in the aging brain.

## Methods

### Laboratory animals

For in vivo ATAC-seq libraries, an equal number of male and female GFAP-GFP (FVB/N background) mice^32^ were pooled and used. For ATAC-seq libraries generated from cultured NSCs, the SVZs from one male and one female C57BL/6 mouse obtained from the National Institute on Aging (NIA) Aged Rodent colony were pooled and used. For gene knockout experiments, male and female Rosa26-Cas9 knock-in mice^116^ (C57BL/6 background) were used (https://www.jax.org/strain/024858/). For immunohistochemistry of coronal brain sections, male and female GFAP-GFP mice^32^ were used. For all other experiments, male C57BL/6 mice obtained from the NIA Aged Rodent colony were used. In all cases, mice were habituated for more than 1 week at Stanford before use. At Stanford, all mice were housed in either the Comparative Medicine Pavilion or the Neuro Vivarium, and their care was monitored by the Veterinary Service Center at Stanford University under the Institutional Animal Care and Use Committee protocol 8661.

### ATAC-seq library generation from freshly isolated cells

We used FACS to freshly isolate populations of endothelial cells, astrocytes, qNSCs, aNSCs and NPCs from GFAP-GFP (FVB/N background) animals^32^. This GFAP-GFP strain expresses GFP under the control of the human GFAP promoter and has been used to isolate NSCs by FACS^33,34,117,118^.

We microdissected and processed the SVZs from young (3–5 months old) and old (20–24 months old) GFAP-GFP mice following a previously described protocol^33^ with the addition of negative gating for CD45 (hematopoietic lineage) and sorting of endothelial cells (CD31^+^) as described^34^ (Extended Data Fig. 1a). All FACS sorting was performed at the Stanford FACS facility on a BD Aria II sorter (BD FACSDiva, v8.0.1), using a 100-μm nozzle at 13.1 pounds per square inch, and FlowJo (v8) software was used for data analysis. Due to the rarity of NSC lineage cells, we pooled sorted cells from two young male and two young female GFAP-GFP mice for the young conditions (3–5 months old), and from three old male and three old female GFAP-GFP mice for the old conditions (20–24 months old). For each respective library, we sorted 2,000 astrocytes (CD45^−^/CD31^−^/GFAP-GFP^+^/PROM1^−^/EGFR^−^), 2,000 qNSCs (CD45^−^/CD31^−^/GFAP-GFP^+^/PROM1^+^/EGFR^−^; with the exception of a single library that only had 1,670 cells), 800–1,000 aNSCs (CD45^−^/CD31^−^/GFAP-GFP^+^/PROM1^+^/EGFR^+^), 2,000 NPCs (CD45^−^/CD31^−^/GFAP-GFP^−^/EGFR^+^) and 2,000 endothelial cells (CD45^−^/CD31^+^) from GFAP-GFP animals for ATAC-seq (Supplementary Table 1). Young and old cells of the five cell types were sorted into 150 μl of NeuroBasal-A medium (Gibco, 10888-022) with penicillin–streptomycin–glutamine (Gibco, 10378-016) and 2% B27 minus vitamin A (Gibco, 12587-010) in a 96-well V-bottomed plate (Costar, 3894) and spun down at 300*g* for 5 min at 4 °C. Sorted cells were washed with 100 μl ice-cold PBS (Corning, 21-040-CV), male and female cells were pooled by age and cell type, and then spun down at 300*g* for 5 min at 4 °C. Next, 50 μl of lysis buffer (10 mM Tris HCl pH 7.4 (Sigma, T2194), 10 mM NaCl, 3 mM MgCl_2_ (Ambion, AM9530G), 0.10% NP-40 (Thermo, 85124)) was added to each well (alternating between young and old wells) and was immediately spun down at 500*g* for 10 min at 4 °C. Lysis buffer was carefully aspirated and 5 μl of transposition mix (2.5 μl 2× Tagment DNA (TD) buffer, 2.25 μl nuclease-free H_2_O, 0.25 μl Tn5 transposase (Illumina, FC-121-1030)) was added to each well and pipetted 6× to resuspend nuclei. Cells were incubated for 30 min at 37 °C in a sealed 96-well plate and then briefly spun down at 500*g* for 1 min to account for evaporation. Transposed DNA was then purified using the Zymo DNA Clean & Concentrator kit (Zymo, D4014) and eluted in 20 μl of nuclease-free H_2_O. PCR amplification and subsequent qPCR monitoring was performed as previously described in the original ATAC-seq protocol^35^. ATAC-seq libraries from young and old cells were amplified with 11–14 PCR cycles and then purified using the Zymo DNA Clean & Concentrator kit (Zymo, D4014) and eluted in 15 μl of nuclease-free H_2_O. See ‘Library sequencing and ATAC-seq quality control of in vivo and cultured NSCs’ for details on sequencing of ATAC-seq libraries.

The following antibodies were used: CD31-PE (eBioscience, Clone 390, 12-0311-81, 4338515; 1:50 dilution), CD45-Brilliant Violet 605 (BioLegend, clone 30-F11, 103139, B264625; 1:50 dilution), CD24-eFluor 450 (eBioscience, Clone M1/69, 48-0242-82, 4311339; 1:400 dilution), EGF-Alexa 647 (Molecular probes, E-35351, 1526644; 1:300 dilution), Prominin-1-biotin (Invitrogen, clone 13A4, 13-1331-82, 2233571; 1:400 dilution) and Streptavidin-PEcy7 (eBioscience, 25-4317-82, 4290713; 1:1000 dilution).

### Analysis of potential changes in FACS markers with age

To assess whether the protein levels of the FACS markers used to freshly isolate NSCs from the SVZ neurogenic niche changed with age, we used the same sorted cell populations from young and old GFAP-GFP animals described above in ‘ATAC-seq library generation from freshly isolated cells’ and used FlowJo (v10.7.1) to export the compensated scaled fluorescence values for NSC markers (GFAP-GFP, PROM1, EGFR) from all sorted propidium iodide-negative (live) cells, qNSCs (CD45^−^/CD31^−^/GFAP-GFP^+^/PROM1^+^/EGFR^−^), and aNSCs (CD45^−^/CD31^−^/GFAP-GFP^+^/PROM1^+^/EGFR^+^). Fluorescence values for these three channels were normalized to the young mean for an experiment and the means for each animal were then visualized using box plots with R (v3.5.2). Statistical comparisons between sample means were performed in R using a two-tailed Mann–Whitney test.

For all live cells, there were significant changes in EGFR, GFAP and PROM1 expression with age (Extended Data Fig. 1b), likely reflecting changes in cell-type composition with age in the SVZ neurogenic niche^7,22,23,119^. For FACS-purified qNSCs, there was no age-related change in EGFR, GFAP or PROM1 expression (Extended Data Fig. 1c). For FACS-purified aNSCs, there was no change in GFAP or PROM1 expression, but a slight increase in EGFR expression with age (Extended Data Fig. 1d).

Previously, we verified that young and old FACS-isolated SVZ astrocytes, qNSCs, aNSCs and NPCs expressed well-established cell-type markers (validated by RT–qPCR), showed expected cell cycle characteristics^34^, and were similar to NSCs isolated with a different FACS scheme using the marker GLAST^117^.

### Primary neural stem cell culture

To obtain primary cultures of quiescent and activated NSCs from young and old mice for ATAC-seq, we used a previously described primary culture protocol^29^, which does not depend on FACS to isolate NSCs. We microdissected and pooled SVZs from pairs of male and female C57BL/6 animals at a young age (3 months old) or an old age (23 months old) obtained from the NIA Aged Rodent colony. To obtain primary cultures of quiescent and activated NSCs from young and old mice for immunofluorescence staining and detachment, migration and tension sensor assays, we isolated NSCs from SVZs from a single young male (2.5–4 months old) or a single old male (20–25 months old) C57BL/6 animal from the NIA Aged Rodent colony. For CRISPR–Cas9 experiments, SVZs from a single young male or female (3.3–5.2 months old) or old male or female (21.8–25.3 months old) Rosa26-Cas9 (ref. ^116^; C57BL/6 background) mice were used. In all cases, microdissected SVZs were finely minced, suspended in 5 ml of PBS + 0.1% gentamicin (Thermo Fisher, 15710064) and spun down at 300*g* for 5 min at room temperature. We then dissociated SVZs by enzymatic digestion using 5 ml of HBSS (Corning, 21-021-CVR) with 1% penicillin–streptomycin–glutamine (Gibco, 10378-016), 1 U ml^−1^ Dispase II (STEMCELL Technologies, 07913), 2.5 U ml^−1^ papain (Worthington Biochemical, LS003126) and 250 U ml^−1^ DNAse I (D4527, Sigma-Aldrich), vortexed briefly, and left at 37 °C for 40 min on a rotator. Following digestion, the samples were spun down at 300*g* for 5 min at room temperature and resuspended in 5 ml of NeuroBasal-A medium (Gibco, 10888-022) with 1% penicillin–streptomycin–glutamine (Gibco, 10378-016) and 2% B27 minus vitamin A (Gibco, 12587-010) and triturated repeatedly (×20) with 2–3 washes. Single-cell suspensions were then resuspended in ‘complete activated media’: Neurobasal-A (Gibco, 10888-022) supplemented with 2% B27 minus vitamin A (Gibco, 12587-010), 1% penicillin–streptomycin–glutamine (Gibco, 10378-016), 20 ng ml^−1^ of EGF (PeproTech, AF-100-15) and 20 ng ml^−1^ of bFGF (PeproTech, 100-18B). For passaging, cells were dissociated with 1 ml Accutase (STEMCELL Technologies, 07920) for 5 min at 37 °C, washed once with 5 ml PBS, and resuspended in ‘complete activated media’ for expansion of aNSCs/NPCs (aNSCs/NPCs). For qNSCs, quiescence was induced over 5–10 d by replacing ‘complete activated media’ with ‘complete quiescent media’: Neurobasal-A (Gibco, 10888-022) supplemented with 2% B27 minus vitamin A (Gibco, 12587-010), 1% penicillin–streptomycin–glutamine (Gibco, 10378-016), 50 ng ml^−1^ of BMP4 (BioLegend 94073) and 20 ng ml^−1^ of bFGF (PeproTech, 100-18B). For adherent cultures of both qNSCs and aNSCs/NPCs, we coated plates with Poly-d-Lysine (PDL; Sigma-Aldrich, P6407, dilution of 1:20 in PBS) for 30–120 min at 37 °C, and washed plates 4× with PBS before plating cells at the appropriate density. All cell counting was performed using the Countess II FL Automated Cell Counter (Life Technologies, AMQAF1000).

### ATAC-seq library generation from primary neural stem cell cultures

To establish individual primary NSC cultures for ATAC-seq, we dissected and pooled the SVZs from one male and one female C57BL/6 NIA mouse from either a young cohort (3 months old) or an old cohort (23 months old). We cultured NSCs as described above (‘Primary neural stem cell culture’) to generate four young and four old independent cultures. At passage 5, NSCs were plated at a density of 1.2 million cells per 6 cm PDL-coated plate in complete quiescent media for 8 d before sorting. At passage 7, NSCs from the same culture were plated at a density of 1.5 million cells per 6-cm plate onto PDL-coated plates in complete activated media for 24 h before sorting to synchronize quiescent and activated NSC sorting experiments. Plates were washed 3× with PBS. Adherent qNSCs were lifted from the plate using 1 ml of Accutase (STEMCELL Technologies, 07920) incubated for 15 min at 37 °C and adherent aNSCs/NPCs were lifted from the plate using 1 ml of Accutase (STEMCELL Technologies, 07920) and incubated for 5 min at 37 °C. This cell suspension was diluted with 10 ml of PBS and spun down at 300*g* for 5 min. Pellet was resuspended in 200 μl of Neurobasal-A (Gibco, 10888-022) supplemented with 2% B27 minus vitamin A (Gibco, 12587-010), 1% penicillin–streptomycin–glutamine (Gibco, 10378-016) with propidium iodide (BioLegend, 421301; 1:5,000 dilution) for live/dead staining. Cells were kept on ice during all subsequent steps.

Due to concern about differing levels of dead cells in the cultures and the contaminating influence of dead cells on ATAC-seq libraries, all samples were sorted using FACS based on the live gate (propidium iodide). Around 10,000–15,000 live cultured qNSCs and aNSCs/NPCs were respectively sorted into 100 μl of NeuroBasal-A medium (Gibco, 10888-022) with penicillin–streptomycin–glutamine (Gibco, 10378-016) and 2% B27 minus vitamin A (Gibco, 12587-010) in a 96-well V-bottomed plate (Costar, 3894) and spun down at 300*g* for 5 min at 4 °C. Sorted cells were washed with 100 μl ice-cold PBS (Corning, 21-040-CV) and spun down at 300*g* for 5 min at 4 °C and processed as described in ‘ATAC-seq library generation from freshly isolated cells’ with the following exceptions. In total, 50 μl of transposition mix (12.5 μl 4x Tagment DNA (TD) buffer (gift from the Chang Lab), 35 μl nuclease-free H_2_O, 2.5 μl Tn5 (gift from the Chang Lab)) was used for each well instead of 5 μl. PCR amplification and subsequent qPCR monitoring was performed as previously described in the original ATAC-seq protocol^35^. All libraries were amplified for five PCR cycles and then an additional four PCR cycles (based off of qPCR amplification curves) and purified using the Zymo DNA Clean & Concentrator kit (Zymo, D4014) and eluted in 15 μl of nuclease-free H_2_O.

### Library sequencing and ATAC-seq quality control of in vivo and cultured neural stem cells

We quantified individual library concentrations using a Bioanalyzer (High Sensitivity) and pooled at a concentration of 5 nM for sequencing. Multiplexed libraries were sequenced using NextSeq (400 M) by the Stanford Functional Genomics Facility. To assess individual library quality, individual library paired-end FASTQ files were processed using the ATAC-seq pipeline from the laboratory of A.K. (https://github.com/kundajelab/atac_dnase_pipelines) with default parameters (using ‘--species mm10’ and including ‘--auto_detect_adapter’).

For in vivo ATAC-seq libraries generated from freshly isolated SVZ cells, libraries were excluded based on insufficient read coverage (<10 million unique reads) or low peak calling (≤20,000 peaks). In general, endothelial cell libraries were of worse quality than the other four sorted cell types and we additionally censored one endothelial library with low bowtie alignment (~92%) because all other libraries had a bowtie alignment of ≥95%. The high-quality libraries were sequenced to a mean read depth of 29,187,427 unique reads (ranging from ~10 to 69 million reads per library; Supplementary Table 1).

In general, ATAC-seq library quality was better for cultured NSCs than freshly isolated NSCs, so we used a different set of metrics for quality control. For cultured NSCs, one library (of four) from each condition was excluded due to poor quality, defined as the library with the lowest TSS enrichment (<20 in all cases). Additionally, both young and old quiescent cultures had one library that appeared highly anomalous (~twofold greater fraction of reads in peaks (FRiP) and TSS enrichment compared to every other library) so they were additionally excluded to avoid confounding results. The remaining 2–3 high-quality libraries per condition were sequenced to a mean read depth of 26,271,688 unique reads (ranging from ~18 to 42 million reads per library; Supplementary Table 1).

### ATAC-seq pipeline and processing

Libraries that passed quality control were reprocessed using the ATAC-seq pipeline from the laboratory of A.K. (https://github.com/kundajelab/atac_dnase_pipelines) starting from de-duplicated BAM files to call peaks per multi-replicate condition. De-duplicated, Tn5-shifted tagAlign files for each replicate were converted to BAM files (using bedToBam (v2.29.2)) and sorted (using samtools sort (v1.10)) for downstream analysis. Peaks per multi-replicate condition were selected using ‘overlap > optimal set’ resulting in approximately 20,000–90,000 peaks per in vivo condition (with a mean peakset size of 65,243 peaks) and approximately 90,000–150,000 peaks per cultured NSC condition (with a mean peakset size of 118,987 peaks). To generate pooled read libraries, the 2–3 high-quality filtered, de-duplicated BAM files for each condition were merged (using samtools merge (v1.10)), sorted (using samtools sort (v1.10)), Tn5-shifted (using deepTools alignmentSieve (v3.4.3)) and indexed (using samtools index (v1.10)). All analysis was performed using the *mm10* mouse genome (‘TxDb.Mmusculus.UCSC.mm10.knownGene’).

### Transcription start site enrichment

TSS enrichment heat maps were generated with ngsplot.R (v2.6.1) (ref. ^120^) using pooled, Tn5-shifted, sorted BAM files as inputs for each of the ten conditions.

### Generating consensus peaksets and count matrices

To generate consensus peaksets for downstream analysis, BAM files and multi-replicate peak files were loaded into a large DBA object using Diffbind (v2.10.0) (refs. ^121,122^) ‘dba.count’ with parameters ‘minOverlap = 0’ and ‘score = DBA_SCORE_READS’. We annotated peaks in the consensus count matrices using the ‘annotatePeak’ function of the package ChIPSeeker (v1.18.0) (ref. ^123^) with parameters ‘tssREgion = c(−3,000, 3,000)’.

### Functional enrichment of genetic elements within global peaksets

The ‘annotatePeak’ function of ChIPSeeker (v1.18.0) (ref. ^123^) was used to identify the genetic element identity of each chromatin peak within the multi-replicate peakset for each condition with parameters (tssRegion = c(−3,000, 3,000), annoDb = ‘org.Mm.eg.db’), and the annotation statistics were extracted using ‘@annoStat’. The different promoter terms ‘Promoter (≤1 kb)’, ‘Promoter (1–2 kb)’, and ‘Promoter (2–3 kb)’ were manually grouped together under ‘Promoter’, and the two intron terms were manually grouped under ‘Intron’.

### Principal component analysis

DESeq2 (v1.22.2) (ref. ^124^) was used to calculate dispersion estimates from raw consensus count matrices and then variance stabilizing transformations were applied before visualization by PCA.

For in vivo ATAC-seq libraries generated from freshly isolated SVZ cells, PCA on all chromatin peaks was generated using the global consensus peakset of 141,970 peaks. PCA consisting of all young and old qNSC and aNSC libraries was generated using the count matrix consisting of these 87,796 peaks. Based on peak annotations, the NSC consensus peakset was subdivided into a distal + intronic peakset (60,231 peaks), a distal peakset (31,660 peaks), an intronic peakset (28,571 peaks) and a promoter peakset (20,633 peaks). For ATAC-seq libraries generated from cultured NSCs, PCA was generated from the consensus peakset with 121,497 peaks. To determine how cultured NSCs compared to NSCs freshly isolated from the SVZ, we performed PCA on the count matrix consisting of the 11 freshly isolated NSC libraries and the 10 cultured NSC libraries (156,963 peaks).

### Principal component analysis with ATAC-seq peaks with enhancer marks

To identify ATAC-seq peaks that have enhancer marks, we downloaded FASTQ files for chromatin immunoprecipitation followed by sequencing (ChIP–seq) datasets for acetylated histone H3 Lys27 (H3K27ac) and p300—two marks of active enhancers—obtained from cultured qNSCs and proliferative (activated) NSCs^29^. We then processed these datasets using the standard ENCODE ChIP–seq pipelines. ATAC-seq peaksets that overlap with both H3K27ac and p300 marks were generated for qNSCs and aNSCs using ‘bedtools intersect --wa --u’ resulting in subsetted peaksets of 5,401 and 3,875, respectively. The resulting peak files were used to generate an accessibility count matrix (6,644 peaks), which was used for PCA as described above.

### Clustering ATAC-seq libraries from freshly isolated cells for heat map visualization

To cluster and visualize all ATAC-seq libraries from freshly isolated SVZ cell populations together, we generated a heat map from the global consensus peakset (141,970 peaks) with ‘cor()’ using the default Pearson’s correlation with the R library ‘pheatmap’ (v1.0.12).

### Bulk RNA-seq analysis of *Ascl1* mRNA expression

VST-normalized expression values of *Ascl1* mRNA were obtained from Leeman et al.^34^ and visualized for five SVZ cell populations.

### Correlating ATAC-seq promoter accessibility and single-cell RNA-seq expression

For the five cell populations freshly isolated from the SVZ of young and old mice for ATAC-seq, the average (VST-normalized) chromatin accessibility value of each gene’s promoter peak was associated with the average single-cell RNA-seq^22^ log-normalized expression value for that gene. Promoters were binned in deciles based on promoter accessibility in ATAC-seq, and the association between promoter chromatin accessibility and associated gene expression was plotted as box plots of deciles using R (v3.5.2).

### Chromatin signal track visualization for freshly isolated neural stem cells

Alignment tracks were visualized using IGV (v2.4.19). For each condition, the BAM file for a single representative library (Supplementary Table 1) was normalized by Reads per Kilobase per Million mapped reads (RPKM-normalization) and converted to a bigwig file using deepTools (v3.4.3) with the following parameters: ‘--extendReads 100 --normalizeUsing RPKM --binSize 10’.

### Differential peak calling with Diffbind

To identify differentially accessible peaks that change with age for each cell type, count matrices consisting of young and old replicates within a single cell type were generated using Diffbind (v2.10.0) (refs. ^121,122^) as described above (see ‘Generating consensus peaksets and count matrices’). Differential peak calling was accomplished using EdgeR (v3.24.3) (refs. ^125,126^) with the following parameters for ‘dba.analyze’: bCorPlot = FALSE, bParallel = TRUE, bTagwise = FALSE, bFullLibrarySize = TRUE, bReduceObjects = FALSE, method = DBA_EDGER’. For all comparisons, differential peaks were obtained using an FDR threshold of 0.05 (Supplementary Table 2). Differential peaks were annotated and associated with their closest gene using the ‘annotatePeak’ function of the package ChIPSeeker (v1.18.0) (ref. ^123^) with parameters ‘tssREgion = c(−3,000, 3,000)’. For the differential peaks that change with age in the freshly isolated qNSC and aNSC libraries, differential peaks were aligned to the *mm10* chromosomes using the ‘covplot()’ function in ChIPSeeker (v1.18.0) (ref. ^123^) for ease of visualization.

During this study, the Diffbind package was substantially updated leading to changes in differential peak calling (Diffbind v3). Using EdgeR with Diffbind v3, similar differential peaksets to those called by Diffbind v2 can be obtained with the settings ‘DBA$config$design <- FALSE’. We also tested another differential peak caller using Diffbind v3 (DESeq2), which could not call differential peaks in our samples at FDR < 0.05, possibly due to the low cell number used as input and the resulting relatively shallow sequencing depth. Due to this discrepancy, we verified that the original differential peaksets had clean signal pileups (Extended Data Fig. 3i; ‘Global signal pileup analysis of chromatin accessibility in neural stem cells’) and that the FDR values of the original differential peaksets (called by EdgeR using Diffbind v2) correlated well with the *P* values of DESeq2 peaks (using Diffbind v3; Extended Data Fig. 3l,m).

### Functional enrichment of genetic elements within differential neural stem cell peaksets

The differentially accessible peaks that change with age in the freshly isolated qNSC and aNSC conditions, were separated into sets that close with age or open with age and were annotated with the ‘annotatePeak’ function of ChIPSeeker (v1.18.0) (ref. ^123^) with parameters (tssRegion = c(−3,000, 3,000), annoDb = ‘org.Mm.eg.db’). The annotation statistics from ‘@annoStat’ were manually grouped into four categories: ‘distal intergenic’, ‘intron’, ‘promoter’ and ‘other’.

### Nucleosome peak calling

To identify whether the chromatin landscapes of young and old freshly isolated qNSCs and aNSCs had different levels of heterochromatin-associated nucleosomes, we used the package NucleoATAC (v0.2.1) (ref. ^127^). We called nucleosome peaks from our ATAC-seq data using ‘nucleoatac run’ with parameters: ‘--bed’ the multi-replicate peak files for each condition, ‘--bam’ pooled, downsampled (to 30 million unique reads), Tn5-shifted BAM files and ‘--fasta’ the mm10 *Mus musculus* UCSC genome. The number of nucleosome peaks for each condition were taken from the ‘*.nucmap_combined.bed’ files and plotted with R (v3.5.2).

### Global signal pileup analysis of chromatin accessibility in neural stem cells

We plotted the smoothed signal using the ‘fc.signal.bigwig’ tracks outputted by the ENCODE ATAC-seq pipeline for each of the tracks within differential peaks output by EdgeR for young and old qNSCs and aNSCs and subsampled common peaks for qNSC and aNSC separately.

### Gene Ontology biological pathway enrichment of differentially accessible chromatin peaks

Differentially accessible peaks were associated with nearby genes using the ChIPSeeker (v1.18.0) (ref. ^123^) function ‘annotatePeak’. Gene lists were uploaded to the online tool EnrichR^128,129^ and top ranked GO terms from ‘GO Biological Process 2018’ were extracted for pathways that change with age (Supplementary Table 3). Selected GO terms were ranked by *P* value (calculated using Fisher’s exact test) and plotted for visualization in R (v3.5.2).

### Gene Ontology biological pathway enrichment of genes driving principal component axes

To identify what biological processes were associated with peaks driving the PCs in the PCA of young and old freshly isolated qNSCs and aNSCs, the top 1,000 peaks driving the principal components (either negatively or positively) were extracted and associated with genes (Supplementary Table 4). The genes (under header ‘symbol’) associated with these peaks were uploaded to EnrichR to identify top ranked GO terms from ‘GO Biological Process 2018’ (Supplementary Table 5). The top six GO terms for PC2 negative peaks (grouping old qNSCs and young aNSCs) and PC2 positive peaks (grouping young qNSCs and old aNSCs) were ranked by *P* value and plotted in R (v3.5.2) for visualization.

### Chromatin peak heat maps within cell adhesion pathways

To visualize how aging affects chromatin peak accessibility associated with cell adhesion and migration, genes associated with differential peaks that change with age in either freshly isolated qNSCs or aNSCs were intersected with the ‘cell adhesion’ GO gene list (GO:0007155) or the ‘negative regulation of cell migration’ GO gene list (GO:0030336; http://www.informatics.jax.org/). Differential peak accessibility levels were plotted as heat maps using ‘pheatmap’ (v1.0.12) in R (v3.5.2; trimmed mean of M values (TMM)-normalized read counts, scaled row-wise).

### Venn diagrams of cell adhesion genes with dynamically accessible chromatin peaks

To visualize whether the opposing chromatin changes during aging in qNSCs and aNSCs involved shared adhesion genes, we used Venn diagrams to visualize the number of cell adhesion genes (from the ‘cell adhesion’ GO gene list (GO:0007155)) with nearby chromatin peaks (annotated by ChIPSeeker (v1.18.0) (ref. ^123^)) that were differentially open in young qNSCs (compared to old qNSCs) and open in old aNSCs (compared young aNSCs). We also used a Venn diagram to visualize the overlap of cell adhesion genes with nearby chromatin peaks that open with age in both aNSCs and NPCs.

### De novo transcription factor binding patterns using a deep learning model and TF-MoDISco

To identify transcription factor binding patterns, ATAC-seq peaks for in vivo quiescent or activated NSCs isolated from young and old mice were separately converted to BigWig tracks of base-resolution Tn5 insertion sites with a +4/−4 shift to account for Tn5 shift. For each cell type, in addition to the peak regions, we selected an equal number of non-peak regions that were matched for GC content in their peaks. We then trained cell-type-specific BPNet models to predict the log counts and base-resolution Tn5 insertion profiles as previously reported^130,131^. Briefly, the BPNet model takes as input a 2,114-bp one-hot encoded input sequence and predicts the ATAC-seq profile and log counts in a 1,000-bp window centered at the input sequence. Following BPNet formulation, we used a multinomial negative log likelihood for the profile output of the model and a mean square error loss for the log counts output of the model. The relative loss weight used for the counts loss was 0.1 times the mean total counts per region. During each epoch, training examples were jittered by up to 500 bp on either side and a random half of the sequences were reverse complemented. Each batch contained a 10:1 ratio of peaks to non-peak regions. Model training was performed using Keras/Tensorflow 2. Code used for model training is available at https://github.com/kundajelab/retina-models/.

We computed importance scores for the counts output using the DeepSHAP^132^ implementation of DeepLIFT algorithm^133^. We next ran the TF-MoDISco algorithm^134^ to perform de novo motif discovery in each cell type. We mapped the discovered motifs to position weight matrices from HOCOMOCO^135^ using TomTom^136^ (v5.5.2). For each cell type, we computed the relative fraction of TF-MoDISco seqlets attributed to each motif (Extended Data Fig. 8a).

### Single-cell RNA-seq expression values for cell adhesion and migration pathways

Single-cell RNA-seq datasets from young and old SVZ neurogenic niches^22^, consisting of 14,685 cells (8,884 from young and 5,801 from old), were used to identify how gene expression of specific molecular signatures and genes involved in cell adhesion and migration differs by cell type and age at single-cell resolution.

Cell types were clustered and annotated as described previously^22^. In brief, we performed *t*-distributed stochastic neighbor embedding clustering using Seurat^137^ with the first 15 PCs and performed PCA on the 4,125 most variable genes. Significant clusters and marker genes for each cluster were found using the Seurat functions FindClusters() and FindAllMarkers(). This identified 11 different cell types—qNSCs/astrocytes (which cluster together), aNSCs/NPCs (which cluster together), neuroblasts, neurons, oligodendrocytes, progenitor cells, oligodendrocytes, endothelial cells, mural cells, microglia, macrophages and T cells), which were annotated using a combination of marker genes identified from the literature and GO for cell types using EnrichR (http://amp.pharm.mssm.edu/Enrichr/). Single-cell gene expression values for three cell types—qNSCs/astrocytes (which cluster together), aNSCs/NPCs (which cluster together) and neuroblasts—were extracted and subset based on genes from GO lists of cell adhesion and migration pathways. For each single cell within a cell population, the expression levels of cell adhesion/migration genes were summed. The cumulative expression level of adhesion genes comparing different cell types was visualized using violin plots with R (v3.5.2). Statistical comparisons between conditions were performed in R using a two-tailed Mann–Whitney test. We also performed a Welch-corrected *t*-test and results were similar. A two-tailed Mann–Whitney test was chosen for reporting because it does not require data to be normally distributed and is commonly used to make comparisons for single gene expression values^138,139^.

### Differentially accessible chromatin peaks of specific cell adhesion genes that change with age

For specific ‘cell adhesion’ genes (GO:007155; *Alcam*, *Ctnnd2*, *Itgb8*, *Lsamp* and *Ntm*), TMM-normalized accessibility values for differential peaks that change with age were obtained from EdgeR (Diffbind v2; Supplementary Table 2) and visualized using box plots with R (v4.1.0). When there were multiple chromatin peaks associated with the same gene that change with age, the peak upstream of and closest to the TSS was chosen.

### Single-cell RNA-seq expression values for specific cell adhesion genes

Using the same single-cell RNA-seq dataset as above^22^, we used the FindMarkers function from Seurat (v4.0.5) and tested if specific cell adhesion genes (*Alcam*, *Ctnnd2*, *Itgb8*, *Lsamp*, *Ntm*) whose chromatin changed with age in qNSCs and aNSCs also showed transcriptional changes. Statistical comparisons between conditions were performed in R using a two-tailed Mann–Whitney test. We validated these differentially expressed genes in two other single-cell RNA-seq datasets of SVZ neurogenic niches from mice at different ages^41,140^. We picked three representative genes that were significantly downregulated with age in qNSCs/astrocytes in at least two of the three single-cell RNA-seq datasets and three representative genes that were significantly upregulated with age in aNSCs/NPCs in at least two of the three single-cell RNA-seq datasets. Expression values were log-normalized counts per 10,000 transcripts and were visualized using violin plots with R (v3.5.2). Data shown are from same dataset^22^ described above.

### Gene expression trajectory of specific cell adhesion genes during aging

Gene expression trajectories as a function of age were derived from single-cell gene expression data of the SVZ of 28 mice, tiling ages from young to old (3.3 months to 29 months)^41^. Expression values were log-normalized counts per 10,000 transcripts. Each dot represents the gene expression value per mouse. The shaded region corresponds to the 95% confidence interval. The line is a smoothed fit using geom_smooth with method = ’loess’ (ggplot2 v3.3.5).

### Assessing cell cycle heterogeneity and cell adhesion pathways using single-cell RNA-seq

Using single-cell RNA-seq dataset from young and old SVZ neurogenic niches^22^, we investigated the heterogeneity in cell cycle and cell adhesion pathways (Extended Data Fig. 6). Using Seurat package (v3.1.5) (ref. ^137^), single cells were assigned to different cell cycle stages (G0/G1, G2/M and S phase) and assigned a continuous ‘G2/M score’ and ‘S-phase score’ based on the expression levels of G2/M phase marker genes and S-phase marker genes, respectively, by the Seurat CellCycleScoring() function. A low ‘S-phase score’ indicates that cells express low levels of S-phase marker genes. The proportion of single cells from young and old SVZ neurogenic niches that belong to these three different cell cycle stages was then plotted as bar plots.

We plotted the relationship between Seurat’s ‘S-phase score’ and the cumulative expression of genes from the ‘cell adhesion’ GO gene list (GO:0007155) in young and old qNSCs/astrocytes, aNSCs/NPCs and neuroblasts. For this analysis, each young cell population was downsampled to the number of old cells (480 qNSCs/astrocytes, 82 aNSCs/NPCs and 146 neuroblasts). Some aNSCs became more ‘quiescent like’ with age (Extended Data Fig. 6b,c). However, adhesion and proliferation could also be uncoupled, as some old aNSCs exhibit adhesion changes without exhibiting proliferation changes (Extended Data Fig. 6c).

### Immunofluorescence staining of young and old primary neural stem cells

NSCs were cultured as described above (‘Primary neural stem cell culture’) in complete activated media until passages 2–4 and then passaged with Accutase (STEMCELL Technologies, 07920) and seeded at a density of 5,000 cells per well in Matrigel-coated coverslips (Corning, 354230, 0062012; 1:100 dilution in cold DMEM/F12; Thermo Fisher Scientific, 11320033) in 24-well plates with complete activated or quiescent media. After 48 h, adherent aNSCs/NPCs were washed ×1 with PBS, then fixed with 4% paraformaldehyde (PFA; Electron Microscopy Sciences, 15714) for 15 min at room temperature. Wells were washed ×4 with PBS and then stored in PBS, wrapped in parafilm, at 4 °C until immunofluorescence staining. For qNSCs, quiescent medium was replaced every other day for 7 d and then adherent qNSCs were fixed with 4% PFA (Electron Microscopy Sciences, 15714) for 15 min at room temperature, washed ×4 with PBS, and then stored in PBS, wrapped in parafilm, at 4 °C until immunofluorescence staining. We found that primary NSCs that had undergone freeze-thaw cycles had diminished age-related differences and thus used fresh dissections for all experiments.

For immunostaining, cells were permeabilized with 0.1% Triton X-100 (Fisher Scientific, BP151) for 10 min at room temperature, and then washed 2× with PBS. Blocking was performed for 30 min with 1% BSA (Sigma, A7979) in PBS. Primary staining was conducted for 1–1.5 h at room temperature with phalloidin (Invitrogen, A12379, 665217; 1:500 dilution), ALCAM/CD166 (Bio-techne, AF1172-SP; 1:40 dilution), PXN (Abcam, ab32084; 1:200 dilution) or cleaved caspase3 (Cell Signaling Technology, 9664T; 1:1,000 dilution) resuspended in 1% BSA (Sigma, A7979). Wells were then washed ×4 with PBS and secondary staining was performed for 1 h at room temperature with Donkey anti-Rabbit Alexa 568 (Invitrogen, A10042; 1:1,000 dilution) or Donkey anti-Goat Alexa 647 (Invitrogen, A21447; 1:1000 dilution) resuspended in 1% BSA (Sigma, A7979). DAPI (Thermo Fisher, 62248; 1:500 dilution) was added during secondary antibody staining. Wells were washed ×4 with PBS + 0.2% TWEEN 20 (Sigma-Aldrich, P1379-1L), then ×4 with PBS. Coverslips were mounted onto glass slides with ProLong Gold Antifade Mountant with DAPI (Thermo Fisher, P36931) and visualized with a Nikon Eclipse Ti confocal microscope equipped with a Zyla sCMOS camera (Andor) and NIS-Elements software (AR 4.30.02, 64-bit). Quantification of immunofluorescence staining of ALCAM was done using a custom pipeline in Fiji (v2) (ref. ^141^). The phalloidin channel was used to create a cell mask and overlaid onto the ALCAM channel. The sum of pixel intensity within the cell mask (RawIntDen) was then used to determine fluorescence intensity for each cell, normalized by cell size. For each experiment, values were normalized by dividing each cell’s fluorescence intensity by the mean fluorescence intensity of all cells in the young condition for aNSCs/NPCs and the old condition for qNSCs, and the same threshold settings were used to create the phalloidin cell mask for all images within one experiment. For focal adhesion quantification, the PXN channel was used. Images were converted to 8-bit and background subtracted (rolling = 5 sliding), threshold was set (kept the same for all images taken within one experiment), a region of interest (ROI) was drawn around cell periphery, and number of focal adhesions counted (‘analyze particles’, size = 0.5–infinity). For cleaved caspase3 quantification, CellProfiler (v4.2.1) was used to identify and count nuclei (DAPI) using the IdentifyPrimaryObject function and cleaved caspase3-positive cells using the IdentifySecondaryObjects function. Staining, imaging and analysis were performed in a blinded manner. *P* values were calculated with a two-tailed Mann–Whitney test comparing sample means. For immunofluorescence images (Fig. 2h,k and Extended Data Fig. 7e), brightness and contrast were adjusted in Fiji (v2) to enhance visualization. These adjustments were performed after all data quantification was complete. The same settings were applied to all images shown for each experiment.

### Quantitative FACS of cultured primary neural stem cells

NSCs were cultured as described above (‘Primary neural stem cell culture’) in complete activated media until passages 5–6 and then passaged with Accutase (STEMCELL Technologies, 07920) and seeded at a density of 200,000 cells per well in Matrigel-coated (Corning, 354230, 0062012; 1:100 dilution in cold DMEM/F12; Thermo Fisher Scientific, 11320033) six-well plates (Falcon, 353046) with complete activated media (for aNSCs/NPCs) or quiescent media (for qNSCs). For aNSCs/NPCs, cells were processed 24 h after plating. For qNSCs, cells were processed after 7 d in quiescent media (replaced every other day). For subsequent steps, qNSCs and aNSCs/NPCs were processed in the same manner. Media were removed and 1 ml of Accutase (STEMCELL Technologies, 07920) was added to each well and incubated at 37 °C for 5 min. Cells were triturated repeatedly with a P1000 to dissociate and lift cells and then transferred to a 15-ml conical tube pre-filled with 1 ml PBS, and 1 ml of PBS was added to each well to recover any remaining cells. All subsequent steps were performed on ice or at 4 °C. Cells were centrifuged at 300*g* for 5 min at 4 °C. Samples were decanted, and pellet was resuspended in 150 μl PBS + 2% FBS (Gibco, 10099-141) and transferred to a 96-well U-bottomed plate (Falcon, 353077). This plate was then centrifuged at 400*g* for 5 min at 4 °C. After centrifugation, the plate was immediately inverted to remove supernatant and stained with ALCAM-APC (R&D, FAB1172A, AASL0320111; 10 μl per 10^6^ cells) resuspended in PBS + 2% FBS (Gibco, 10099-141) at 4 °C for 30 min while shaking. After incubation, samples were washed with PBS + 2% FBS (Gibco, 10099-141) and centrifuged at 400*g* for 5 min at 4 °C. Next, the plate was immediately inverted, and cells were resuspended in 200 μl PBS + 2% FBS (Gibco, 10099-141) + DAPI (Thermo Fisher, 62248; 1:500 dilution) and filtered through a cell strainer snap cap (Falcon, 352235). Unstained, single-stained and fluorescence-minus-one controls were also prepared. Cells were immediately run on a BD LSR II, alternating between young and old conditions to minimize batch effect. For each sample, 10,000 live cells were recorded. FlowJo (v10.7.1) was used to export the scaled fluorescence values for ALCAM from all live cells. Fluorescence values were then normalized to the young mean for each independent experiment. *P* values were calculated using a two-tailed Mann–Whitney test comparing sample means.

### Detachment assay of cultured quiescent neural stem cells and activated neural stem cells/neural progenitor cells

For the enzyme-based cell detachment assay, aNSCs/NPCs were cultured in complete activated media until passages 2–6 and then seeded at a density of 30,000 cells per well in PDL-coated 96-well plates (Falcon, 353072) with complete activated media. The next day, medium on adherent aNSCs/NPCs was replaced with complete activated media containing 10 nM Syto64 (Invitrogen, S11346, 8344573) to visualize the cells and incubated for 1–2 h. Medium was replaced with complete activated medium to remove excess Syto64. The plates were immediately imaged with the ×4 objective for whole-well imaging with the red image channel on the Incucyte S3 system. Each well was visually inspected for appropriate cell density, no cell clumping and even distribution of cells. After imaging, medium was removed and replaced with 100 μl of Accutase (STEMCELL Technologies, 07920) and incubated at room temperature for 5 min to lift cells. Accutase (STEMCELL Technologies, 07920) was aspirated and wells were washed once with PBS and 100 μl of complete activated media was added to the plate. A multichannel pipette was used to minimize pipetting differences between conditions. Cells were also plated such that young and old conditions were paired and would experience similar mechanical force during pipetting. The plates were immediately imaged with Incucyte S3 ×4 objective for whole-well imaging with the red image channel. For analysis, well quantification area was restricted by 250 pixels using Incucyte’s built-in analysis software to decrease noise from cell debris accumulating at the edges of the well. Cell count was calculated using Incucyte’s built-in analysis software and the percentage of cells remaining was calculated as 100 × (number of cells after Accutase/number of cells before Accutase).

For the enzyme-based cell detachment assay on qNSCs, aNSCs/NPCs were seeded at a density of 10,000 cells per well in PDL-coated 96-well plates with complete quiescent medium to induce quiescence. Complete quiescent medium was replaced every other day for 7 d to induce quiescence. Next, qNSCs were incubated with Syto64, imaged, and analyzed as described above for aNSCs/NPCs with the following change: qNSCs were treated with Trypsin-EDTA (referred to as trypsin in the paper; 0.25%) phenol red (Thermo Fisher Scientific, 25200114; instead of Accutase) for 15 min at room temperature to lift the cells. Trypsin, a stronger dissociation agent, was used instead of Accutase because qNSCs are much more adhesive than aNSCs/NPCs and Accutase could not effectively detach cells (Extended Data Fig. 7n). *P* values were calculated using a two-tailed Mann–Whitney test on the average of 2–4 technical replicates (wells) for each independent culture (from an individual mouse). Experiments were not performed in a blinded manner; however, quantification of the percentage of cells remaining was done in an automated manner using Incucyte’s analysis software for cell counting. For all enzyme-based cell detachment assays, a multichannel pipette was used to ensure equal pipetting force was applied to young and old samples.

For the centrifugation-based cell detachment assay^52,53^, aNSCs/NPCs were cultured and plated in a 96-well plate (Falcon, 353072) as described above for the enzyme-based assay. Cells were plated toward the center of the plate (that is, between columns 5–9 of a 96-well plate) to minimize variation in centrifugal force as force is a function of distance to the rotor. After imaging the plate on the Incucyte S3 system with the ×4 objective for whole-well imaging, 210 μl of warm completed activated media (final volume in well is 310 μl) was added to each well to fill the well and then sealed with Microseal ‘B’ PCR Plate Sealing Film (Bio-Rad, MSB1001). We used 210 μl of additional complete activated media to minimize the air gap in each well. Adding more than 210 μl of media resulted in a poor seal with the sealing film, causing media to leak during centrifugation. After the plate was sealed, it was inverted and centrifuged at 300*g* for 4 min at room temperature. After centrifugation, the sealing film was removed and medium was gently aspirated to remove any cells that had detached. Next, 100 μl of warm complete activated medium was gently added to each well with a multichannel pipette and immediately imaged with the Incucyte S3 with a ×4 objective for whole-well imaging. Images were cropped to a circle of radius 700 pixels in CellProfiler (v4.2.1) to decrease noise from cell debris accumulating at edges of well. Adhesion surface was calculated as the percentage area covered by cells using CellProfiler (v4.2.1) with the IdentifyPrimaryObjects function and outputting area occupied by objects. The percentage of remaining area was calculated as 100 × (amount before spinning/amount after spinning). *P* values were calculated using a two-tailed Mann–Whitney test on the average of 2–3 technical replicates (wells) for each independent culture from an individual mouse. Experiments were not performed in a blinded manner; however, quantification of the percentage of cells remaining was performed in an automated manner using CellProfiler (v4.2.1).

### NFIC CRISPR–Cas9 gene knockout in young and old cultured activated neural stem cells/neural progenitor cells

#### Selection of gene to knockout

We used the following criteria to select a transcription factor to knock out in aNSCs/NPCs: (1) Identified in chromatin accessibility to change with age in aNSCs, (2) expressed in aNSCs (using single-cell RNA-seq), (3) known to be implicated in cell adhesion and migration. The NFI family of transcription factors (NFIA, NFIB, NFIC and NFIX) met these criteria. We focused on NFIC because our pilot studies targeting this isoform were the most promising.

#### Cloning of sgRNAs

To knockout NFIC, we used a CRISPR–Cas9 approach, using aNSCs/NPCs microdissected from SVZs of young and old Cas9-expressing mice (Rosa26-Cas9^2^) and delivered guide RNAs to these cells using lentivirus. We used the sgRNA-expressing plasmid MCB320 (gift from the laboratory of M. Bassik; https://www.addgene.org/89359/) and subcloned sgRNAs of interest. This plasmid contains a puromycin-resistance gene and mCherry reporter for selection. See Supplementary Table 7 for all primers used for cloning and sequencing. The MCB320 plasmid was digested with Blp1 and BstXI restriction enzymes and the gel-extracted, purified band was used for ligation reaction with a double-stranded oligonucleotide containing the sgRNA sequence. For the forward oligonucleotide of each sgRNA sequence, we added cloning adaptor sequences: 5′-ttgg and 3′-gtttaagagc. For the reverse complement oligonucleotide of each sgRNA sequence, we used the reverse complement of the sgRNA sequence and added 5′-ttagctcttaaac and 3′-ccaacaag. Each pair of oligonucleotides (IDT, standard desalting; 1 μM each of forward and reverse) was annealed in 98 μl of nuclease-free duplex buffer (IDT, 11-05-03-01) for 5 min at 95 °C and then gradually cooled to room temperature. Then, 1 μl of a 1:20 dilution of the annealed oligonucleotide (in nuclease-free duplex buffer (IDT, 11-05-03-01)) was ligated with 500 ng of the purified digested plasmid band and transformed in NEB stable competent cells (New England BioLabs, C3050H). Successful ligation of the guide was confirmed by Sanger sequencing using a mU6 sequencing primer (Supplementary Table 7).

#### Lentivirus production

For lentivirus production, human embryonic kidney 293T cells (HEK293T/17, ATCC, CRL-11268) were cultured in ‘complete DMEM’: (Gibco, 11965092) + 10% FBS (Gibco, 10099-141) + 1% penicillin–streptomycin–glutamine (Gibco, 10378-016; complete DMEM). HEK293T cells were plated at a density of 5 million cells per 10 cm plate (Greiner Bio-One, 664-160). At 16 h after plating, medium was replaced with fresh complete DMEM, and 3–4 h later, HEK293T cells were transfected using polyethylenimine (PEI; Polysciences, 23966-2; 1 mg ml^−1^) and 3rd generation lentivirus packaging vectors (pMDLg, pRSV and pVSVG; gift from the laboratory of M. Bassik). Per transfection, 50 μl of PEI was added to 900 μl of antibiotic and FBS-free DMEM (Gibco, 11965092) and incubated at room temperature for 10 min. In total, 0.5 μg of each packaging vector (pMDLg, pRSV and pVSVG) was combined with 10 μg of MCB320 lentiviral plasmid containing guide of interest into a final volume of 50 μl. This DNA mix was then added to the DMEM–PEI mixture and incubated at room temperature for 20 min. The PEI–DNA mixture was then added dropwise to HEK293T cells, and 24 h later, the medium was replaced with plain Neurobasal-A (Gibco, 10888-022; no growth factors or B27). Supernatant (10 ml) was collected 24 h later and the medium was replaced for another collection 48 h after transfection. Supernatants were combined, centrifuged at 3,000*g* for 5 min, filtered through a 0.45-μm filter (EMD Millipore, SE1M003M00), and then flash frozen into 1.5-ml aliquots and stored at −80 °C until ready for use.

#### Infection of activated neural stem cells/neural progenitor cells

Activated NSCs/NPCs were cultured from young and old mixed-sex Rosa26-Cas9^2^ knock-in mice as described in ‘Primary neural stem cell culture’. One million aNSCs/NPCs per well were plated onto PDL-coated 6-well plates in 1 ml of complete activated media. At 24 h later, medium was removed and cells were infected by adding 0.75 ml of filtered viral supernatant combined with 0.25 ml of complete activated medium, supplemented with 4× growth factors and B27 (final volume of growth factors and B27 is 1×) and added to adherent aNSCs/NPCs. After 24 h, infection was repeated. After 24 h, medium was changed to complete activated media. Two days later, successful infection was verified by microscopy using the mCherry reporter. Once mCherry expression was confirmed, 0.5 μg ml^−1^ of puromycin (Sigma-Aldrich, P8833) was added to the culture media to select for cells that had been successfully infected. When all uninfected control aNSCs/NPCs died from puromycin selection (approximately 2–3 d later), adherent aNSCs/NPCs were washed with complete activated media to remove puromycin and allowed to expand for 24-48 h. aNSCs/NPCs were then passaged and plated onto PDL-coated 96-well plates for detachment assay. All remaining cells (usually 250,000–400,000) were plated onto PDL-coated 24-well plates for genomic DNA collection for verification of the knockout (see below). If there were not enough cells to perform the detachment assay and genomic DNA collection, cells were expanded as neurospheres for an additional 2–3 d. If cultures failed to recover from lentiviral transduction (continued slow growth), they were not used for subsequent experiments. At 16–24 h after plating cells, we performed the detachment assay as described above (‘Detachment assay of cultured quiescent neural stem cells and activated neural stem cells/neural progenitor cells’) except cells were incubated with Accutase for 3 min instead of 5 min. All experiments were performed in a paired manner (paired young and old). *P* values were calculated using a Wilcoxon matched-pairs signed-rank test comparing sample means. To account for the paired-manner of the experiment, the difference in the percentage of cells remaining (old aNSCs/NPCs − young aNSCs/NPCs) was also calculated. Difference in the percentage of cells remaining greater than 0 indicates old cells are more adhesive than young cells. *P* values comparing the difference in the percentage of cells remaining were calculated using a two-tailed Mann–Whitney test.

#### Verification of knockout efficacy

For genomic DNA isolation, aNSCs/NPCs were plated at a density of 250,000–400,000 cells per well of a PDL-coated 24-well plate at the same time as cells were plated for the detachment assay. After 16–24 h, genomic DNA was harvested (same day as detachment assay). Cells were washed with 500 μl cold PBS and then lysed with 100 μl DirectPCR Lysis Reagent (Viagen Biotech, 102-T) with 1% Proteinase K (Fisher Scientific, 25-530-049) for 10 min at room temperature. Supernatant was pipetted repeatedly to lift cells and then transferred to PCR tubes and incubated at 65 °C for 25 min and then 95 °C for 15 min in a thermocycler. PCR was performed to amplify the region targeted by sgRNA and Sanger sequenced (see primers in Supplementary Table 7). Knockout efficiency was evaluated using the Synthego ICE analysis tool (v3.0; https://ice.synthego.com/#/) (ref. ^142^). With this tool, a score of 60 indicates 60% of the population sequenced has a frameshift mutation or deletion greater than 21 base pairs.

### Migration assay of cultured quiescent neural stem cells and activated neural stem cells/neural progenitor cells using live-cell imaging

aNSCs/NPCs were cultured in complete activated media until passages 2–4 and then seeded at a density of 200,000 cells per well in a PDL-coated 12-well plate with complete activated media treated with vehicle (H_2_O) or 10 μM Y-27632 (dissolved in H_2_O; Tocris, 1254). After 48 h, adherent aNSCs/NPCs were passaged with 500 μl of Accutase (STEMCELL Technologies, 07920), and resuspended in in Neurobasal-A (Gibco, 10888-022) supplemented with 2% B27 minus vitamin A (Gibco, 12587-010) and 1% penicillin–streptomycin–glutamine (Gibco, 10378-016) with propidium iodide (BioLegend, 421301; 1:5,000 dilution) for live/dead staining. A total of 1,000 live (PI^−^) cells per well were sorted with flow cytometry using the BD PICI onto PDL-coated Incucyte ImageLock 96-well plates (Essen BioScience, 4379) containing 100 μl of complete activated media with 10 nM Syto64 (Invitrogen, S11346, 8344573) and either vehicle (H_2_O) or 10 μM Y-27632 (dissolved in H_2_O; Tocris, 1254). The ImageLock plate was immediately brought to the Incucyte Zoom or Incucyte S3 live imaging system to image (with phase and red image channels) at 37 °C every 30 min for 20 h with the ×20 objective. After 20 h, media was washed ×2 with PBS and then replaced with 100 μl of complete quiescent media. Medium was changed every other day for 7 d to induce quiescence. After induction, quiescent medium was supplemented with 10 nM Syto64 (Invitrogen, S11346, 8344573) before imaging. Wells were imaged (with phase and red image channels) using either the Incucyte S3 or Incucyte Zoom system with the ×20 objective every hour for 20 h at 37 °C. Three images (from different parts of the well) at each time point were taken for each well and tracked cell metrics were summed across these three images. This 20-h window includes time needed for cells to adhere to the plate (early time points) in addition to migration (later time points). Cell migration tracking was performed in an automated manner using Imaris (v9.3.0). Every image stack was manually inspected to ensure that cell tracking was correctly performed (for example, that automated cell tracking was not incorrectly labeling debris and that cell tracks accurately follow individual cells). ‘Track Velocity’ was output for visualization with Prism (v8) and *P* values were calculated with a two-tailed Mann–Whitney test comparing both single-cell values as well as sample mean values (where single-cell velocities were averaged by animal). Experiments were not performed in a blinded manner; however, quantification of cell migration velocity was performed in an automated manner using Imaris (v9.3.0).

### Matrigel dispersion assay

For Matrigel dispersion assays, aNSCs/NPCs were cultured in complete activated media until passages 4–6, passaged with Accutase (STEMCELL Technologies, 07920) and resuspended in complete activated media at a concentration of 100,000 cells per microliter. Matrigel (Corning, 354230, no. 8344573 for experiments with young and old aNSCs/NPCs in Fig. 3 and no. 9028255 for experiments with ROCKi in Fig. 7) was diluted at a 1:2 ratio in cold F12:DMEM (Gibco, 11-330-057) while on ice to prevent solidification, and 50 μl was immediately added per well to a 96-well plate. We note that there are lot-to-lot variations in the quantity of extracellular matrix proteins present in Matrigel, which likely affect activated the ability of NSCs to disperse through the material. The 96-well plate was moved to the 37 °C incubator for 3 h before spotting 50,000 cells (in a volume of 0.5 μl) directly to the center of the Matrigel-coated well. The plate was returned to the 37 °C incubator for 15–30 min to allow for cells to adhere. Next, 150 μl of complete activated media (treated with vehicle (H_2_O) or 10 μM Y-27632 (dissolved in H_2_O; Tocris, 1254)) was carefully added to each well (for the ROCKi experiments), and the 96-well plate was placed in either the Incucyte Zoom or Incucyte S3 live imaging system to image every 6 h for 48 h using the ×4 objective to image the entire well. For each independent culture, 1–4 technical replicates (wells) were quantified to account for difficulties in spotting 0.5 μl volumes, and the distances migrated at each time point were averaged among technical replicates. We manually quantified dispersion distance, defined as the maximum distance from the initial spotting perimeter to the outermost cell body, using Fiji (v2) (ref. ^141^). Neither imaging nor analysis was performed in a blinded manner. *P* values were calculated with a two-tailed Mann–Whitney test at each 12-h time point. At early time points, cell proliferation/survival are less likely to be strong contributing factors in the Matrigel assay than at later time points.

### In vitro EdU pulse assay of cultured activated neural stem cells/neural progenitor cells

Cultured aNSCs/NPCs from young and old male C57BL/6 mice were plated onto PDL-coated coverslips in 24-well plates with complete activated media with or without 10 μM ROCKi compound Y-27632 (dissolved in H_2_O; Tocris, 1254). At 48 h after plating, 10 μM of EdU (Thermo Fisher Scientific, C10086) was added to adherent aNSCs/NPCs. Two hours later, aNSCs/NPCs were washed once with PBS and then fixed with 4% PFA and processed for immunostaining as previously described (‘Immunofluorescence staining of young and old primary neural stem cells’), except Click-iT EdU Alexa Fluor 488 imaging protocol (Thermo Fisher Scientific, C10337) was performed, according to the manufacturer’s instruction, for 30 min at room temperature before blocking. Samples were imaged at ×20 with a Nikon Eclipse Ti confocal microscope equipped with a Zyla sCMOS camera (Andor) and NIS-Elements software (AR 4.30.02, 64-bit). CellProfiler (v4.2.1) was used to identify and count nuclei (DAPI) using the IdentifyPrimaryObjects function and EdU^+^ nuclei using the IdentifySecondaryObjects function. Staining, imaging and analysis were performed in a blinded manner. *P* values were calculated with a two-tailed Mann–Whitney test comparing sample means.

### Immunofluorescence staining of subventricular zone coronal sections to measure distance of quiescent and activated neural stem cells relative to the ventricle

For immunofluorescence staining of SVZ coronal sections to measure the distance of quiescent and activated NSCs relative to the ventricle, four young (4 months old) and four old (18–26 months old) GFAP-GFP animals of both sexes (two male and two female animals per age condition), were subjected to intracardiac perfusion with 4% PFA (Electron Microscopy Sciences, 15714) in PBS. Brains were post-fixed overnight in 4% PFA (Electron Microscopy Sciences, 15714) and then dehydrated in 30% sucrose (Sigma-Aldrich, S3929) for 72 h. Brains were embedded in Tissue-Tek optimal cutting temperature compound (Electron Microscopy Sciences, 62550), sectioned in 16-μm coronal sections using a cryostat (Leica, CM3050S), and then mounted on electrostatic glass slides (Fisher Scientific, 12-550-15). For these experiments, six serial coronal sections (corresponding to images 42–47 from the Allen Institute Coronal Brain Atlas; https://mouse.brain-map.org/static/atlas) were selected for immunostaining. The three anterior serial coronal sections were stained with antibodies to GFAP, Ki67, vinculin, and with DAPI and the three more posterior serial coronal sections were stained with antibodies to S100a6, Ki67, ARL13b, and with DAPI (see below).

Coronal sections were washed with PBS and then permeabilized with ice-cold methanol + 0.1% Triton X-100 (Fisher Scientific, BP151) for 15 min at room temperature. Slides were washed 3× with PBS for 5 min. Antigen retrieval was performed in 10 mM sodium citrate buffer (pH 6.0; 2.94 g Tri-sodium citrate dihydrate (Sigma-Aldrich, S1804) in 1,000 ml milliQ H_2_O adjusted to pH 6.0 with 1 N HCl) + 0.05% TWEEN 20 (Sigma-Aldrich, P1379-1L) in a 85 °C water bath for 2 h. Slides were then cooled to room temperature for 20 min (in antigen retrieval buffer) and coronal sections were blocked with 5% normal donkey serum (NDS; ImmunoReagents, SP-072-VX10) and 1% BSA (Sigma, A7979) in PBS for 30 min at room temperature. Primary antibody staining was performed overnight at 4 °C in 5% NDS and 1% BSA in PBS. Primary antibodies used were: Ki67 (Invitrogen, clone SolA15, 14-5698-82; 1:200 dilution), GFAP (Abcam, ab53554; 1:500 dilution), vinculin (Abcam, ab129002; 1:200 dilution), S100a6 (Abcam, ab181975; 1:500 dilution) and ARL13b (Abcam, ab136648; 1:500 dilution). Vinculin (cell adhesion protein) and ARL13b (ciliary marker) were used to demarcate the ventricle border, for GFAP staining and S100a6 staining, respectively. Different antibodies were used to mark the ventricular wall because of species compatibility for the staining panel. Coronal sections were then washed 3× with PBS and 0.2% TWEEN 20 for 10 min at room temperature followed by 2× PBS washes for 15 min. Secondary antibody staining was performed at room temperature for 2 h in 5% NDS and 1% BSA in PBS. Secondary antibodies used were the following: Donkey anti-Goat 488 (Sigma-Aldrich, SAB460032-250UL; 1:1,000 dilution), Donkey anti-Rat 594 (Life Technologies, A21209; 1:1,000 dilution), Donkey anti-Mouse 488 (Life Technologies, A21202; 1:1000 dilution), Donkey anti-Goat 488 (Invitrogen, A11055; 1:1,000 dilution), Donkey anti-Rabbit 568 (Invitrogen, A10042; 1:1,000 dilution) and/or Donkey anti-Rat 647 (Invitrogen, A48272; 1:1,000 dilution). DAPI (Thermo Fisher, 62248; 1:500 dilution) was added during secondary antibody staining. Sections were washed 3× with PBS and 0.2% TWEEN 20 for 10 min at room temperature followed by 3× PBS washes for 5 min. Coronal sections were mounted with ProLong Gold Antifade Mountant with DAPI (Thermo Fisher, P36931). Multiple *z*-stacks using the ×60 objective of a Nikon Eclipse Ti confocal microscope equipped with a Zyla sCMOS camera (Andor) and NIS-Elements software (AR 4.30.02, 64-bit) were captured to image the entire length of the SVZ from one hemisphere per section (three sections per animal for GFAP staining and three sections per animal for S100a6 staining). For immunofluorescence images (Fig. 4c and Extended Data Fig. 9a,b), brightness and contrast were adjusted in Fiji (v2) to enhance visualization. These adjustments were performed after all data quantification was complete and the same settings were applied to all images shown for each experiment.

### Quantification of the location of quiescent and activated neural stem cells with respect to the ventricle in coronal sections

For quantification of the location of quiescent and activated NSCs with respect to the ventricle in coronal sections, *z*-stacks (30 slices per stack for GFAP staining and 15 slices per stack for S100a6 staining) were transformed into a *z*-projection (sum slices). Analysis was restricted to 200 μm from the ventricle wall to not include striatal astrocytes (although the vast majority of cells were less than 50 μm away from the ventricle wall; Fig. 4c and Extended Data Fig. 9a,b).

GFAP and S100a6 were used as NSC markers. For staining with GFAP, GFAP^+^/Ki67^−^ cells were identified as qNSCs/astrocytes and GFAP^+^/Ki67^+^ cells were identified as aNSCs. For staining with S100a6, S100a6^+^/Ki67^−^ cells were identified as qNSCs and S100a6^+^/Ki67^+^ cells were identified as aNSCs. NSCs directly lining blood vessels were censored in the analysis because they were considered to be localized to a blood vessel rather than the niche lining the ventricle wall. Distances from the ventricle were calculated by measuring the Euclidean distance from the center of the nucleus of each cell of interest to the ventricle border in Fiji (v2). Neither imaging nor cell-type annotation was performed in a blinded manner; however, distance quantification was performed in an automated manner. *P* values were calculated with a two-tailed Mann–Whitney test comparing sample means.

### Immunofluorescence staining of subventricular zone sagittal sections for distance of quiescent and activated neural stem cells and neuroblasts relative to the ventricle

For immunofluorescence staining of SVZ sagittal sections for the distance of quiescent and activated NSCs and neuroblasts relative to the ventricle, five young male (2–3 months) and five old male (21 months) C57BL/6 mice were used (combined over two independent experiments; note that these animals were the same as those used in the 4-h time point in ‘In vivo EdU labeling to follow location of cells in subventricular zone, rostral migratory stream and olfactory bulb’, see below). Mice were intraperitoneally injected with EdU (Fisher Scientific, A10044; resuspended in PBS at 5 mg ml^−1^) at a dose of 50 mg per kg body weight 4 h before intracardiac perfusion with 4% PFA (Electron Microscopy Sciences, 15714) in PBS.

Brains were processed and immunostained in a similar manner as coronal sections (‘Immunofluorescence staining of subventricular zone coronal sections to measure distance of quiescent and activated neural stem cells relative to the ventricle’), except they were sectioned in 16-μm sagittal sections rather than coronal. Sagittal sections corresponding approximately to image 14 from the Allen Institute Sagittal Brain Atlas (https://mouse.brain-map.org/static/atlas/) were stained and imaged as described in ‘Immunofluorescence staining of subventricular zone coronal sections to measure distance of quiescent and activated neural stem cells relative to the ventricle’ with the antibodies described below.

For immunostaining for qNSCs/astrocytes and activated NSCs in sagittal sections, primary antibodies used were: Ki67 (Invitrogen, clone SolA15, 14-5698-82; 1:200 dilution), GFAP (Abcam, ab53554; 1:500 dilution) and vinculin (Abcam, ab129002; 1:200 dilution). Secondary antibodies used were: Donkey anti-Goat 488 (Sigma-Aldrich, SAB460032-250U; 1:1,000 dilution), Donkey anti-Rabbit 568 (Invitrogen, A10042; 1:1,000 dilution) and Donkey anti-Rat 647 (Invitrogen, A48272; 1:1,000 dilution). DAPI (Thermo Fisher, 62248; 1:500 dilution) was added during secondary antibody staining.

For immunostaining for EdU^+^ aNSCs/NPCs and neuroblasts, the Click-iT EdU Alexa Fluor 488 imaging protocol (Thermo Fisher Scientific, C10337) was performed for 30 min at room temperature before blocking, according to the manufacturer’s instructions. The following primary antibodies were used: Ki67 (Invitrogen, clone SolA15, 14-5698-82; 1:200 dilution) and DCX (Cell Signaling Technologies, 4604; 1:500 dilution). The following secondary antibodies were used: Donkey anti-Rabbit Alexa 568 (Invitrogen, A10042; 1:1,000 dilution) and Goat anti-Rat Alexa 647 (Invitrogen, A21247; 1:1,000 dilution). DAPI (1 mg ml^−1^; Thermo Fisher, 62248) was included at a concentration of 1:500 with the secondary antibody mix.

Multiple images tiling the entire length of the ventricle from one sagittal section per animal were captured with a ×20 objective using a Zeiss LSM 900 confocal microscope equipped with Zeiss ZEN Blue 3.0 software. Images were stitched together using the Zeiss ZEN Blue 3.0 software to create a single image containing the entire SVZ. For immunofluorescence images (Fig. 4f), brightness and contrast were adjusted in Fiji (v2) to enhance visualization. These adjustments were performed after all data quantification was complete and the same settings were applied to all images shown for each experiment.

### Quantification of the location of quiescent and activated neural stem cells and neuroblasts with respect to the ventricle in sagittal sections

For quantification of the location of quiescent and activated NSCs and EdU^+^ aNSCs/NPCs and EdU^+^ neuroblasts with respect to the ventricle in sagittal sections, one section containing the entire RMS was used for quantification to ensure consistency in the region being analyzed from animal to animal. Analysis of the SVZ was restricted to the region that is 200 μm from the ventricle wall to not include striatal astrocytes (although the vast majority of cells were less than 50 μm away from the ventricle wall; Fig. 4f).

In the staining panel with DAPI, GFAP, Ki67 and vinculin, GFAP^+^/Ki67^−^ cells were considered qNSCs/astrocytes and GFAP^+^/Ki67^+^ cells were considered aNSCs. In the staining panel with DAPI, EdU, Ki67 and DCX, EdU^+^ cells were classified as aNSCs/NPCs if they were Ki67^+^/DCX^−^ and as neuroblasts if they were DCX^+^ (and either Ki67^+^ or Ki67^−^). Quantification and analysis of the distance of qNSCs/astrocytes, aNSCs, EdU^+^ aNSCs/NPCs and EdU^+^ neuroblasts to the ventricle was performed as described above (‘Quantification of the location of quiescent and activated neural stem cells with respect to the ventricle in coronal sections’).

### Single-cell RNA-seq to detect potential ependymal-repairing subventricular zone astrocytes and reactive astrocytes

#### Ependymal-repairing subventricular zone astrocytes

SVZ astrocytes that intercalate into the ependymal lining are GFAP^+^ and proliferative and can take on ependymal markers^17,63^. To test if old aNSC populations could contain ependymal-repairing astrocytes, we performed UMAP analysis on cells from the SVZ neurogenic niche on single-cell gene expression data of 21,458 single-cell transcriptomes of cells^41^ from the SVZ neurogenic niches of 28 mice, tiling ages from young to old using Seurat (v4.0.5) (ref. ^137^). We filtered for cells expressing markers of ependymal-repairing SVZ astrocytes (*Gfap*, *S100b*, *Cd24a* and *Ctnnb1*) (refs. ^17,63^) using FeaturePlot (min.cutoff = ‘q10’, max.cutoff = ‘q90’; Seurat v4.0.5). This identified four cells that expressed these four markers. All four of these cells clustered with ependymal cells. The presence of these cells did not appear to be dependent on old age, as these four cells were from animals that were 4.7, 5.4, 16.83 and 18.87 months old. We also examined the presence of ependymal-repairing astrocytes in our other single-cell RNA-seq dataset^22^ (which has very few ependymal cells), and we observed two cells exhibiting markers of ependymal-repairing SVZ astrocytes (of 14,685 cells), one from young and one from old animals. These data suggest that ependymal-repairing SVZ astrocytes are present but not numerous in the SVZ neurogenic niche.

#### Reactive astrocytes

To test if any cells in our single-cell datasets of the SVZ neurogenic niche (described above) expressed markers of reactive astrocytes, we performed a similar UMAP analysis. We used markers of A1 reactive astrocytes (produced in response to neuroinflammation)^67^, A2 reactive astrocytes (produced in response to ischemia)^67^ and pan-injury reactive astrocytes (identified from meta-analysis of 15 transcriptomic reactive astrocyte datasets^143^; see Supplementary Table 8 for full list of gene markers). We did not detect any cell in either dataset (of 21,458 cells in one dataset^41^ and 14,685 cells in another dataset^22^) that expressed these types of reactive astrocyte markers^67,143^.

### In vivo EdU labeling to follow location of cells in subventricular zone, rostral migratory stream and olfactory bulb

For these experiments, 12 young (2–3 months) and 12 old (21 months) male C57BL/6 animals (combined over two independent experiments) were intraperitoneally injected with EdU (Fisher Scientific, A10044; resuspended in PBS at 5 mg ml^−1^) at a dose of 50 mg per kg body weight 4 h, 2 d or 7 d before intracardiac perfusion with 4% PFA (Electron Microscopy Sciences, 15714) in PBS. The five young (2–3 months) and five old (21 months) male C57BL/6 mice that were intraperitoneally injected with EdU 4 h before intracardiac perfusion used here were the same as the animals used above in ‘Immunofluorescence staining of subventricular zone sagittal sections for distance of quiescent and activated neural stem cells and neuroblasts relative to the ventricle’ and ‘Quantification of the location of quiescent and activated neural stem cells and neuroblasts with respect to the ventricle in sagittal sections’. Brains were processed and immunofluorescence staining of sagittal sections was performed as described above (‘Immunofluorescence staining of subventricular zone sagittal sections for distance of quiescent and activated neural stem cells and neuroblasts relative to the ventricle’).

Multiple sections per brain, corresponding to image 14 from the Allen Institute Sagittal Brain Atlas (https://mouse.brain-map.org/static/atlas/) were stained and imaged. Sagittal sections were selected for quantification based on presence of an intact RMS as visualized by a stream of DCX-positive cells connecting the SVZ to the OB. Very few sections contained a fully intact RMS and one young 7-d post-injection replicate from the second experiment was censored due to lack of high-quality sections at the appropriate depth. Images of sagittal sections were captured using a ×5 objective, and 9 × 3 images (for a total of 27) were stitched together using the Zeiss ZEN Blue 3.0 software. For quantification, EdU-labeled nuclei were counted using Fiji (v2) (ref. ^141^). Every image was converted to 8-bit, the threshold was adjusted ((90,255) for replicate 1 and (70,255) for replicate 2), watershed was applied to the image and EdU-labeled nuclei were counted in an automated fashion with ‘analyze particles (size (micron^2): (20-infinity) for replicate 1 and 2, and circularity: (0.2–1.00) for replicate 1)’. EdU-labeled nuclei were quantified in three regions (along the entire length of the ventricle for SVZ, the entire length of the RMS and the entire OB), which were manually defined using a hand-drawn ROI for each section. Neither imaging nor analysis was performed in a blinded manner; however, cell counting was performed in an automated manner with the same thresholding for all images for each replicate. *P* values were calculated with a two-tailed Mann–Whitney test. For immunofluorescence images (Fig. 5b,c and Extended Data Fig. 9j), brightness and contrast were adjusted in Fiji (v2) to enhance visualization. These adjustments were performed after all data quantification was complete and the same settings were applied to all images shown for each experiment.

### Quantification of efficiency of EdU incorporation

We used the same sagittal section images that were quantified above for EdU localization to SVZ, RMS and OB (‘In vivo EdU labeling to follow location of cells in subventricular zone, rostral migratory stream and olfactory bulb’). To calculate EdU labeling efficiency, we analyzed the number of EdU-labeled nuclei in the SVZ 4 h after EdU injection as described above. The number of Ki67^+^ cells was quantified in the same region using the same pipeline as counting EdU-labeled nuclei. The same threshold was applied to all images captured the same day. To calculate EdU labeling efficiency 4 h after EdU injection, the number of EdU^+^ cells was divided by the total number of Ki67^+^ cells in the SVZ in each image. Neither imaging nor analysis was performed in a blinded manner; however, cell counting was performed in an automated manner with the same thresholding for all images for each replicate. *P* values were calculated with a two-tailed Mann–Whitney test.

### Ingenuity pathway analysis

Genes associated with differentially accessible peaks (FDR < 0.05) that open in old aNSCs freshly isolated from the SVZ were uploaded to IPA (v1.16) (ref. ^72^) (QIAGEN; https://www.qiagenbioinformatics.com/products/ingenuitypathway-analysis/) to identify age-related regulatory changes (Supplementary Table 6). For each peak-associated gene, we uploaded the log fold-change, *P* value and FDR (*q* value) and based the IPA analysis on FDR. Statistical enrichment of pathways was reported with *P* values calculated by IPA using right-tailed Fisher’s exact test.

### RGD molecular tension sensor FRET measurements of cultured neural stem cells

Sensors containing a minimal RGD sequence derived from fibronectin (TVYAVTGRGDSPASSAA) were expressed, purified and labeled with Alexa Fluor 546 and Alexa Fluor 647, and coverslips passivated with maleimide polyethylene glycol (PEG) succinimidyl carboxymethyl ester (JenKem Technology, A5003-1) were prepared as previously described^52,144^. Flow chambers for imaging were prepared as described^144^ with slight modifications. Eight-well flow chambers (Grace BioLabs, 622505; ~55 µl channel volume) were attached to PEGylated coverslips as previously described^145^. Chambers were incubated with 300 nM double-labeled sensor at room temperature for 45 min. The chambers were then washed with 150 µl of PBS for 1 min to prevent nonspecific cell attachment. Around 60 µl of cell suspensions at a density of 300,000–400,000 cells per ml were added to the channels, and the chambers were incubated for 3 h at 37 °C and 5% CO_2_ in complete activation media to allow time for cells to spread. Chambers were then washed with 150 µl of warm media, and brightfield and FRET measurements were made immediately after and acquired with an objective heater (Bioptechs) set to 37 °C. For measurements on cells treated with the ROCKi Y-27632, complete activated medium was supplemented with 10 μM Y-27632 (dissolved in H_2_O; Tocris, 1254) for cell suspensions and media washes.

We limited our measurements and analysis to individual cells that looked well-spread in brightfield and cell clusters of no more than five cells in which cell outlines could be clearly distinguished. Under conditions used, the vast majority of cells fulfilled these criteria. FRET fluorescence measurements were performed with objective-type total internal reflection fluorescence (TIRF) microscopy on an inverted microscope (Nikon TiE) with an Apo TIRF ×100 oil objective lens, numerical aperture 1.49 (Nikon) as described previously^52^ and controlled using Micromanager^146^. Samples were excited with 532-nm (Crystalaser) or 635-nm (Blue Sky Research) lasers. Emitted light passed through a quad-edge laser-flat dichroic with center/bandwidths of 405/60 nm, 488/100 nm, 532/100 nm and 635/100 nm from Semrock (Di01-R405/488/532/635-25×36) and corresponding quad-pass filter with center/bandwidths of 446/37 nm, 510/20 nm, 581/70 nm and 703/88 nm band-pass filter (FF01-446/510/581/703-25). Donor and acceptor images were taken through separate additional cubes stacked into the light path (donor: 550 nm long-pass; acceptor: 679/41 nm and 700/75 nm) and recorded on a Hamamatsu Orca Flash 4.0 camera.

Images were prepared in Fiji (v2) (ref. ^141^) and analyzed using custom MATLAB scripts, in which FRET efficiencies were computed and thresholded to identify adhesions and quantify forces within adhesions. Adhesions were identified based on FRET values measured using the RGD tension sensor. FRET efficiencies were computed for each pixel in each image, and regions exceeding specific FRET efficiencies and area cutoffs were identified as adhesions using a Watershed algorithm. Forces at adhesions were substantially greater than any signal beneath the cell body, which is similar to background, consistent with previous force measurements at focal adhesions^52^ (Extended Data Fig. 10c,d).

### Immunofluorescence staining of cultured neural stem cells treated with ROCK inhibitor

aNSCs/NPCs were cultured and plated as described above (‘Immunofluorescence staining of young and old primary neural stem cells’) with complete activated or quiescent media with or without 10 μM Y-27632 (dissolved in H_2_O; Tocris, 1254). After 48 h, adherent aNSCs/NPCs (with or without 10 μM Y-27632 (dissolved in H_2_O; Tocris, 1254)) were fixed with 4% PFA as described above (‘Immunofluorescence staining of young and old primary neural stem cells’). For qNSCs, quiescent medium (with or without 10 μM Y-27632) was replaced every other day for 7 d and then fixed with 4% PFA as described above. Staining and quantification for ALCAM, focal adhesions (PXN), cleaved caspase3 and DCX was performed in the same manner as described above (‘Immunofluorescence staining of young and old primary neural stem cells’) except quantification for DCX^+^ cells was performed using CellProfiler (v4.2.1). The function IdentifyPrimaryObjects was used to identify and count nuclei (DAPI) that served as a seed for IdentifySecondaryObjects to identify and count cells that were DCX positive. Antibodies used were phalloidin (Invitrogen, A12379, 665217; 1:500 dilution), ALCAM/CD166 (Bio-techne, AF1172-SP; 1:40 dilution), PXN (Abcam, ab32084; 1:200 dilution) and DCX (Cell Signaling Technologies, 4604; 1:500 dilution) resuspended in 1% BSA (Sigma, A7979). Staining, imaging and analysis were performed in a blinded manner. *P* values were calculated with a two-tailed Mann–Whitney test comparing sample means. For immunofluorescence images (Fig. 6h), brightness and contrast were adjusted in Fiji (v2) to enhance visualization. These adjustments were performed after all data quantification was complete. The same settings were applied to all images shown for each experiment.

### Osmotic pump surgery for in vivo intracerebroventricular delivery of ROCK inhibitor in old mice

For delivery of ROCK inhibitor in the lateral ventricle of old mice, Y-27632 (ROCKi; Tocris, 1254) was diluted in artificial cerebrospinal fluid (Tocris, 3525) to a final concentration of 100 μM. A concentration of 100 μM was chosen based on a previous study^79^. Osmotic pumps (ALZET/Durect, model 2002) with a 14-d infusion rate of 0.5 μl per hour were loaded with 200 μl of diluted Y-27632 or vehicle control (artificial cerebrospinal fluid only) and allowed to equilibrate in PBS in a 37 °C water bath overnight. Mice were anesthetized with isoflurane and received postsurgical buprenorphine and saline. Osmotic pumps were connected to a cannula (ALZET, Brain infusion kit III) and inserted by stereotactic surgery at +1 mm lateral, −0.3 mm anterior–posterior and −3 mm deep relative to bregma to target the right lateral ventricle of old (20–22 months old) male C57BL/6 mice (8–10 mice per condition (vehicle control or ROCKi), combined over two independent experiments). The pump connected to the cannula was then placed subcutaneously along the back of the mouse. Block randomization was used on cages of mice such that an equal number of mice per cage were assigned to each experimental group. The day before surgery, mice were singly housed and cage mates were separated across experimental conditions. We alternated performing the surgery between control and Y-27632 mice to avoid batch effects.

One week after start of drug delivery, mice were intraperitoneally injected with EdU (Fisher Scientific, A10044) (resuspended in PBS at 5 mg ml^−1^) at a dose of 50 mg per kg body weight either 4 h or 7 d before intracardiac perfusion with 4% PFA (Electron Microscopy Sciences, 15714) in PBS.

### Quantification of distance of neural stem cells relative to ventricle and of location of EdU^+^ cells in different locations in old mice treated with ROCK inhibitor

Quantification of the sagittal distance of aNSCs/NPCs was performed as described above in ‘Quantification of the location of quiescent and activated neural stem cells and neuroblasts with respect to the ventricle in sagittal sections’, except that all Ki67^+^/DCX^−^ cells were counted as aNSCs/NPCs (rather than filtering first by cells that are EdU^+^) and images were captured with the ×60 objective of a Nikon Eclipse Ti confocal microscope equipped with a Zyla sCMOS camera (Andor) and NIS-Elements software (AR 4.30.02, 64-bit). *P* values were calculated with a two-tailed Mann–Whitney test comparing sample means.

For quantification of the number of EdU cells in the entire SVZ (along the entire ventricle), entire length of the RMS and entire OB, sagittal sections were immunostained, imaged and analyzed as described above in ‘In vivo EdU labeling to follow location of cells in subventricular zone, rostral migratory stream and olfactory bulb’ except analysis of EdU^+^ cell localization (in SVZ, RMS or OB) was performed in a blinded manner.

Brain sections adjacent to the ones used for quantification of the number of EdU cells in the entire SVZ, entire RMS and entire OB were immunostained as described above in ‘Immunofluorescence staining of subventricular zone sagittal sections for distance of quiescent and activated neural stem cells and neuroblasts relative to the ventricle’. The following primary antibodies were used: DCX (Cell Signaling Technologies, 4604; 1:500 dilution) and NeuN (Millipore, clone A60, MAB377, 2919670; 1:500 dilution). The following secondary antibodies were used: Donkey anti-Rabbit Alexa 647 (Invitrogen, A31573; 1:1,000 dilution) and Donkey anti-Mouse Alexa 568 (Invitrogen, A10037; 1:1,000 dilution). DAPI (1 mg ml^−1^; Thermo Fisher, 62248) was included at a concentration of 1:500 with the secondary antibody mix. Images of EdU^+^ cells in the OB were captured with the ×60 objective of a Nikon Eclipse Ti confocal microscope equipped with a Zyla sCMOS camera (Andor) and NIS-Elements software (AR 4.30.02, 64-bit). For immunofluorescence images (Fig. 8b,e,i), brightness and contrast were adjusted in Fiji (v2) to enhance visualization. These adjustments were performed after all data quantification was complete and the same settings were applied to all images shown for each experiment.

### Statistics and reproducibility

For all experiments that were not blinded, young and old conditions were processed in an alternate manner rather than in two large groups, to minimize the group effect. Mice from multiple orders from the NIA or multiple cages were used to control for covariates and experiments were performed alternating between experimental groups (either young–old or control–treatment) to avoid batch effect. For in vivo EdU experiments and in vivo ROCKi experiments, block randomization was used on cages of mice such that an equal number of mice per cage were assigned to each experimental group. We did not perform power analyses, although we did take into account previous experiments to determine the number of animals needed. To calculate statistical significance for experiments, all tests were two-sided Mann–Whitney tests unless otherwise stated. Blinding was generally not performed except for when noted in Methods. However, all quantification (with the exception of Matrigel dispersion assays and cell-type annotation for immunohistochemistry experiments) were performed in an automated fashion using software tools. We have indicated that no blinding was performed when relevant in the Methods.

For the young-versus-old Matrigel dispersion experiment, three young replicates were excluded due to poor cell viability (tenfold less recovered cells compared to other young and old conditions). For the young-versus-old in vivo EdU-mediated migration experiment, one young (7 d) and one old (7 d) animal were excluded because there was no EdU labeling due to failed intraperitoneal injections. For ATAC-seq on freshly isolated and cultured NSCs, low-quality ATAC-seq libraries were excluded based on quality-control metrics (Methods). For the in vivo ROCKi experiment, two animals that received no treatment (4 h), three animals that received no treatment (7 d) and two ROCK inhibitor-treated (7 d) animals were excluded due to failed intraperitoneal injection (no EdU-labeled) or mass brain bleeding during surgery. An additional two animals that received no treatment (4 h), one ROCKi-treated animal (4 h), two animals that received no treatment (7 d) and one ROCKi-treated animal (7 d) were excluded for SVZ quantification due to tearing of brain sections in these regions preventing quantification. All other data were included in the study.

### Reporting summary

Further information on research design is available in the Nature Portfolio Reporting Summary linked to this article.

## Supplementary information

Reporting Summary

Supplementary Table 1 Number of animals and cells sorted to generate each ATAC-seq library and associated quality-control metrics for ATAC-seq libraries (initial read counts, final read counts, bowtie alignment, mitochondrial read percentage, final read percentage Picard complexity, peaks called, TSS enrichment, fraction of reads in DNAseq hypersensitive sites (DHSs), fraction of reads in peaks).

Supplementary Table 2 Differentially accessible ATAC-seq peaks that change with age in freshly isolated cells from the SVZ neurogenic niche (endothelial cells, astrocytes, qNSCs, aNSCs and NPCs) and in culture (qNSCs and aNSCs/NPCs).

Supplementary Table 3 GO Biological Processes enrichment of genes with nearby differentially accessible peaks that change with age in freshly isolated cells from the SVZ neurogenic niche (endothelial cells, astrocytes, qNSCs, aNSCs and NPCs) and in culture (qNSCs and aNSCs/NPCs).

Supplementary Table 4 List of top 1,000 genes driving PC1, PC2 and PC3 from PCA on freshly isolated young and old qNSCs and aNSCs.

Supplementary Table 5 GO Biological Processes associated with the top 1,000 genes driving PC1, PC2 and PC3 from PCA on freshly isolated young and old qNSCs and aNSCs.

Supplementary Table 6 Canonical Pathway enrichment using IPA of genes associated with differentially accessible chromatin peaks that open with age in old aNSCs freshly isolated from the SVZ neurogenic niche.

Supplementary Table 7 Primer sequences used for cloning of lentiviral CRISPR–Cas9 plasmids and Synthego inference of CRISPR edits (ICE) analysis.

Supplementary Table 8 Markers used to assess for presence of reactive and ependymal-repairing astrocytes, assembled from literature.

Supplementary Video 1 Live-cell imaging of old (20–24 months) cultured aNSCs/NPCs migrating on a PDL-coated plate over 20 h. Cultured aNSCs/NPCs are in the presence of Syto64, a cell-permeant red fluorescent nucleic acid stain, to enable live-cell migration tracking.

Supplementary Video 2 Live-cell imaging of old (20–24 months) cultured aNSCs/NPCs treated with 10 μM ROCKi migrating on a PDL-coated plate over 20 h. Cultured aNSCs/NPCs are in the presence of Syto64, a cell-permeant red fluorescent nucleic acid stain, to enable live-cell migration tracking.

## Data Availability

All raw sequencing data for ATAC-seq libraries can be found under BioProject PRJNA715736. Raw and processed single-cell RNA-seq data were retrieved from BioProject PRJNA450425 (ref. ^22^), BioProject PRJNA795276 (ref. ^67^) and https://zenodo.org/record/7145399#.ZFKWsezMJ6o (ref. ^67^). Raw H3K27ac and p300 ChIP–seq data were retrieved from the European Nucleotide Archive under accession number ERP002084. Gene annotation was based on the mm10 mouse genome (TxDb.Mmusculus.UCSC.mm10.knownGene). Source data are provided with this paper.

## Source data

Source Data Fig. 1 Statistical source data.
Source Data Fig. 2 Statistical source data.
Source Data Fig. 3 Statistical source data.
Source Data Fig. 4 Statistical source data.
Source Data Fig. 5 Statistical source data.
Source Data Fig. 6 Statistical source data.
Source Data Fig. 7 Statistical source data.
Source Data Fig. 8 Statistical source data.
Source Data Extended Data Fig. 1 Statistical source data.
Source Data Extended Data Fig. 7 Statistical source data.
Source Data Extended Data Fig. 8 Statistical source data.
Source Data Extended Data Fig. 9 Statistical source data.
Source Data Extended Data Fig. 10 Statistical source data.

## Code availability

The code used to analyze genomic data is available in the GitHub repository for this paper (https://github.com/brunetlab/Yeo_RW_NSC_ATACseq/). Code used to process raw ATAC-seq data is available at https://github.com/kundajelab/atac_dnase_pipelines/. Code used for deep learning model training for transcription factor binding site identification is available at https://github.com/kundajelab/retina-models/.
