# Supplementary material for: Chromatin accessibility dynamics of neurogenic niche cells reveal defects in neural stem cell adhesion and migration during aging
Source: Nat Aging. 2023 Jul 13;3(7):866–93. doi: 10.1038/s43587-023-00449-3 (PMC10353944; doi:10.1038/s43587-023-00449-3)
Supplement: Supplementary file 1 — Reporting Summary [file 43587_2023_449_MOESM1_ESM.pdf]

nature portfolio

Corresponding author(s): Dr. Anne Brunet

Last updated by author(s): May 22, 2023

Reporting Summary

Nature Portfolio wishes to improve the reproducibility of the work that we publish. This form provides structure for consistency and transparency in reporting. For further information on Nature Portfolio policies, see our Editorial Policies and the Editorial Policy Checklist.

Statistics

For all statistical analyses, confirm that the following items are present in the figure legend, table legend, main text, or Methods section.

n/a	Confirmed	
☐	☒	The exact sample size (*n*) for each experimental group/condition, given as a discrete number and unit of measurement
☐	☒	A statement on whether measurements were taken from distinct samples or whether the same sample was measured repeatedly
☐	☒	The statistical test(s) used AND whether they are one- or two-sided<br>*Only common tests should be described solely by name; describe more complex techniques in the Methods section.*
☐	☒	A description of all covariates tested
☐	☒	A description of any assumptions or corrections, such as tests of normality and adjustment for multiple comparisons
☐	☒	A full description of the statistical parameters including central tendency (e.g. means) or other basic estimates (e.g. regression coefficient) AND variation (e.g. standard deviation) or associated estimates of uncertainty (e.g. confidence intervals)
☐	☒	For null hypothesis testing, the test statistic (e.g. *F*, *t*, *r*) with confidence intervals, effect sizes, degrees of freedom and *P* value noted<br>*Give P values as exact values whenever suitable.*
☒	☐	For Bayesian analysis, information on the choice of priors and Markov chain Monte Carlo settings
☒	☐	For hierarchical and complex designs, identification of the appropriate level for tests and full reporting of outcomes
☐	☒	Estimates of effect sizes (e.g. Cohen's *d*, Pearson's *r*), indicating how they were calculated

Our web collection on statistics for biologists contains articles on many of the points above.

Software and code

Policy information about availability of computer code

Data collection	NIS-Elements software (version AR 4.30.02), BD FACSDiva software (version 8.0.1), Zen blue edition (version 3.0), Incucyte S3, Incucyte ZOOM
Data analysis	Data analysis was performed using R (v3.5.2, v4,1,0). Packages include: samtools (v1.10), bedtools (v2.29.2), deeptools (v3.4.3), ngsplot (v2.6.1), Diffbind (v2.10.0, v3), ChIPSeeker (v1.18.0), DESeq2 (v1.22.2), pheatmap (v1.0.12), EdgeR (v3.24.3), NucleoATAC (v0.2.1), Seurat (v3.1.5, v4.0.5), ggplot2 (v3.3.5)<br><br>Other analysis tools used include Imaris (v9.3.0), Prism (v8), Ingenuity Pathway Analysis (IPA) (v1.16) (https://www.qiagenbioinformatics.com/products/ingenuitypathway-analysis), Fiji (v2), EnrichR (https://maayanlab.cloud/Enrichr/), Flowjo (v8, v10.7.1), CellProfiler (v4.2.1), Synthego ICE (v3.0)(https://ice.synthego.com/#/), Incucyte (v2021B), IGV (v2.4.19), TomTom (v5.5.2), BD FACSDiva (v8.0.1).<br><br>The code used to analyze genomic data in the current study is available in the Github repository for this paper (https://github.com/brunetlab/Yeo_RW_NSC_ATACseq). Code used to process ATAC-seq data is available at https://github.com/kundajelab/atac_dnase_pipelines. Code used for deep learning model training for transcription factor binding site identification is available at https://github.com/kundajelab/retina-models.

For manuscripts utilizing custom algorithms or software that are central to the research but not yet described in published literature, software must be made available to editors and reviewers. We strongly encourage code deposition in a community repository (e.g. GitHub). See the Nature Portfolio guidelines for submitting code & software for further information.

Data

Policy information about availability of data

All manuscripts must include a data availability statement. This statement should provide the following information, where applicable:

- Accession codes, unique identifiers, or web links for publicly available datasets
- A description of any restrictions on data availability
- For clinical datasets or third party data, please ensure that the statement adheres to our policy

All raw sequencing data for ATAC-seq libraries can be found under BioProject PRJNA715736. Raw and processed single-cell RNA-seq data were retrieved from BioProject PRJNA45042522, BioProject PRJNA795276 41, and https://zenodo.org/record/7145399#.ZFKWsezMJ6o. Raw H3K27ac and p300 ChIP-seq data were retrieved from the European Nucleotide Archive under accession number ERP002084. Gene annotation was based on the mm10 mouse genome (TxDb.Mmusculus.UCSC.mm10.knownGene). Figures 1-2 and Extended Data Figures 1, 2, 3, 4, 7, and 8 of this study are based on the raw data which can be found under this accession number. For both figures, raw sequences for ATAC-seq libraries are provided as FASTQ files.

Human research participants

Policy information about studies involving human research participants and Sex and Gender in Research.

Reporting on sex and gender	n/a
Population characteristics	n/a
Recruitment	n/a
Ethics oversight	n/a

Note that full information on the approval of the study protocol must also be provided in the manuscript.

Field-specific reporting

Please select the one below that is the best fit for your research. If you are not sure, read the appropriate sections before making your selection.

☒ Life sciences ☐ Behavioural & social sciences ☐ Ecological, evolutionary & environmental sciences

For a reference copy of the document with all sections, see nature.com/documents/nr-reporting-summary-flat.pdf

Life sciences study design

All studies must disclose on these points even when the disclosure is negative.

Sample size	Sample size was decided based on previous experiments and prior literature (Leeman et al 2018; Mahmoudi et al 2019; Dulken et al 2019) using similar experimental paradigms. We did not use power analyses and have clearly indicated this in Materials and Methods (Statistics section). We have clearly indicated cases where samples from independent experiments were combined, and have included all combined and non-combined data (and associated statistics) in Source Data.
Data exclusions	For the young vs old matrigel dispersion experiment, 3 young replicates were excluded due to poor cell viability (10-fold less recovered cells compared to other young and old conditions).<br>For the young vs old in vivo EdU-mediated migration experiment, one young (7 day) and one old (7 day) animals were excluded since there was no EdU-labeling due to failed intraperitoneal injections.<br>For ATAC-seq on freshly isolated and cultured NSCs, low-quality ATAC-seq libraries were excluded based on QC metrics (described in Methods).<br>For the in vivo ROCK inhibitor experiment, 2 no treatment (4 hour), 3 no treatment (7 day), 2 ROCK inhibitor treated (7 day) animals were excluded due to failed intraperitoneal injection (no EdU-labelled) or mass brain bleeding during surgery. An additional 2 no treatment (4 hour), 1 ROCK inhibitor treated (4 hour), 2 no treatment (7 day), and 1 ROCK inhibitor treated (7 day) animals were excluded for SVZ quantification due to tearing of brain sections in these regions preventing quantification.<br>All other data was included in the study.
Replication	All data in this paper was replicated in at least one other independent experiment (see Source Data) except in vitro ATAC-seq library preparation (4 young biological replicates and 4 old biological replicates, combined in 1 experiment) and experiment in Extended Data Fig 10b (2 biological young and 2 biological old replicates +/- treatment, 4-5 technical replicates per biological replicate)<br>Independent replication was done for Fig 2i,j,l,m,o,p,r,s,u; Fig 3d,g,h,k,l,m,n,o,p; Fig 4e,f,g,i,j; Fig 5b,c,f,g,i,j,k; Extended Data Fig 1b; Extended Data Fig 7f-r; Extended Data Fig 8f,g; Extended Data Fig 9c,d,f,g,h,i; Extended Data Fig 10c-m
Randomization	For all figures, excepted ones noted below, mice from multiple orders from the NIA or multiple cages were used to control for covariates and experiments were performed alternating between experimental groups (either young - old or control - treatment) to avoid batch effect. For

Fig. 5, 8, block randomization was used on cages of mice such that an equal number of mice per cage were assigned to each experimental group.

Blinding

Blinding was generally not done for these figures except when noted in Methods (Fig 2i,j,l,m; Fig 6i,j; Fig 8f-h; Extended Data Fig 7f-g,j-m; Extended Data Fig 10i,j). However, all of our quantifications (with the exception of the Matrigel dispersion assays and cell type annotation in immunohistochemistry experiments which were quantified by hand) were performed in an automated fashion using software tools (e.g. Imaris for live cell imaging migration tracking, Fiji for staining quantification and counting of EdU-positive cells, CellProfiler for cell counting). We have indicated that no blinding was done for these figures in the Methods section.

Reporting for specific materials, systems and methods

We require information from authors about some types of materials, experimental systems and methods used in many studies. Here, indicate whether each material, system or method listed is relevant to your study. If you are not sure if a list item applies to your research, read the appropriate section before selecting a response.

Materials & experimental systems

n/a	Involved in the study
☐	☒ Antibodies
☒	☐ Eukaryotic cell lines
☒	☐ Palaeontology and archaeology
☐	☒ Animals and other organisms
☒	☐ Clinical data
☒	☐ Dual use research of concern

Methods

n/a	Involved in the study
☒	☐ ChIP-seq
☐	☒ Flow cytometry
☒	☐ MRI-based neuroimaging

Antibodies

Antibodies used

Primary Antibodies for FACS: CD31-PE (eBioscience, Clone 390, Cat. 12-0311-81, Lot 4338515 [1:50]), CD45-Brilliant Violet 605 (Biolegend, Clone 30-F11, Cat. 103139, Lot B264625 [1:50]), CD24-eFluor 450 (eBioscience, Clone M1/69, Cat. 48-0242-82, Lot 4311339 [1:400]), EGF-Alexa 647 (Molecular probes, Cat. E-35351, Lot 1526644 [1:300]), and Prominin-1-biotin (Invitrogen,Clone 13A4, Cat. 13-1331-82, Lot 2233571 [1:400]), ALCAM-APC (R&D, Cat. FAB1172A, lot AASL0320111 [10 uL per 10^6 cells])

Secondary Antibodies for FACS: Streptavidin- PEcy7 (eBioscience, Ref 25-4317-82, Lot 4290713 [1:1000]).

Primary antibodies used for immunofluorescence: Ki67 (Invitrogen, Clone SolA15, Cat. 14-5698-82, Lot 2196796 [1:200]), DCX (Cell Signaling Technologies, 4604, Lot 6 [1:500]), GFAP (Abcam, Cat. ab53554, Lot GR3254781-1 [1:500]), Vinculin (Abcam, Cat. ab129002, [1:200]), S100a6 (Abcam, Cat. ab181975, [1:500]), Arl13b (Abcam, Cat. ab136648, [1:500]), Phalloidin (Invitrogen, Cat. A12379, 665217, [1:500]), ALCAM/CD166 (Bio-techne, Cat. AF1172-SP, [1:40]), Paxillin (Abcam, Cat. ab32084, [1:200]), cleaved-caspase3 (Cell Signaling Technology, Cat. 9664T, [1:1000]), and NeuN (Millipore, Clone A60, Cat. MAB377, Lot 2919670, [1:500]).

Secondary antibodies used for immunofluorescence: Donkey anti-Rabbit Alexa 568 (Invitrogen, Ref A10042, Lot 2136776 [1:500]), Goat anti-Rat Alexa 647 (Invitrogen, Ref A21247, Lot 2156534 [1:500]), Donkey anti-Goat 488 (Sigma Aldrich, Clone CF488AS, SAB460032-250UL, Lot 19C1014 [1:1000]), Donkey anti-Rat 594 (Life Technologies, Ref A21209, Lot 1979379 [1:1000]), Donkey anti-mouse Alexa 568 (Life Technologies, A100, 37, Lot 2300930 [1:1000]), Donkey anti-Rabbit Alexa 647 (Invitrogen, Ref A31573, Lot 1903516 [1:1000]), Donkey anti-Goat Alexa 647 (Invitrogen, A21447, [1:1000]), and Donkey anti-Mouse 488 (Life Technologies, A21202, [1:1000]).

Validation

All antibodies were validated for the indicated applications by the manufacturer. Additionally, the following antibodies were used in mouse brain sections or primary cultures derived from mouse brains and verified in this study:
The Ki67 antibody was validated in vitro as it only stained the nucleus of cultured aNSCs/NPCs (proliferative NSCs) and not quiescent NSCs (non-proliferative NSCs). The Ki67, DCX, and GFAP antibodies were also validated in vivo by examination of sagittal brain sections: Ki67 and DCX specifically labelled cells in the SVZ neurogenic niche and along the RMS (and no other regions), and the GFAP antibody displayed stereotypic cytoplasmic and projection GFAP staining present in both Ki67-positive (aNSCs) and Ki67-negative cells (qNSCs) but no DCX+ cells (neuroblasts). Additionally, all three of these antibodies are widely cited in the literature. The Arl13b antibody was validated in vivo as it only stained cilia lining the ventricle walls. Vinculin antibody was validated in vitro and in vivo and displayed stereotypical cytoplasmic staining. Phalloidin was validated in vitro and displayed stereotypical actin staining pattern and is widely cited in the literature. Paxillin was validated in vitro and displayed stereotypical localization to focal adhesion puncta and is widely cited in the literature. Alcam/CD166 antibody was validated in vitro for species specificity and displayed typical staining pattern, matching literature reports. Cleaved-caspase 3 antibody was validated in vitro and only stained very small subset of cells and its staining pattern increased in cells treated with a chemotherapeutic drug known to induce apoptosis and is widely cited in literature. NeuN antibody was validated in vivo and only stained nuclei and it did not stain cells in the SVZ niche, but stained cells outside this region (corresponding to the natural distribution of neurons in the brain). All secondary antibodies were validated with a no primary control to ensure no non-specific staining.

Animals and other research organisms

Policy information about studies involving animals; ARRIVE guidelines recommended for reporting animal research, and Sex and Gender in Research

Laboratory animals	For in vivo ATAC-seq libraries and immunofluorescence experiments with coronal sections, male and female GFAP-GFP (FVB/N background) were used. For ATAC-seq on cultured NSCs, male and female C57BL/6 mice obtained from the NIA Aged Rodent Colony were used. For CRISPR/Cas9 experiments, make and female Rosa26-Cas9 (C57BL/6 mice) were used. For all other experiments, male C57BL/6 mice were used. Generally for all experiments, young mice were 2.5-5 months old and old mice were 18-26 months old (exact ages of mice used for specific experiments can be found in Supplementary Table 1). Mice were habituated for more than one week at Stanford before use. At Stanford, all mice were housed in either the Comparative Medicine Pavilion or the Neuro Vivarium, and their care was monitored by the Veterinary Service Center at Stanford University under IACUC protocols 8661.
Wild animals	No wild animals were used in this study.
Reporting on sex	For aging colonies generated by the lab (GFAP-GFP and Rosa26-Cas9) we used male and female mice to normalize for potential sex differences. Sex was assigned at weaning based on anogenital distance. Majority of mice obtained from NIA Aged Rodent colony are male so male mice were used for most in vitro experiments and in vivo EdU experiments. Sex of all animals used in experiments are detailed in Source Data.
Field-collected samples	No field-collected samples were used in this study.
Ethics oversight	At Stanford, all mice were housed in either the Comparative Medicine Pavilion or the Neuro Vivarium, and their care was monitored by the Veterinary Service Center at Stanford University under IACUC protocols 8661.

Note that full information on the approval of the study protocol must also be provided in the manuscript.

Flow Cytometry

Plots

Confirm that:

☒ The axis labels state the marker and fluorochrome used (e.g. CD4-FITC).

☒ The axis scales are clearly visible. Include numbers along axes only for bottom left plot of group (a 'group' is an analysis of identical markers).

☒ All plots are contour plots with outliers or pseudocolor plots.

☒ A numerical value for number of cells or percentage (with statistics) is provided.

Methodology

Sample preparation	Sorting freshly isolated populations of cells from the young and old SVZ:<br>We micro-dissected and processed the subventricular zones from young (3-5 months old) and old (20-24 months old) GFAP-GFP mice as previously described (Codega et al 2014) with the addition of negative gating for CD45 (hematopoietic lineage) and sorting of endothelial cells (CD31+) as previously described (Leeman et al 2018) (see Extended Data Fig. 1a). All FACS sorting was performed at the Stanford FACS facility on a BD Aria II sorter, using a 100-um nozzle at 13.1 pounds/square inch (psi). Due to the rarity of NSC lineage cells, we pooled sorted cells from 2 young male and 2 young female GFAP-GFP mice for the young conditions (3-5 months old), and from 3 old male and 3 old female GFAP-GFP mice for the old conditions (20-24 months old). For each respective library, we sorted either 2000 astrocytes, 2000 qNSCs (with the exception of a single library which only had 1670 cells), 800-1000 aNSCs, 2000 NPCs, or 2000 endothelial cells from GFAP-GFP animals for ATAC-seq (see Supplementary Table 2). Young and old cells of the five cell types were sorted into 150 uL of NeuroBasal-A medium (Gibco, 10888-022) with penicillin-streptomycin-glutamine (Gibco, 10378-016) and 2% B-27 minus vitamin A (Gibco, 12587-010) in a 96-well V-bottomed plate (Costar, 3894) and spun down at 300g for 5 min at 4C for downstream ATAC-seq library generation.<br><br>Sorting young and old cultured qNSCs and aNSCs/NPCs for ATAC-seq library generation:<br>To obtain primary cultures of adult NSCs for ATAC-seq, we micro-dissected and pooled SVZs from pairs of male and female C57BL/6 mouse at a young age (3 months old) or an old age (23 months old) obtained from the NIA. We dissociated and cultured NSCs as described in Methods (in the section "Primary NSC cell culture") to generate 4 young and 4 old biological replicates. At passage 5, NSCs were plated at a density of 1.2 million cells per 6cm PDL-coated plate in complete quiescent media for 8 days prior to sorting. At passage 7, NSCs from the same culture were plated at a density of 1.5 million cells per 6cm plate onto PDL-coated plates in complete activation media for 24 hours prior to sorting to synchronize quiescent and activated sorting experiments. Plates were washed 3x with 1x PBS. Adherent qNSCs were lifted from the plate using 1 mL of Accutase (STEMCELL Technologies, 07920) for 15 min at 37C and adherent aNSCs/NPCs were lifted from the plate using 1 mL of Accutase (STEMCELL Technologies, 07920) for 5min at 37C. The Accutase (STEMCELL Technologies, 07920) cell suspension was diluted with 10 mL of 1x PBS, cells were spun down at 300g for 5min, then resuspended in 200 uL of Neurobasal-A (Gibco, 10888-022) supplemented with 2% B-27 minus vitamin A (Gibco, 12587-010), 1% penicillin–streptomycin–glutamine (Gibco, 10378-016) with propidium iodide (BioLegend, 421301, 1:5000) for live/dead staining. Cells were kept on ice during all subsequent steps. Due to concern about differing levels of dead cells in the young vs. old cultures as well as the contaminating influence of dead cells on ATAC-seq libraries, all samples were sorted using fluorescence-activated cell sorting (FACS) based on the live gate (propidium iodide). Specifically, 15,000 live cultured qNSCs and cultured aNSCs/NPCs (with the exception of a single young qNSC library with 10,000 cells) (see Supplementary Table 2) were respectively sorted into 100 uL

of NeuroBasal-A medium (Gibco, 10888-022) with penicillin-streptomycin-glutamine (Gibco, 10378-016) and 2% B-27 minus vitamin A (Gibco, 12587-010) in a 96-well V-bottomed plate (Costar, 3894) and spun down at 300g for 5 min at 4C foir downstream ATAC-seq library generation.

Sorting young and old cultured aNSCs/NPCs for live cell migration tracking
NSCs were cultured as described in Methods (see Primary NSC culture) in complete activated media until Passage 2-3 and then seeded at a density of 200,000 cells per well in a PDL-coated 12-well plate with complete activated media. After 48 hours, adherent aNSCs/NPCs were passaged with 500 uL of Accutase (STEMCELL Technologies, 07920), and resuspended in Neurobasal-A (Gibco, 10888-022) supplemented with 2% B27 minus vitamin A (Gibco, 12587-010) and 1% penicillin–streptomycin–glutamine (Gibco, 10378-016) with propidium iodide (BioLegend, 421301, 1:5000) for live/dead staining. 1000 live (PI-) cells per well were FACS sorted using the BD PICI onto PDL-coated Incucyte ImageLock 96-well plates (Essen BioScience, 4379) for live cell migration tracking experiments.

Quantitative FACS of cultured primary NSCs
NSCs were cultured in complete activated media until Passage 5-6 and then passaged with Accutase (STEMCELL Technologies, 07920) and seeded at a density of 200,000 cells per well in Matrigel (Corning, 354230, Lot #0062012, diluted [1:100] in cold DMEM/F12 (Thermo Fisher Scientific, 11320033))-coated 6-well plates (Falcon, 353046) with complete activated media (for aNSCs/NPCs) or quiescent media (for qNSCs). For aNSCs/NPCs, cells were processed 24 hours after plating. For qNSCs, cells were processed after 7 days in quiescent media (replaced every other day). For the following steps, qNSCs and aNSCs/NPCs were processed in the same manner. Media was removed and 1 mL of Accutase (STEMCELL Technologies, 07920) was added to each well and incubated at 37 deg C for 5 minutes. Cells were triturated repeated with a P1000 to dissociate and lift cells and then transferred to a 15 mL conical tube pre-filled with 1 mL PBS. 1 mL of PBS was added to each well to recover any remaining cells. Cells were centrifuged at 300 rcf for 5 minutes at 4 deg C. All subsequent steps were performed on ice or at 4 deg C. Samples were decanted, and pellet was resuspended in 150 uL PBS + 2% FBS (Gibco, 10099-141) and transferred to a 96-well U-bottom plate (Falcon, 353077). This plate was then centrifuged at 400 rcf for 5 minutes at 4 deg C. After centrifugation, the plate was immediately inverted to remove supernatant and stained with ALCAM-APC (R&D, FAB1172A, lot AASL0320111 [10 uL per 10^6 cells]) resuspended in PBS + 2% FBS (Gibco, 10099-141) at 4 deg C for 30 minutes while shaking. After incubation, samples were washed with PBS + 2% FBS (Gibco, 10099-141) and centrifuged at 400 rcf for 5 minutes at 4 deg C. Next, the plate was immediately inverted, and cells were resuspended in 200 uL PBS + 2% FBS (Gibco, 10099-141) + DAPI (ThermoFisher, 62248 [1:500]) and then filtered through a cell strainer snap cap (Falcon, 352235). Unstained, single-stained, and FMO controls were also prepared. Cells were immediately run on a bioanalyzer (BD LSR II), alternating between young and old conditions to minimize batch effect. For each sample, 10,000 live cells were recorded.

Instrument

All cell sorting was performed using BD Aria II or BD PICI machine models housed in the Stanford Shared FACS Facility. All quantitative FACS was performed on a bioanalyzer (BD LSR II) housed in the Stanford Shared FACS Facility.

Software

Flowjo (v8 and v10.7.1) software was used for data analysis.

Cell population abundance

Of all live (PI-) cells dissociated from the SVZ, endothelial cells accounted for ~4-5%. OF live (PI-), lineage-restricted (CD31-/CD45-), GFAP+ cells, astrocytes accounted for ~30-40%, qNSCs accounted for ~15-20%, and aNSCs accounted for ~10%. Of live (PI-), lineage-restricted (CD31-/CD45-), GFAP- cells, NPCs accounted for ~15-20%.

The purity of this gating strategy was previously determined by RNA-sequencing to ensure that markers of other contaminating cell populations were not present in the populations being assessed (Leeman et al. 2018). We further assessed chromatin accessibility at cell marker loci within these populations to ensure that no other contaminating cell populations (based on stereotypical cell markers) were likely present in our gating strategy.

Gating strategy

Gating was determined using fluorescent-minus-one controls for each color used in each FACS experiment to ensure that positive populations were solely associated with the antibody for that specific marker.

☒ Tick this box to confirm that a figure exemplifying the gating strategy is provided in the Supplementary Information.
